# Supplementary material for: COVID-19 in Portugal: a retrospective review of paediatric cases, hospital and PICU admissions in the first pandemic year
Source: BMJ Paediatr Open. 2022 Aug 29;6(1):e001499. doi: 10.1136/bmjpo-2022-001499 (PMC9438012; doi:10.1136/bmjpo-2022-001499)
Supplement: Supplementary data [file bmjpo-2022-001499supp001.pdf]

**SUPPLEMENTARY TABLE 1. PEDIATRIC COVID-19 CASES COUNTRY OF ORIGIN, EXCLUDING PORTUGAL**

| Country | n(%) |
|---|---|
| Brasil | 2170 (59.2%) |
| Angola | 331 (9.0%) |
| Cape Verde | 205 (5.6%) |
| Saint Tome and Principe | 121 (3.3%) |
| Guine Bissau | 86 (2.3%) |
| Nepal | 75 (2.0%) |
| Ukraine | 72 (2.0%) |
| Italy | 66 (1.8%) |
| France | 62 (1.7%) |
| Venezuela | 49 (1.3%) |
| Spain | 47 (1.3%) |
| United Kingdom | 45 (1.2%) |
| Romenia | 45 (1.2%) |
| India | 33 (0.9%) |
| Bangladesh | 27 (0.7%) |
| Mozambique | 23 (0.6%) |
| Netherlands | 19 (0.5%) |
| Republic of Moldava | 17 (0.5%) |
| Germany | 16 (0.4%) |
| Paquistan | 16 (0.4%) |
| Belgium | 14 (0.4%) |
| Bulgaria | 14 (0.4%) |
| Colombia | 12 (0.3%) |
| Cuba | 9 (0.2%) |
| Ireland | 9 (0.2%) |
| China | 7 (0.2%) |
| United States of America | 7 (0.2%) |
| Other | 67 (1.8%) |

**SUPPLEMENTARY TABLE 2. SYMPTOMATIC AND ASYMPTOMATIC COVID-19 CASES PER AGE**

| Years | Asymptomatic n (%) | Symptomatic n (%) | Total | Total reported cases | Missing information n (%) |
|---|---|---|---|---|---|
| 0 | 162 (15.1%) | 913 (84.9%) | 1075 | 1719 | 644 (37.5%) |
| 1 | 387 (15.9%) | 2040 (84.1%) | 2427 | 3725 | 1298 (34.8%) |
| 2 | 485 (22.6%) | 1658 (77.4%) | 2143 | 3692 | 1549 (42%) |
| 3 | 527 (28.2%) | 1342 (71.8%) | 1869 | 3332 | 1463 (43.9%) |
| 4 | 640 (34.1%) | 1237 (65.9%) | 1877 | 3709 | 1832 (49.4%) |
| 5 | 704 (34.7%) | 1325 (65.3%) | 2029 | 4023 | 1994 (49.6%) |
| 6 | 769 (36%) | 1367 (64%) | 2136 | 4299 | 2163 (50.3%) |
| 7 | 825 (36.4%) | 1442 (63.6%) | 2267 | 4592 | 2325 (50.6%) |
| 8 | 871 (34.7%) | 1640 (65.3%) | 2511 | 5058 | 2547 (50.4%) |
| 9 | 966 (34.6%) | 1824 (65.4%) | 2790 | 5548 | 2758 (49.7%) |
| 10 | 918 (33%) | 1868 (67%) | 2786 | 5705 | 2919 (51.2%) |
| 11 | 892 (31.6%) | 1930 (68.4%) | 2822 | 5726 | 2904 (50.7%) |
| 12 | 879 (30.2%) | 2029 (69.8%) | 2908 | 5865 | 2957 (50.4%) |
| 13 | 816 (26.8%) | 2229 (73.2%) | 3045 | 6080 | 3035 (49.9%) |
| 14 | 869 (27.2%) | 2329 (72.8%) | 3198 | 6449 | 3251 (50.4%) |
| 15 | 829 (23.5%) | 2702 (76.5%) | 3531 | 6919 | 3388 (49%) |
| 16 | 778 (19.9%) | 3139 (80.1%) | 3917 | 7635 | 3718 (48.7%) |
| 17 | 760 (18.4%) | 3379 (81.6%) | 4139 | 7975 | 3836 (48.1%) |
| Total | 13077 (27.5%) | 34393 (72.4) | 47470 | 92051 | 44581 (48.4%) |

Footnote: Asymptomatic and symptomatic percentages calculated from all patients with known disease presentation; Missing information percentages calculated from all patients within an age bracket

**SUPPLEMENTARY TABLE 3. DISTRIBUITION OF SYMPTOMS OF PAEDIATIC COVID-19 CASES**

| | Fever | Cough | Rhinorrhea | Dyspneia | Headache | Diarrhoea | Abdominal pain | Odynophagia | Nausea and Vomiting | Arthalgia | Myalgia | Tiredness and weakness | Tachycardia | Chest pain | Irritability | Convulsions |
|---|---|---|---|---|---|---|---|---|---|---|---|---|---|---|---|---|
| Total* | 9737 (46.2%) | 10016 (47.5%) | 7633 (36.2%) | 800 (3.8%) | 7065 (33.5%) | 2153 (10.2%) | 1461 (6.9%) | 4958 (23.5%) | 1622 (7.7%) | 160 (0.8%) | 3552 (16.9%) | 1642 (7.8%) | 65 (0.3%) | 432 (2%) | 85 (0.4%) | 38 (0.2%) |
| Symptoms** | | | | | | | | | | | | | | | | |
| Fever | | 3721 (37,2%) | 2902 (38%) | 314 (39,3%) | 2824 (40%) | 1060 (49,2%) | 845 (57,8%) | 2046 (41,3%) | 992 (61,2%) | 81 (50,6%) | 1615 (45,5%) | 752 (45,8%) | 52 (80%) | 156 (36,1%) | 56 (65,9%) | 25 (65,8%) |
| Cough | 3721 (38,2%) | | 4203 (55,1%) | 584 (73%) | 2724 (38,6%) | 790 (36,7%) | 430 (29,4%) | 2310 (46,6%) | 562 (34,6%) | 74 (46,3%) | 1600 (45%) | 752 (45,8%) | 30 (46,2%) | 261 (60,4%) | 40 (47,1%) | 18 (42,9%) |
| Rhinorrhea | 2902 (29,8%) | 4203 (42%) | | 366 (45,8%) | 1976 (28%) | 568 (26,4%) | 342 (23,4%) | 1746 (35,2%) | 415 (25,6%) | 68 (42,5%) | 1019 (28,7%) | 565 (34,4%) | 22 (33,8%) | 144 (33,3%) | 33 (38,8%) | 11 (26,2%) |
| Dyspneia | 314 (3,2%) | 584 (5,8%) | 366 (4,8%) | | 208 (2,9%) | 77 (3,6%) | 48 (3,3%) | 183 (3,7%) | 64 (3,9%) | 9 (5,6%) | 132 (3,7%) | 118 (7,2%) | 16 (24,6%) | 102 (23,6%) | 4 (4,7%) | 1 (2,4%) |
| Headache | 2824 (29%) | 2724 (27,2%) | 1976 (25,9%) | 208 (26%) | | 616 (28,6%) | 596 (40,8%) | 2106 (42,5%) | 578 (35,6%) | 99 (61,9%) | 2055 (57,9%) | 857 (52,2%) | 14 (21,5%) | 205 (47,5%) | 16 (18,8%) | 13 (31%) |
| Diarrhoea | 1060 (10,9%) | 790 (7,9%) | 568 (7,4%) | 77 (9,6%) | 616 (8,7%) | | 513 (35,1%) | 326 (6,6%) | 430 (26,5%) | 23 (14,4%) | 355 (10%) | 220 (13,4%) | 4 (6,2%) | 45 (10,4%) | 16 (18,8%) | 6 (14,3%) |
| Abdominal pain | 845 (8,7%) | 430 (4,3%) | 342 (4,5%) | 48 (6%) | 596 (8,4%) | 513 (23,8%) | | 351 (7,1%) | 436 (26,9%) | 24 (15%) | 252 (7,1%) | 159 (9,7%) | 11 (16,9%) | 49 (11,3%) | 10 (11,8%) | 5 (11,9%) |
| Odynophagia | 2046 (21%) | 2310 (23,1%) | 1746 (22,9%) | 183 (22,9%) | 2106 (29,8%) | 326 (15,1%) | 351 (24%) | | 319 (19,7%) | 68 (42,5%) | 1080 (30,4%) | 460 (28%) | 16 (24,6%) | 135 (31,3%) | 15 (17,6%) | 12 (28,6%) |
| Nausea and Vomiting | 992 (10,2%) | 562 (5,6%) | 415 (5,4%) | 64 (8%) | 578 (8,2%) | 430 (20%) | 436 (29,8%) | 319 (6,4%) | | 24 (15%) | 296 (8,3%) | 228 (13,9%) | 13 (20%) | 55 (12,7%) | 16 (18,8%) | 5 (11,9%) |
| Arthalgia | 81 (0,8%) | 74 (0,7%) | 68 (0,9%) | 9 (1,1%) | 98 (1,4%) | 23 (1,1%) | 24 (1,6%) | 68 (1,4%) | 24 (1,5%) | | 115 (3,2%) | 56 (3,4%) | 1 (1,5%) | 13 (3%) | 3 (3,5%) | 0 (0%) |
| Myalgia | 1615 (16,6%) | 1600 (16%) | 1019 (13,3%) | 132 (16,5%) | 2055 (29,1%) | 355 (16,5%) | 252 (17,2%) | 1080 (21,8%) | 296 (18,2%) | 115 (71,9%) | | 648 (39,5%) | 13 (20%) | 124 (28,7%) | 10 (11,8%) | 6 (14,3%) |
| Tiredness and weakness | 752 (7,7%) | 752 (7,5%) | 565 (7,4%) | 118 (14,8%) | 857 (12,1%) | 220 (10,2%) | 159 (10,9%) | 460 (9,3%) | 228 (14,1%) | 56 (35%) | 648 (18,2%) | | 10 (15,4%) | 85 (19,7%) | 20 (23,5%) | 3 (7,1%) |
| Tachycardia | 52 (0,5%) | 30 (0,3%) | 22 (0,3%) | 16 (2%) | 14 (0,2%) | 4 (0,2%) | 10 (0,7%) | 16 (0,3%) | 13 (0,8%) | 1 (0,6%) | 13 (0,4%) | 10 (0,6%) | | 4 (0,9%) | 5 (5,9%) | 0 (0%) |
| Chest pain | 156 (1,6%) | 261 (2,6%) | 144 (1,9%) | 102 (12,8%) | 202 (2,9%) | 45 (2,1%) | 49 (3,4%) | 135 (2,7%) | 55 (3,4%) | 13 (8,1%) | 124 (3,5%) | 85 (5,2%) | 4 (6,2%) | | 1 (1,2%) | 0 (0%) |
| Irritability | 56 (0,6%) | 40 (0,4%) | 33 (0,4%) | 4 (0,5%) | 16 (0,2%) | 16 (0,7%) | 10 (0,7%) | 15 (0,3%) | 15 (0,9%) | 3 (1,9%) | 10 (0,3%) | 20 (1,2%) | 5 (7,7%) | 1 (0,2%) | | 3 (7,1%) |
| Convulsions | 25 (0,3%) | 18 (0,2%) | 11 (0,1%) | 1 (0,1%) | 13 (0,2%) | 6 (0,3%) | 5 (0,3%) | 12 (0,2%) | 5 (0,3%) | 0 (0%) | 6 (0,2%) | 3 (0,2%) | 0 (0%) | 0 (0%) | 3 (3,5%) | |

Footnote: *Percentages calculated from symptomatic patients with specified symptoms, **Percentages calculated from patients with column symptom
