# Supplementary material for: COVID-19 in Portugal: a retrospective review of paediatric cases, hospital and PICU admissions in the first pandemic year
Source: BMJ Paediatr Open. 2022 Aug 29;6(1):e001499. doi: 10.1136/bmjpo-2022-001499 (PMC9438012; doi:10.1136/bmjpo-2022-001499)
Supplement: Author's manuscript [file bmjpo-2022-001499.draft_revisions.pdf]

BMJ Paediatrics Open

BMJ Paediatrics Open is committed to open peer review. As part of this commitment we make the peer review history of every article we publish publicly available.

When an article is published we post the peer reviewers' comments and the authors' responses online. We also post the versions of the paper that were used during peer review. These are the versions that the peer review comments apply to.

The versions of the paper that follow are the versions that were submitted during the peer review process. They are not the versions of record or the final published versions. They should not be cited or distributed as the published version of this manuscript.

BMJ Paediatrics Open is an open access journal and the full, final, typeset and author-corrected version of record of the manuscript is available on our site with no access controls, subscription charges or pay-per-view fees (http://bmjpaedsopen.bmj.com).

If you have any questions on BMJ Paediatrics Open's open peer review process please email info.bmjpo@bmj.com

BMJ Paediatrics Open

COVID-19 in Portugal: a retrospective review of paediatric cases, hospital and PICU admissions in the first pandemic year

Journal:	*BMJ Paediatrics Open*
Manuscript ID	bmjpo-2022-001499
Article Type:	Original research
Date Submitted by the Author:	31-Mar-2022
Complete List of Authors:	Elias, Cecilia; Universidade de Lisboa, EPI Task- Force FMUL; USP Francisco George<br>Feteira-Santos, Rodrigo; Universidade de Lisboa, EPI Task-Force FMUL ; Instituto de Medicina Preventiva e Saúde Pública, Faculdade de Medicina, Universidade de Lisboa. , Avenida Professor Egas Moniz, 1649-028, Lisboa, Portugal.<br>Camarinha, Catarina; Universidade de Lisboa, EPI Task-Force FMUL<br>de Araújo Nobre, Miguel; Universidade de Lisboa, EPI Task-Force FMUL; FMUL, Clinica Universitaria Estomatologia<br>Costa, Andreia Silva; Universidade de Lisboa, EPI Task-Force FMUL; Universidade de Lisboa Instituto de Saúde Ambiental<br>Bacelar-Nicolau, Leonor; Universidade de Lisboa, EPI Task-Force FMUL; Universidade de Lisboa, FMUL - Institute of Preventive Medicine & ISAMB<br>Furtado, Cristina; Universidade de Lisboa, EPI Task-Force FMUL; Instituto Nacional de Saúde Doutor Ricardo Jorge<br>Nogueira, Paulo Jorge; Universidade de Lisboa, EPI Task- Force FMUL; Universidade de Lisboa Faculdade de Medicina, Instituto de Medicina Preventiva e Saúde Pública
Keywords:	COVID-19, Epidemiology, Mortality

SCHOLARONE™
Manuscripts

I, the Submitting Author has the right to grant and does grant on behalf of all authors of the Work (as defined in the below author licence), an exclusive licence and/or a non-exclusive licence for contributions from authors who are: i) UK Crown employees; ii) where BMJ has agreed a CC-BY licence shall apply, and/or iii) in accordance with the terms applicable for US Federal Government officers or employees acting as part of their official duties; on a worldwide, perpetual, irrevocable, royalty-free basis to BMJ Publishing Group Ltd ("BMJ") its licensees and where the relevant Journal is co-owned by BMJ to the co-owners of the Journal, to publish the Work in this journal and any other BMJ products and to exploit all rights, as set out in our licence.

The Submitting Author accepts and understands that any supply made under these terms is made by BMJ to the Submitting Author unless you are acting as an employee on behalf of your employer or a postgraduate student of an affiliated institution which is paying any applicable article publishing charge ("APC") for Open Access articles. Where the Submitting Author wishes to make the Work available on an Open Access basis (and intends to pay the relevant APC), the terms of reuse of such Open Access shall be governed by a Creative Commons licence – details of these licences and which Creative Commons licence will apply to this Work are set out in our licence referred to above.

Other than as permitted in any relevant BMJ Author's Self Archiving Policies, I confirm this Work has not been accepted for publication elsewhere, is not being considered for publication elsewhere and does not duplicate material already published. I confirm all authors consent to publication of this Work and authorise the granting of this licence.

Confidential: For Review Only

Article

COVID-19 in Portugal: a retrospective review of paediatric cases, hospital and PICU admissions in the first pandemic year

Cecília Elias [1,2], Rodrigo Feteira-Santos [1,3,4,5], Catarina Camarinha [1], Miguel de Araújo Nobre [1,3,6], Andreia Silva Costa [1,5,7,8], Leonor Bacelar-Nicolau [1,3,4,5], Cristina Furtado [1,3,5,9], Paulo Jorge Nogueira [1,3,4,5*]

1. EPI Task-Force FMUL, Faculdade de Medicina, Universidade de Lisboa. Avenida Professor Egas Moniz, 1649-028, Lisboa, Portugal.

2. Unidade de Saúde Publica Francisco George, ACES Lisboa Norte, ARSLVT

3. Instituto de Medicina Preventiva e Saúde Pública, Faculdade de Medicina, Universidade de Lisboa. Avenida Professor Egas Moniz, 1649-028, Lisboa, Portugal.

4. Área Disciplinar Autónoma de Bioestatística (Laboratório de Biomatemática), Faculdade de Medicina, Universidade de Lisboa. Avenida Professor Egas Moniz, 1649-028, Lisboa, Portugal.

5. Instituto de Saúde Ambiental, Faculdade de Medicina, Universidade de Lisboa. Avenida Professor Egas Moniz, 1649-028, Lisboa, Portugal.

6. Clínica Universitária de Estomatologia, Faculdade de Medicina, Universidade de Lisboa. Avenida Professor Egas Moniz, 1649-028 Lisboa, Portugal.

7. CIDNUR - Centro de Investigação, Inovação e Desenvolvimento em Enfermagem de Lisboa Escola Superior de Enfermagem de Lisboa. Avenida Professor Egas Moniz, 1600-190 Lisboa.

8. CRC-W—Católica Research Centre for Psychological, Family and Social Wellbeing, Universidade Católica Portuguesa, Palma de Cima, 1649-023 Lisboa, Portugal.

9. National Institute of Health Dr. Ricardo Jorge, Av. Padre Cruz, 1600-560 Lisboa, Portugal.

* **Correspondence**: cecilia.elias@gmail.com

Keywords: COVID-19, Pediatric, Epidemiology, Comorbidities, Hospital Admission, PICU

Confidential: For Review Only

ABSTRACT

Background: COVID-19 is considered by WHO a pandemic with public health emergency repercussions. Children often develop a mild disease with good prognosis. The prompt recognition of children at risk is essential to successfully manage pediatric COVID-19.

Methods: Data on all reported pediatric COVID-19 cases, in Portugal, was retrospectively assessed from a fully anonymized dataset provided by the Directorate-General for Health (DGS). Pediatric hospital admission results were attained from the DGS vaccine recommendations and pediatric intensive care unit (PICU) admission results from the Epicentre.pt work group.

Results: 92051 COVID-19 cases were studied, 50.5% males, average age of 10.1 years. The most common symptoms were cough, fever, rhinorrhea and headache. Gastrointestinal symptoms were infrequent. Patients with comorbidities were more likely symptomatic with cough (41.9%), fever (32.9%), or headache (26.6%). Dyspnea had a 12-fold increase, tachycardia 11-fold and convulsions 10-fold in the comorbidity group. The most common comorbidity was asthma (n=753). Hospital admission was required in 0.93% of cases and PICU admission in 3.48 per 10000 cases. PICU admission for Multisystem Inflammatory Syndrome in Children was observed more frequently in children with no comorbidities (75%) and males, whereas severe COVID-19 was rarer and occurred mainly in female patients and under 1 year of age. Overall children with comorbidities were 26 times more likely to require ICU admission. Two deaths were reported as caused by COVID-19. Case fatality rate and mortality rates were extremely low, 1.8 per 100,000 cases and 1.2 per 1,000,000 respectively.

Conclusions: The clinical presentations observed were predominantly respiratory and neurologic, gastrointestinal symptoms were infrequent. Symptomatic presentations were strikingly frequent in the subpopulation with comorbidities and at a greater risk of PICU admission. Less than 1% of patients required hospital admission. MIS-C was more frequent in patients with no comorbidities and males. Mortality and case-fatality rates in Portugal were extremely low.

Keywords: COVID-19, Pediatric, Epidemiology, Comorbidities, MIS-C, Mortality

Confidential: For Review Only

Confidential: For Review Only

What we know about this subject:

Children present a milder course of disease and a lower susceptibility to being severely affected by COVID-19. However, children with comorbidities can present worse clinical condition and outcomes. Globally pediatric COVID-19 case-fatality and mortality rates are low.

What our study adds:

Portuguese children with comorbidities are highly symptomatic for COVID-19 and in particular can develop dyspnoea 12x, tachycardia 11x and convulsions 10x more frequently than children without comorbidities. MIS-C was observed more frequently in male patients and those without comorbidities whereas severe COVID-19 was observed more frequently in extremely young (<1year) female patients. Children with comorbidities were 26x more likely to require PICU admission. Portuguese children and adolescents presented an extremely low mortality and case-fatality rate, 1.8 per 100,000 cases and 1.2 per 1,000,000 respectively.

Confidential: For Review Only

BACKGROUND

Coronavirus disease 2019 (COVID-19), caused by severe acute respiratory syndrome coronavirus 2 (SARS-CoV-2), is considered by the World Health Organization (WHO) a pandemic with public health emergency repercussions.[1],[2] Worldwide, millions of people have been infected with COVID-19 since the end of 2019 among all age groups.[3] Children are also at risk for COVID-19 but have experienced a lower incidence than other age groups.[4],[5] Laboratory-confirmed COVID-19 cases in the United States reveal that only 1.7% of cases were among children and adolescents under 18 years old.[6] Furthermore, several studies showed these age groups have lower susceptibility to being severely affected, often developing a mild form of disease and with a likely good prognosis.[7–9] Studies have shown that children present different symptoms [10], distinct comorbidities, decreased disease severity and fewer disease complications.[10] The characteristics and clinical manifestations of infected children[11],[12] and the role of the pediatric population in SARS-CoV-2 spread in the community have been considered of major interest.[13] The most frequent symptoms described in children have been fever, cough[14] and ear, nose, and throat (ENT) complaints. However, other manifestations such as gastrointestinal symptoms have also been identified.[12],[15],[16] A small number of children need hospital admission, and of these, a minority required ICU admission either due to severe COVID-19 or multisystem inflammatory disease in children (MIS-C). Several risk factors have been associated with these conditions such as extreme age, male sex, comorbidities and non-white background.[17]

As COVID-19 characteristics and clinical course among children can differ from adult patients in numerous ways, specific recommendations for the diagnosis, treatment and vaccination plans in children are being implement worldwide, despite differences across countries.[18],[19],[20],[21]

Increasing knowledge of the clinical characteristics in this demographic group may also contribute towards the development and continuous adaptation of preventive strategies that can benefit all communities.

This study aims to investigate the epidemiological and clinical characteristics of pediatric SARS-CoV-2 infection cases, hospital and ICU admissions in Portugal and compare these to other countries.

MATERIALS AND METHODS

Confidential: For Review Only

Study design and data

Data on all cases of COVID-19 in children and adolescents under the age of 18, in Portugal, diagnosed between March 2nd, 2020, and February 28th, 2021 was retrospectively assessed. The dataset was fully anonymized and was provided by the Directorate-General for Health (DGS) on11th of March 2021. The data was collected by the SINAVE (National Epidemiological Surveillance System). Hospital admissions with a SARS-CoV-2 primary or secondary diagnosis, from March 2nd to December 31st 2020, were obtained as results from the Pediatric COVID-19 vaccination technical recommendation by DGS.[22] No information on whether the diagnosis was the primary or secondary cause of admission was available. Data on pediatric SARS-CoV-2 PICU admissions were obtained as anonymized data results on severe COVID-19 and Multisystem inflammatory syndrome in children (MIS-C) from the Epicentre.pt work group. Data from the National Statistics Institute (INE) was used for mortality and population comparisons. The pediatric mid 2020 population was used by proxy as an estimate population for the period analyzed. Patients and public were not involved in the design or analysis developed in this study. According to the WHO definition, a confirmed SARS-CoV-2 case was based on a positive polymerase chain reaction test.[23] Daily death reports published by DGS were analyzed to calculate the number of deaths caused by COVID-19.

Statistical analysis

Descriptive statistics were used to summarize univariate characteristics. Results were presented as absolute and relative frequencies for qualitative variables, and as means, medians, standard deviations (SD), interquartile range (IQR), minimum and maximum statistics for quantitative variables. All analyses were conducted using R software version 3.6.3 (R Foundation for Statistical Computing, Vienna, Austria).

Results

GENERAL CHARACTERISTICS

The present study included 92051 COVID-19 cases in children and adolescents reported from March 2nd, 2020, to February 28th, 2021, as infected with SARS-CoV-2 in Portugal; 46453 (50.5%) were males, and 45583 (49.5%) females. The age profile of patients was distributed between 0 and 17 years old,

Confidential: For Review Only

with case numbers increasing with age (Table 1). An average age of 10.1 (standard deviation of 5.0) and a median of 11.0 years were observed.

The geographical distribution highlights most cases occurred in 3 urban areas, 22999 (25%) in Lisbon district, followed by Porto 18350 (19.9%) and Braga 11091 (12%). The areas with fewer cases were predominantly rural districts (Portalegre and Beja) and autonomous regions of the Azores and Madeira islands (Figure 1). The children and adolescents with COVID-19 had mostly Portuguese nationality (95.3%). Only 4% were from other backgrounds, mainly from Brazil, Angola, Cape Verde, and São Tome and Principe scattered from a wide range of countries.

Confidential: For Review Only

COVID-19 CASES TIMELINE

During this study, several COVID-19 epidemic waves were observed (Figure 2): 1) between March and April 2020, when 738 cases were reported (0.8% of all cases); 2) between May and July 2020, when 1756 cases (1.9% of all cases) were reported; 3) from September to December 2020, when 39897 cases (43.3% of all cases) were reported; and 4) from January to March 2021, following lockdown easing during the Christmas season, when 46634 cases (50.7% of all cases) were reported.

CLINICAL PRESENTATION

Symptoms were reported in 21077 (22.9%) of the cases; 13466 (14.6%) were recorded as asymptomatic, and there was no information regarding the presence or absence of symptoms stated in 57508 cases (62.5%). The number of reported symptoms and the frequency of COVID-19 cases were inversely proportional, 7089 (7.7%) presented one symptom, whereas 89 patients (0.1%) presented with eight or more symptoms. The asymptomatic presentation of the disease increased with age, peaking between 8 and 12 years of age and slightly decreasing subsequently.

The most common symptoms observed individually were cough (10205, 11.1%), fever (9812, 10.7%), rhinorrhea (7739, 8.4%), and headache (7143, 7.8%). Analyzing the distribution of the co-occurrence of clinical COVID-19 symptoms, in each pediatric patient, fever was commonly combined with cough

(38.1%), rhinorrhea (29.7%), headache (28.9%), odynophagia (20.9%) or myalgia (16.5%). Furthermore, combinations of these symptoms were the most common presentations observed. Fever, cough, and rhinorrhea were the most common presentations for children from 0 to 9 (Table 2). Children aged 0 presented with the highest rates of fever (30%), cough (23%), rhinorrhea (22%) and diarrhea (6.3%) when compared to the other age groups. From age 10 to 17 years, headache was the most common reported symptom (12%), followed by fever, cough, rhinorrhea, odynophagia, and myalgia.

COMORBIDITIES

The majority (98%) of pediatric patients diagnosed with COVID-19 had no reported comorbidities. A total of 1593 patients presented with comorbidities, of which 1000 were specified. Of these, 943 patients presented with one comorbidity, 47 presented with two comorbidities and 10 presented with three or more comorbidities. The three most frequent comorbidities associated with COVID-19 in children and adolescents were: asthma (n = 753, 0.8%), neuromuscular diseases (n = 49) and diabetes (n = 55). Most patients with asthma or neurologic disease were males, whereas most patients with diabetes were females. (Table 3)

Patients with comorbidities presented symptoms more frequently than patients with no comorbidities. Fever was observed in 524 of these patients (32.9%) whereas 9812 (10.7%) patients with no comorbidities, corresponding to approximately 3-fold increase. Similarly, 41.9% of patients with comorbidities presented with cough, a 3-fold increase; 10.3% with dyspnea, a 12-fold increase; 26.6% with headache, a 3-fold increase and 6.8% with abdominal pain, a 4-fold increase.

HOSPITAL AND PICU ADMISSIONS AND DEATHS

A total of 476 children were admitted to hospital with a COVID-19 ICD-10 code, either as primary or secondary diagnosis. Children aged from 0 – 4 represented 47.9% (228) of hospital admissions, corresponding to 2.24% of all Covid-19 cases in this age group during this time. (Table 4) No other sociodemographic or clinical variables were available to further characterize this population.

Confidential: For Review Only

A total of 32 children required PICU admission, of these 28 (87.5%) were admitted due to MIS-C and 4 (12.5%) due to severe COVID-19. MIS-C patients were predominantly male (21, 75%) with ages from 15 months to 17 years age, average of 10.75 and median of 10.5 years. Most cases occurred between January and February 2021. Six patients presented comorbidities. No deaths or major disability were observed.

Severe COVID-19 was observed mainly in female patients, under the age of one and all presenting comorbidities.

The risk of PICU admission in patients with comorbidities was 26x increased when comparing to patients without comorbidities.

Two deaths caused by COVID-19 were reported by the DGS during this study.

.

Confidential: For Review Only

Discussion

In this study, epidemiological and clinical characteristics of pediatric COVID-19 infections, hospital and PICU admissions in Portugal were described. These results highlight the clinical differences of COVID-19 presentations in the various pediatric ages, the most common combinations of symptoms and the role comorbidities play.

Although there is accessible and exhaustive knowledge regarding adult COVID-19 disease[24],[25] pediatric COVID-19 epidemiology and clinical characteristics are significantly less studied and documented in the literature. SARS-CoV-2 infection studies have shown that children are more likely to be asymptomatic than adults [26] and present a less severe disease course.[27] [28] Young children and infants are reported as more vulnerable to infection [29] than older pediatric patients. COVID-19 related fatalities have been reported as scarce in the pediatric population.[6] In this context, we assessed the characteristics and clinical features of COVID-19 in children in Portugal.

We observed an increase of COVID-19 cases with age in both sexes with slightly higher number of cases in males compared to females (50.5% vs. 49.5%). However, when comparing within age groups, significant differences between sex were observed mainly between 16 and 17, and predominantly in females. Overall, in the literature, male sex seems to be most affected, although individual smaller studies have shown either sex as being the most frequent.[9],[28]

Most of these cases are geographic clustered in the high-density urban areas of Lisbon, Porto, and Braga. The association between population density and cumulative infection cases spread was previously observed at a county level in the United States of America and considered an effective predictor of cumulative infection cases.[30] Though the regions with the lower incidences were mainly rural and low-density populated districts, such as Portalegre, Beja and the autonomous regions of the Azores and Madeira islands. The nationality of COVID-19 pediatric patients reflects the population's background. The majority are Portuguese and 4% come from diverse backgrounds Brazil, Angola, Cape Verde, and São Tome and Principe, countries with historical ties and emigration patterns to Portugal.

Several COVID-19 waves were observed in our study. Interestingly, the pediatric and adult population peaks co-occurred simultaneously. Following the first wave, in March and April 2020, the first State of

Confidential: For Review Only

Emergency [31], and lockdown were implemented, including school closures, mandatory remote work, limiting mobility, business hours, and social gatherings.

Between March 17th and April 2nd, all children who attended the Emergency Department (ED) of a Portuguese hospital with suspected COVID-19 symptoms as cough, fever or rhinorrhoea were tested. Of all 94 cases, one was positive.[32] This can indicate a low incidence of disease at this stage which is in accordance with our results. Studies on antibody seroprevalence of SARS-CoV-2 have shown that only half the children and adolescents had developed disease symptoms.[33]

The presence of gastrointestinal symptoms has been identified as major symptoms occurring in the pediatric population.[34],[35],[36] In our analysis, diarrhea, abdominal pain, nausea and vomiting were less frequent than other symptoms like cough, fever, rhinorrhea, and headache, such has been reported elsewhere.[4],[28] These symptoms have been dominant in other meta-analysis describing clinical characteristics of children: Wang et al. reported the presence of fever in 48% and cough 39% of the cases.[14] Li et al.[37] in 7004 pediatric cases reported fever 47% and cough 42% as the most prevalent clinical symptoms. As found in our results (38.1%), the combination of symptoms, fever and cough 30% has also been documented.[37] Interestingly the most common symptoms per age groups can be organized in 3 sets: 1) from ages zero to four years: fever, cough and rhinorrhea; 2) from ages five to nine years: fever, cough, rhinorrhea and headache; and 3) from 10 to 17 years: the previous 4 symptoms, odynophagia and myalgia. This result can reflect simultaneously the inability of young children to express their complaints and an increase in the variety of symptoms and presentations as age increases. Unknown clinical presentations were frequent and while the majority of these are likely to be asymptomatic patients in which no symptoms were denied on the digital platform SINAVE, during contact tracing, future optimization of this platform is important to mitigate these limitations. Concomitantly work overload and non-medical personal on the contact tracing team might also help explain these results.

Asthma was the most common underlying condition (753 cases) and has been the most reported comorbidity among several pediatric cases as well.[6],[5],[28] However, this represents a lower prevalence of asthma than would have been anticipated by the authors suggesting asthma was underreported or these patients were successfully shielding at this phase of the pandemic. Verma et al.

Confidential: For Review Only

analysed SARS-CoV-2 infections in children in four hospitals in the New York City area and found those with asthma were more likely to need respiratory support during hospitalization.[38] In a systematic review, including 5686 children aged under 18 years with SARS-CoV-2 infection, the most frequent comorbidity was cardiac disease; and most children who required ventilation had underlying comorbidities.[4] Only a small proportion of children with COVID-19 had comorbidities (n = 1000, 2%) which is in keeping with other studies.[28] However, overall comorbidity prevalence is lower than the authors would expect; which is likely associated with typical adult diseases, rather than paediatric diseases, being available as tick box in the SINAVE platform. Further to this, a free text box, where contact tracing team could add other comorbidities, was not made available.

A study of ED and admitted pediatric COVID-19 patients at a Lisbon Hospital showed 10% had risk factors for disease, 20% were asymptomatic, 43% presented with fever and 42% with respiratory symptoms.[39] Though the referred study is likely representing the most symptomatic COVID-19 cases seeking medical help, whereas our study focuses on all pediatric COVID-19 cases, chronic respiratory disease was also the most common comorbidity.

Children and adolescents with comorbidities reported a higher number of symptoms per patient and were less likely asymptomatic. These results are in line with studies showing children with comorbidities report worse clinical presentations and outcome.[40] Furthermore, all symptoms were reported more often and remarkably dyspnoea was approximately 12 times more common, tachycardia 11 times and convulsions 10 times, symptoms which could be associated with disease severity. These results imply children and adolescents with comorbidities, once diagnosed with COVID-19, should receive thorough medical follow-up and care.

In 2020, a total of 476 children were admitted to hospital from 51153 cases reported, corresponding to 1 in 108 cases requiring hospitalization, and a hospitalization rate of 0.93% of cases nationwide. A much lower result than reported in neighbouring Navarra, Spain (3.6%) during the first wave[41] or in the United States (5.3%)[42] and in Scotland, where 1.03% of cases in children aged 5- to 17-year-olds required hospitalization[43]. These results probably reflect a higher testing rate of asymptomatic or mildly ill children in Portugal.

Confidential: For Review Only

Thirty-two cases required PICU admission, corresponding to a rate of admission of 3.48 per 10000 cases. Four cases (females under the age of one year and presenting comorbidities; 12.5%) were admitted with severe COVID-19 (non-MIS-C) and twenty-eight (87.5%) with MIS-C, implying that in our population, severe COVID-19 in children as less frequent than MIS-C. Patients with MIS-C were predominantly male and the majority (75%) had no comorbidities, which is similar to previously published results, where most MIS-C patients were healthy, and no correlation was observed between comorbidity score and MIS-C occurrence.[44]

During this period, the Directorate-General of Health confirmed two paediatric deaths caused by COVID-19, corresponding to a case-fatality rate of approximately 1.8 per 100,000 cases. Furthermore, analysing specifically total paediatric deaths in Portugal in 2020 (n=387) and comparing to the paediatric COVID-19 deaths, COVID-19 was the main cause of death of 0.52%. The mortality rate due to COVID-19 was 1.2 per million children and adolescents. These results find parallel to similar studies, highlighting the low mortality and case-fatality rates. Despite the extremely small number of deaths, it is reasonable to compare these results with other countries. Smith et al.[45] reported a mortality rate due to COVID-19 of 2 per million and fatality rate of 5 per 100,000 in England, between March 2020 and February 2021, a result 1.7 and 2.5 times higher than the one we report, respectively.[43] Spanish mortality results were published by Tagarro et al.[46] and reported 0.21 per 100 000 in children aged 0 to 9 years and 0.34 per 100 000 in children aged 10 to 19 years. These results may reflect differences in shielding of vulnerable population or lockdown policies implemented in these countries, but further studies are required to analyse these effects.

The major strengths of our study are the sample size and the robust analysis of the large paediatric database, allowing an extensive description of clinical and epidemiological characteristics.

The authors found unusual the lack of surveillance data in the analyzed dataset. The analysis of real-world data has important challenges as previously acknowledged[47], however other studies using national data have reported similar difficulties[48],[49]. Despite the authors best effort in this analysis, less than optimal real-world data has imposed important limitations to the study and hindered some of its quality, particularly relating to missing values in some of the analyzed variables. The authors are

Confidential: For Review Only

developing further studies and proposing solutions to help prevent these issues. As stated, the major limitations of this analysis are data-related, the possible underreporting of cases and its characteristics, such as symptoms and comorbidities and the impracticality of establishing a chronological order of events. Furthermore, the estimated population used for comparison was obtain by-proxy with the reported paediatric for 2020.

CONCLUSIONS

COVID-19 affects all age groups, although pediatric patients tend to develop a milder course of disease associated with a good prognosis. The clinical manifestations of disease observed in our analyses were predominantly respiratory and neurologic, while gastrointestinal symptoms were reported infrequently. The small fraction of pediatric COVID-19 patients who present with comorbidities reported strikingly increased symptomatic presentations compared to patients without comorbidities and present a higher risk of PICU admission. Less than 1% of patients required hospital admission. MIS-C was more frequent in patients with no comorbidities and males, severe COVID-19 was rarer occurred mainly in children under 1 year of age and females, all with comorbidities. Case fatality rates and mortality rates were extremely low when comparing with other countries. Further studies on the impact of COVID-19 in patients with comorbidities and on factors associated with hospitalization and death in healthy children are required.

Confidential: For Review Only

Acknowledgements

The authors would like to thank Dr Cristina Camilo and the EPICENTRE.PT research group.

Funding

This research received no funding.

Conflict of Interests

The authors declare no conflict of interest.

Contributorship Statement

Cecilia Elias planned the study and was the guarantor. She developed the methodology and was involved in obtaining the data, data analysis, writing the manuscript, creating tables/figures and reviewing the text. Rodrigo Feteira-Santos was involved in obtaining data, writing the manuscript, creating tables and figures and reviewing the text. Catarina Camarinho was involved in planning the study, creating tables and figures, writing and reviewing the manuscript and maintaining references. Miguel de Araújo Nobre was involved in writing and reviewing the manuscript. Andreia Costa was involved in writing and reviewing the manuscript. Leonor Bacelar was involved in writing and reviewing the manuscript. Cristina Furtado was involved in writing and reviewing the manuscript. Paulo Nogueira was involved in planning the study, data analysis and writing and reviewing the manuscript.

Confidential: For Review Only

References:

[1] Cucinotta D, Vanelli M. WHO Declares COVID-19 a Pandemic. Acta Biomed 2020;91:157–60. https://doi.org/10.23750/abm.v91i1.9397.

[2] WHO/Europe | Coronavirus disease (COVID-19) outbreak - WHO announces COVID-19 outbreak a pandemic n.d.

[3] Wu Z, McGoogan JM. Characteristics of and Important Lessons From the Coronavirus Disease 2019 (COVID-19) Outbreak in China: Summary of a Report of 72 314 Cases From the Chinese Center for Disease Control and Prevention. JAMA 2020;323:1239–42. https://doi.org/10.1001/jama.2020.2648.

[4] Williams N, Radia T, Harman K, Agrawal P, Cook J, Gupta A. COVID-19 Severe acute respiratory syndrome coronavirus 2 (SARS-CoV-2) infection in children and adolescents: a systematic review of critically unwell children and the association with underlying comorbidities. Eur J Pediatr 2021;180:689–97. https://doi.org/10.1007/s00431-020-03801-6.

[5] Stokes EK, Zambrano LD, Anderson KN, Marder EP, Raz KM. Coronavirus Disease 2019 Case Surveillance — United States, January 22–May 30, 2020. MMWR Morb Mortal Wkly Rep 2020 2020;69:759–65.

[6] Bialek S, Gierke R, Hughes M, McNamara LA, Pilishvili T, Skoff T. Coronavirus disease 2019 in children: Current status - Morbidity and Mortality Weekly Report 2020;69:422–6.

[7] Dong Y, Mo X, Hu Y, Qi X, Jiang F, Jiang Z, et al. Epidemiology of COVID-19 Among Children in China. Pediatrics 2020;145:20200702. https://doi.org/10.1542/PEDS.2020-0702.

[8] Mantovani A, Rinaldi E, Zusi C, Beatrice G, Saccomani MD, Dalbeni A. Coronavirus disease 2019 (COVID-19) in children and/or adolescents: a meta-analysis. Pediatr Res 2021;89:733–7. https://doi.org/10.1038/s41390-020-1015-2.

[9] Götzinger F, Santiago-García B, Noguera-Julián A, Lanaspa M, Lancella L, Calò Carducci FI, et al. COVID-19 in children and adolescents in Europe: a multinational, multicentre cohort study. Lancet Child Adolesc Heal 2020;4:653–61. https://doi.org/10.1016/S2352-4642(20)30177-2.

[10] Zare-Zardini H, Soltaninejad H, Ferdosian F, Hamidieh AA, Memarpoor-Yazdi M. Coronavirus Disease 2019 (COVID-19) in Children: Prevalence, Diagnosis, Clinical Symptoms, and Treatment. Int J Gen Med 2020;13:477–82. https://doi.org/10.2147/IJGM.S262098.

[11] Guo CX, He L, Yin JY, Meng XG, Tan W, Yang GP, et al. Epidemiological and clinical features of pediatric COVID-19. BMC Med 2020;18:1–7. https://doi.org/10.1186/s12916-020-01719-2.

[12] Zimmermann P, Curtis N. Coronavirus infections in children including COVID-19: An overview

Confidential: For Review Only

of the epidemiology, clinical features, diagnosis, treatment and prevention options in children. Pediatr Infect Dis J 2020;39:355–68. https://doi.org/10.1097/INF.0000000000002660.

[13] Davies NG, Klepac P, Liu Y, Prem K, Jit M, Pearson CAB, et al. Age-dependent effects in the transmission and control of COVID-19 epidemics. Nat Med 2020;26:1205–11. https://doi.org/10.1038/s41591-020-0962-9.

[14] Wang Z, Zhou Q, Wang C, Shi Q, Lu S, Ma Y, et al. Clinical characteristics of children with COVID-19: a rapid review and meta-analysis. Ann Transl Med 2020;8:620. https://doi.org/10.21037/atm-20-3302.

[15] Mayor S. Covid-19: UK studies find gastrointestinal symptoms are common in children. BMJ 2020;370. https://doi.org/10.1136/bmj.m3484.

[16] Giacomet V, Barcellini L, Stracuzzi M, Longoni E, Folgori L, Leone A, et al. Gastrointestinal symptoms in severe covid-19 children. Pediatr Infect Dis J 2020;39:E317–20. https://doi.org/10.1097/INF.0000000000002843.

[17] Ward JL, Harwood R, Smith C, Kenny S, Clark M, Davis PJ, et al. Risk factors for PICU admission and death among children and young people hospitalized with COVID-19 and PIMS-TS in England during the first pandemic year. Nat Med 2022;28:193–200. https://doi.org/10.1038/s41591-021-01627-9.

[18] Maltezou HC, Vorou R, Papadima K, Kossyvakis A, Spanakis N, Gioula G, et al. Transmission dynamics of SARS-CoV-2 within families with children in Greece: A study of 23 clusters. J Med Virol 2021;93:1414–20. https://doi.org/10.1002/jmv.26394.

[19] Han Y-N, Feng Z-W, Sun L-N, Ren X-X, Wang H, Xue Y-M, et al. A comparative-descriptive analysis of clinical characteristics in 2019-coronavirus-infected children and adults. J Med Virol 2020;92:1596–602. https://doi.org/10.1002/jmv.25835.

[20] Bi Q, Wu Y, Mei S, Ye C, Zou X, Zhang Z, et al. Epidemiology and transmission of COVID-19 in 391 cases and 1286 of their close contacts in Shenzhen, China: a retrospective cohort study. Lancet Infect Dis 2020;20:911–9. https://doi.org/10.1016/S1473-3099(20)30287-5.

[21] Kawasuji H, Takegoshi Y, Kaneda M, Ueno A, Miyajima Y, Kawago K, et al. Transmissibility of COVID-19 depends on the viral load around onset in adult and symptomatic patients. PLoS One 2020;15:e0243597. https://doi.org/10.1371/journal.pone.0243597.

[22] Direção Geral da Saúde (DGS). Comissão Técnica de Vacinação contra a COVID-191 Vacinação contra a COVID-19 em crianças com 5 a 11 anos n.d.

[23] World Health Organization. Laboratory testing for 2019 novel coronavirus (2019-nCoV) in suspected human cases. WHO - Interim Guid 2020;2019:1–7.

Confidential: For Review Only

[24] Lechien JR, Chiesa-Estomba CM, Place S, Van Laethem Y, Cabaraux P, Mat Q, et al. Clinical and epidemiological characteristics of 1420 European patients with mild-to-moderate coronavirus disease 2019. J Intern Med 2020;288:335–44. https://doi.org/https://doi.org/10.1111/joim.13089.

[25] Zhou F, Yu T, Du R, Fan G, Liu Y, Liu Z, et al. Clinical course and risk factors for mortality of adult inpatients with COVID-19 in Wuhan, China: a retrospective cohort study. Lancet 2020. https://doi.org/10.1016/S0140-6736(20)30566-3.

[26] Adeyinka A, Bailey K, Pierre L, Kondamudi N. COVID 19 infection: Pediatric perspectives. J Am Coll Emerg Physicians Open 2021;2:1–11. https://doi.org/10.1002/emp2.12375.

[27] Dong Y, Dong Y, Mo X, Hu Y, Qi X, Jiang F, et al. Epidemiology of COVID-19 among children in China. Pediatrics 2020;145. https://doi.org/10.1542/peds.2020-0702.

[28] Patel NA. Pediatric COVID-19: Systematic review of the literature. Am J Otolaryngol - Head Neck Med Surg 2020;41:102573. https://doi.org/10.1016/j.amjoto.2020.102573.

[29] Dong Y, Mo X, Hu Y, Qi X, Jiang F, Jiang Z, et al. Epidemiology of COVID-19 Among Children in China. Pediatrics 2020;145. https://doi.org/10.1542/peds.2020-0702.

[30] Wong DWS, Li Y. Spreading of COVID-19: Density matters. PLoS One 2020;15:e0242398.

[31] Diário da República 1.ª série. Decreto do Presidente da República n.º 14-A/2020 de 18 de março. 2020.

[32] Costa A, Almeida H, Moniz M, Alves C. COVID-19 screening in a Portuguese pediatric population. Enferm Infecc Microbiol Clin 2020:9–12. https://doi.org/10.1016/j.eimc.2020.09.004.

[33] Felsenstein S, Hedrich CM. SARS-CoV-2 infections in children and young people. Clin Immunol 2020;220:108588. https://doi.org/https://doi.org/10.1016/j.clim.2020.108588.

[34] Waterfield T, Watson C, Moore R, Ferris K, Tonry C, Watt A, et al. Seroprevalence of SARS-CoV-2 antibodies in children: a prospective multicentre cohort study. Arch Dis Child 2021;106:680–6. https://doi.org/10.1136/archdischild-2020-320558.

[35] Akobeng AK, Grafton-Clarke C, Abdelgadir I, Twum-Barimah E, Gordon M. Gastrointestinal manifestations of COVID-19 in children: a systematic review and meta-analysis. Frontline Gastroenterol 2021;12:332–7. https://doi.org/10.1136/flgastro-2020-101529.

[36] Al-Beltagi M, Saeed NK, Bediwy AS, El-Sawaf Y. Paediatric gastrointestinal disorders in SARS-CoV-2 infection: Epidemiological and clinical implications. World J Gastroenterol 2021;27:1716–27. https://doi.org/10.3748/wjg.v27.i16.1716.

[37] Li B, Zhang S, Zhang R, Chen X, Wang Y, Zhu C. Epidemiological and Clinical Characteristics

Confidential: For Review Only

of COVID-19 in Children: A Systematic Review and Meta-Analysis. Front Pediatr 2020;8:591132. https://doi.org/10.3389/fped.2020.591132.

[38] Verma S, Lumba R, Dapul HM, Gold-von Simson G, Phoon CK, Lighter JL, et al. Characteristics of Hospitalized Children With SARS-CoV-2 in the New York City Metropolitan Area. Hosp Pediatr 2021;11:71–8. https://doi.org/10.1542/hpeds.2020-001917.

[39] Picão de Carvalho C, Castro C, Sampaio Graça I, Lorenzo C, Barbosa Rodrigues A, Inácio R, et al. Série de Casos de 103 Crianças com Infeção por SARS-CoV-2 em Portugal. Acta Med Port 2020;33:795. https://doi.org/10.20344/amp.14537.

[40] Tsankov BK, Allaire JM, Irvine MA, Lopez AA, Sauvé LJ, Vallance BA, et al. Severe COVID-19 Infection and Pediatric Comorbidities: A Systematic Review and Meta-Analysis. Int J Infect Dis 2021;103:246–56. https://doi.org/10.1016/j.ijid.2020.11.163.

[41] Moreno-Galarraga L, Urretavizcaya-Martínez M, Alegría Echauri J, García Howard M, Ruperez García E, Aguilera-Albesa S, et al. SARS-CoV-2 infection in children requiring hospitalization: the experience of Navarra, Spain. World J Pediatr 2020;16:614–22. https://doi.org/10.1007/s12519-020-00393-x.

[42] Kim TY, Kim EC, Agudelo AZ, Friedman L. COVID-19 hospitalization rate in children across a private hospital network in the United States: COVID-19 hospitalization rate in children. Arch Pediatr 2021;28:530–2. https://doi.org/10.1016/j.arcped.2021.07.004.

[43] Shi T, Pan J, Katikireddi SV, McCowan C, Kerr S, Agrawal U, et al. Risk of COVID-19 hospital admission among children aged 5–17 years with asthma in Scotland: a national incident cohort study. Lancet Respir Med 2022. https://doi.org/10.1016/S2213-2600(21)00491-4.

[44] Preston LE, Chevinsky JR, Kompaniyets L, Lavery AM, Kimball A, Boehmer TK, et al. Characteristics and Disease Severity of US Children and Adolescents Diagnosed With COVID-19. JAMA Netw Open 2021;4:e215298–e215298. https://doi.org/10.1001/jamanetworkopen.2021.5298.

[45] Smith C, Odd D, Harwood R, Ward J, Linney M, Clark M, et al. Deaths in children and young people in England after SARS-CoV-2 infection during the first pandemic year. Nat Med 2022;28:185–92. https://doi.org/10.1038/s41591-021-01578-1.

[46] Tagarro A, García-Salido A, Martínez E-V, Vega-Piris L, Mellado MJ. Low COVID-19 mortality in Spanish children. Lancet Child Adolesc Heal 2021;5:e24–5. https://doi.org/10.1016/S2352-4642(21)00125-5.

[47] Liu M, Qi Y, Wang W, Sun X. Toward a better understanding about real-world evidence. Eur J Hosp Pharm Sci Pract 2022;29:8–11. https://doi.org/10.1136/ejhpharm-2021-003081.

Confidential: For Review Only

[48] Nogueira PJ, de Araújo Nobre M, Costa A, Ribeiro RM, Furtado C, Bacelar Nicolau L, et al. The Role of Health Preconditions on COVID-19 Deaths in Portugal: Evidence from Surveillance Data of the First 20293 Infection Cases. J Clin Med 2020;9. https://doi.org/10.3390/jcm9082368.

[49] Nogueira PJ, de Araújo Nobre M, Costa A, Ribeiro RM, Furtado C, Bacelar Nicolau L, et al. Reply to Comment on Nogueira, P.J., et al. "The Role of Health Preconditions on COVID-19 Deaths in Portugal: Evidence from Surveillance Data of the First 20293 Infection Cases". J. Clin. Med. 2020, 9, 2368. J Clin Med 2020;9. https://doi.org/10.3390/jcm9113449.

Confidential: For Review Only

TABLE 1. AGE AND SEX OF PEDIATRIC COVID-19 INFECTED CASES, DURING 2020 IN PORTUGAL.

		Total n (%)***	Female n (%)**	Male n (%)**
n		92051	45583 (49.5%)	46453 (50.5%)
Age	0	1719 (1.9%)	826 (48.1%)	884 (51.9%)
	1	3725 (4.0%)	1784 (47.9%)	1940 (52.1%)
	2	3692 (4.0%)	1860 (50.4%)	1832 (49.6%)
	3	3332 (3.6%)	1575 (47.3%)	1757 (52.7%)
	4	3709 (4.0%)	1840 (49.6%)	1869 (50.4%)
	Subtotal 1-4 y	14458 (15.7%)	7059 (48.8%)	7398 (51.2%)
	5	4023 (4.4%)	1988 (49.4%)	2035 (50.6%)
	6	4299 (4.7%)	2128 (49.5%)	2171 (50.5%)
	7	4592 (5.0%)	2232 (48.6%)	2360 (51.4%)
	8	5058 (5.5%)	2490 (49.2%)	2568 (50.8%)
	9	5548 (6.0%)	2709 (48.8%)	2839 (51.2%)
	Subtotal 5-9 y	23520 (25.6%)	11547 (49.1%)	11973 (50.9%)
	10	5705 (6.2%)	2872 (50.3%)	2833 (49.7%)
	11	5726 (6.2%)	2838 (49.6%)	2888 (50.4%)
	12	5865 (6.4%)	2928 (49.9%)	2937 (50.1%)
	13	6080 (6.6%)	2942 (48.4%)	3137 (51.6%)
	14	6449 (7.0%)	3235 (50.2%)	3213 (49.8%)
	Subtotal 10-14 y	29825 (32.4%)	14815 (49.7%)	15008 (50.3%)
	15	6919 (7.5%)	3402 (49.2%)	3514 (50.8%)
	16	7635 (8.3%)	3890 (50.9%)	3744 (49.1%)
	17	7975 (8.7%)	4041 (50.7%)	3932 (49.3%)
	Subtotal 15-17 y	22529 (24.5%)	11333 (50.3%)	11190 (49.7%)

Footnotes: 15 (0.02%) missing values were observed for sex, ** percentage within sex level; *** global percentage.

Confidential: For Review Only

TABLE 2. SYMPTOMS ASSOCIATED WITH COVID-19 PER AGE GROUP

		Age groups				
		0-1 years n (%)	1 to 4 years n (%)	5 to 9 years n (%)	10 to 14 years n (%)	15 to 17 years n (%)
Symptoms	Fever	517 (30.1%)	2870 (19.9%)	2113 (9.0%)	2338 (9.9%)	1974 (8.4%)
	Cough	401 (23.3%)	2423 (16.8%)	1933 (8.2%)	2661 (11.3%)	1913 (8.1%)
	Rhinorrhoea	379 (22.0%)	2074 (14.3%)	1484 (6.3%)	1961 (8.3%)	1841 (7.8%)
	Dyspnea	34 (2.0%)	196 (1.4%)	136 (0.6%)	181 (0.8%)	258 (1.1%)
	Headache	2 (0.1%)	151 (1.0%)	1364 (5.8%)	2761 (11.7%)	2865 (12.2%)
	Diarrhea	108 (6.3%)	632 (4.4%)	452 (1.9%)	532 (2.3%)	468 (2.0%)
	Abdominal pain	8 (0.5%)	202 (1.4%)	560 (2.4%)	461 (2.0%)	256 (1.1%)
	Odynophagia	13 (0.8%)	370 (2.6%)	1009 (4.3%)	1767 (7.5%)	1842 (7.8%)
	Nausea and vomiting	38 (2.2%)	365 (2.5%)	409 (1.7%)	488 (2.1%)	348 (1.5%)
	Arthralgia	0 (0.0%)	10 (0.1%)	26 (0.1%)	68 (0.3%)	62 (0.3%)
	Myalgia	1 (0.1%)	86 (0.6%)	443 (1.9%)	1313 (5.6%)	1752 (7.4%)
	General weakness	16 (0.9%)	154 (1.1%)	225 (1.0%)	539 (2.3%)	729 (3.1%)
	Tachycardia	2 (0.1%)	21 (0.1%)	16 (0.1%)	19 (0.1%)	13 (0.1%)

Confidential: For Review Only

TABLE 3. COMORBIDITIES OF THE PEDIATRIC COVID-19 INFECTED CASES, DURING 2020 IN PORTUGAL.

		Total<br>n (%) ***	Female<br>n (%) **	Male<br>n (%) **
Number of comorbidities	0	46859 (97.9%)	23282 (49.7%)	23577 (50.3%)
	1	943 (2.0%)	401 (42.5%)	542 (57.5%)
	2	47 (0.1%)	20 (42.6%)	27 (57.4%)
	3 or more	10 (0.02%)	6 (60.0%)	4 (40.0%)
Comorbidities	Asthma	753 (75.3%)	437 (58.0%)	316 (42.0%)
	Chronic neurologic / neuromuscular disease	49 (4.9%)	25 (51.0%)	24 (49.0%)
	Diabetes	55 (5.5%)	26 (47.3%)	29 (52.7%)
	Chronic hematological disease	40 (4.0%)	23 (57.5%)	17 (42.5%)
	Chronic neurologic deficit	34 (3.4%)	19 (55.9%)	15 (44.1%)
	Cancer	36 (3.6%)	23 (63.9%)	13 (36.1%)
	Chronic pulmonary disease	42 (4.2%)	25 (59.5%)	17 (40.5%)
	Chronic renal disease	28 (2.8%)	13 (46.4%)	15 (53.6%)
	HIV and other immunodeficiencies	25 (2.5%)	13 (52.0%)	12 (48.0%)
	Liver disease	8 (0.8%)	6 (75.0%)	2 (25.0%)

Footnotes: Results of each morbidity were presented considering the sample size of cases with any reported morbidity (n=1000).

Confidential: For Review Only

TABLE 4. PEDIATRIC HOSPITAL AND PICU ADMISSIONS

Hospital admissions from March to December 2020			
Age Group	Hospital admissions n (%)	Cases per age group	% per group
0 - 4	228 (47.9)	10161	2.24
5 - 11	112 (23.5)	18358	0.61
12 - 17	136 (28.6)	22674	0.60
Total	476 (100)	51193	0.93

PICU admissions from March 2020 to February 2021			
	Severe COVID-19	MIS-C	Total ICU admissions
n (%)	4 (12.5)	28 (87.5)	32 (100)
Reported cases	92051	92051	92051
per 10000 cases	0,43	3,04	3,48
Female n (%)	3 (75)	7 (25)	
Male n (%)	1 (25)	21 (75)	
Min age (months)	3	15	
Max age (years)	17	17	
Median (years)	0	10,5	
Comorbidities	4 (100)	6 (21.4)	10

iew Only

Confidential: For Review Only

2

Confidential: For Review Only

FIGURE 1. MAP OF COVID-19 PEDIATRIC CASES IN PORTUGAL, BY DISTRICT IN ABSOLUTE VALUE.

Confidential: For Review Only

FIGURE 2. PEDIATRIC COVID-19 CASES PER WEEK

Footnote: 1. First COVID-19 patient diagnosed in Portugal; 2. First national state of emergency; 3. Lockdown easing; 4. Preschool and primary school reopening; 5. End of the school year; 6. Start of the school year; 7. State of emergency with restrictions; 8. Christmas with minimal gathering restrictions; 9. Reintroduction of mandatory remote working, travel restrictions and school closures; 10. Highest daily death toll due to COVID-19 during the pandemic.

Confidential: For Review Only

Confidential: For Review Only

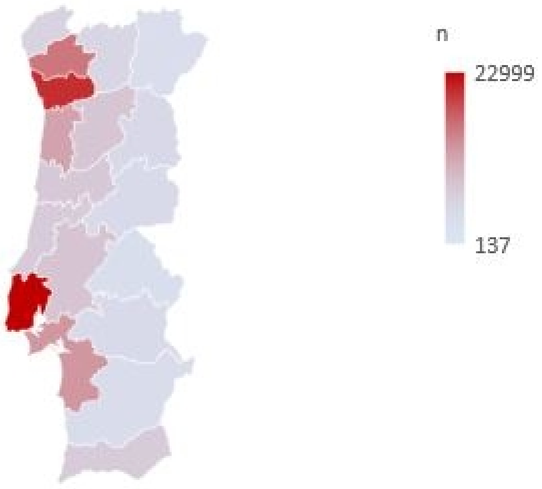

60x56mm (120 x 120 DPI)

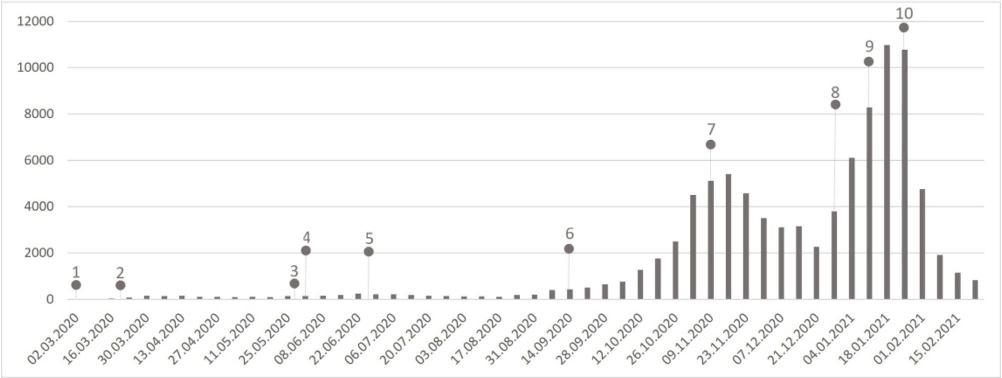

216x82mm (300 x 300 DPI)

SUPPLEMETARY TABLE 1. PEDIATRIC COVID-19 CASES COUNTRY OF ORIGIN, DURING 2020 IN PORTUGAL.

Country	n (%)
Brasil	2170 (59.2%)
Angola	331 (9%)
Cape Verde	205 (5.6%)
Saint Tome and Príncipe	121 (3.3%)
Guine Bissau	86 (2.3%)
Nepal	75 (2%)
Ukraine	72 (2%)
Italy	66 (1.8%)
France	62 (1.7%)
Venezuela	49 (1.3%)
Spain	47 (1.3%)
United Kingdom	45 (1.2%)
Romenia	45 (1.2%)
India	33 (0.9%)
Bangladesh	27 (0.7%)
Mozambique	23 (0.6%)
Netherlands	19 (0.5%)
Republic of Moldava	17 (0.5%)
Germany	16 (0.4%)
Paquistan	16 (0.4%)
Belgium	14 (0.4%)
Bulgaria	14 (0.4%)
Colombia	12 (0.3%)
Cuba	9 (0.2%)
Ireland	9 (0.2%)
China	7 (0.2%)
United States of America	7 (0.2%)
Other	67 (1.8%)

For Review Only

SUPPLEMENTARY TABLE 2. AGE AND GENDER OF SYMPTOMATIC AND ASYMPTOMATIC COVID-19 CASES

Age	Male		Male A/S ratio	Female		Female A/S ratio
	Asymptomatic n (%)	Symptomatic n (%)		Asymptomatic n (%)	Symptomatic n (%)	
0	85 (0.6%)	366 (1.7%)	4.31	83 (0.6%)	315 (1.5%)	3.80
1	211 (1.6%)	791 (3.8%)	3.75	197 (1.5%)	691 (3.3%)	3.51
2	252 (1.9%)	589 (2.8%)	2.34	255 (1.9%)	554 (2.6%)	2.17
3	274 (2.0%)	478 (2.3%)	1.74	269 (2.0%)	379 (1.8%)	1.41
4	353 (2.6%)	371 (1.8%)	1.05	305 (2.3%)	365 (1.7%)	1.20
5	349 (2.6%)	422 (2.0%)	1.21	372 (2.8%)	399 (1.9%)	1.07
6	396 (2.9%)	427 (2.0%)	1.08	387 (2.9%)	368 (1.7%)	0.95
7	420 (3.1%)	430 (2.0%)	1.02	420 (3.1%)	404 (1.9%)	0.96
8	473 (3.5%)	447 (2.1%)	0.95	411 (3.1%)	453 (2.1%)	1.10
9	503 (3.7%)	509 (2.4%)	1.01	488 (3.6%)	496 (2.4%)	1.02
10	497 (3.7%)	522 (2.5%)	1.05	455 (3.4%)	547 (2.6%)	1.20
11	492 (3.7%)	540 (2.6%)	1.10	422 (3.1%)	566 (2.7%)	1.34
12	474 (3.5%)	579 (2.7%)	1.22	435 (3.2%)	588 (2.8%)	1.35
13	446 (3.3%)	664 (3.2%)	1.49	399 (3.0%)	653 (3.1%)	1.64
14	466 (3.5%)	663 (3.1%)	1.42	425 (3.2%)	721 (3.4%)	1.70
15	453 (3.4%)	806 (3.8%)	1.78	403 (3.0%)	853 (4.0%)	2.12
16	440 (3.3%)	892 (4.2%)	2.03	365 (2.7%)	1088 (5.2%)	2.98
17	450 (3.3%)	967 (4.6%)	2.15	341 (2.5%)	1174 (5.6%)	3.44

Footnotes: A, Asymptomatic; S, Symptomatic.

Confidential: For Review Only

SUPPLEMENTARY TABLE 3. DISTRIBUTION OF COMBINATIONS OF SYMPTOMS OF PEDIATRIC COVID-19 CASES

Symptoms	Fever	Cough	Rhinorrhea	Dyspneia	Headache	Diarrhoea	Abdominal pain	Odynophagia	Nausea and Vomitng	Arthalgia	Myalgia	Tiredness and weakness	Tachycardia	Chest pain	Irritability	Convulsions
Total	9812 (10.7%)	10205 (11.1%)	7739 (8.4%)	805 (0.9%)	7143 (7.8%)	2142 (2.3%)	1487 (1.6%)	5001 (5.4%)	1648 (1.8%)	166 (0.2%)	3595 (3.9%)	3595 (1.8%)	71 (0.1%)	439 (0.5%)	88 (0.1%)	42 (0%)
Fever	-	3743 (36.7%)	2915 (37.7%)	315 (39.1%)	2837 (39.7%)	1064 (49.7%)	847 (57%)	2055 (41.1%)	993 (60.3%)	82 (49.4%)	1621 (45.1%)	753 (20.9%)	53 (74.6%)	156 (35.5%)	56 (63.6%)	25 (59.5%)
Cough	3743 (38.1%)	-	4233 (54.7%)	587 (72.9%)	2740 (38.4%)	797 (37.2%)	433 (29.1%)	2322 (46.4%)	563 (34.2%)	74 (44.6%)	1609 (44.8%)	752 (20.9%)	33 (46.5%)	262 (59.7%)	40 (45.5%)	18 (42.9%)
Rhinorrhea	2915 (29.7%)	4233 (41.5%)	-	587 (72.9%)	1987 (27.8%)	576 (26.9%)	345 (23.2%)	1759 (35.2%)	417 (25.3%)	68 (41%)	1028 (28.6%)	567 (15.8%)	23 (32.4%)	145 (33%)	33 (37.5%)	11 (26.2%)
Dyspneia	315 (3.2%)	587 (5.8%)	368 (4.8%)	-	209 (2.9%)	78 (3.6%)	48 (3.2%)	184 (3.7%)	65 (3.9%)	9 (5.4%)	132 (3.7%)	118 (3.3%)	16 (22.5%)	102 (23.2%)	4 (4.5%)	1 (2.4%)
Headache	2837 (28.9%)	2740 (26.8%)	1987 (25.7%)	209 (26%)	-	618 (28.9%)	599 (40.3%)	2115 (42.3%)	582 (35.3%)	99 (59.6%)	2064 (57.4%)	861 (23.9%)	15 (21.1%)	205 (46.7%)	16 (18.2%)	13 (31%)
Diarrhoea	1064 (10.8%)	797 (7.8%)	576 (7.4%)	78 (9.7%)	618 (8.7%)	-	518 (34.8%)	330 (6.6%)	434 (26.3%)	23 (13.9%)	355 (9.9%)	224 (6.2%)	4 (5.6%)	45 (10.3%)	16 (18.2%)	6 (14.3%)
Abdominal pain	847 (8.6%)	433 (4.2%)	345 (4.5%)	48 (6%)	599 (8.4%)	518 (24.2%)	-	353 (7.1%)	445 (27%)	25 (15.1%)	252 (7%)	163 (4.5%)	11 (15.5%)	49 (11.2%)	10 (11.4%)	5 (11.9%)
Odynophagia	2055 (20.9%)	2322 (22.8%)	1759 (22.7%)	184 (22.9%)	2115 (29.6%)	330 (15.4%)	353 (23.7%)	-	321 (19.5%)	68 (41%)	1084 (30.2%)	461 (12.8%)	16 (22.5%)	137 (31.2%)	15 (17%)	12 (28.6%)
Nausea and vomiting	993 (10.1%)	563 (5.5%)	417 (5.4%)	65 (8.1%)	582 (8.1%)	434 (20.3%)	445 (29.9%)	321 (6.4%)	-	25 (15.1%)	296 (8.2%)	233 (6.5%)	13 (18.3%)	55 (12.5%)	16 (18.2%)	5 (11.9%)
Arthalgia	82 (0.8%)	74 (0.7%)	68 (0.9%)	9 (1.1%)	99 (1.4%)	23 (1.1%)	25 (1.7%)	68 (1.4%)	25 (1.5%)	-	115 (3.2%)	57 (1.6%)	1 (1.4%)	13 (3%)	3 (3.4%)	0 (0%)
Myalgia	1621 (16.5%)	1609 (15.8%)	1028 (13.3%)	132 (16.4%)	2064 (28.9%)	355 (16.6%)	252 (16.9%)	1084 (21.7%)	296 (18%)	115 (69.3%)	-	649 (18.1%)	13 (18.3%)	124 (28.2%)	10 (11.4%)	6 (14.3%)
Tiredness and weakness	753 (7.7%)	752 (7.4%)	567 (7.3%)	118 (14.7%)	861 (12.1%)	224 (10.5%)	163 (11%)	461 (9.2%)	233 (14.1%)	57 (34.3%)	649 (18.1%)	-	10 (14.1%)	85 (19.4%)	20 (22.7%)	3 (7.1%)
Tachycardia	53 (0.5%)	33 (0.3%)	23 (0.3%)	16 (2%)	15 (0.2%)	4 (0.2%)	11 (0.7%)	16 (0.3%)	13 (0.8%)	1 (0.6%)	13 (0.4%)	10 (0.3%)	-	4 (0.9%)	5 (5.7%)	0 (0%)
Chest pain	156 (1.6%)	262 (2.6%)	145 (1.9%)	102 (12.7%)	205 (2.9%)	45 (2.1%)	49 (3.3%)	137 (2.7%)	55 (3.3%)	13 (7.8%)	124 (3.4%)	85 (2.4%)	4 (5.6%)	-	1 (1.1%)	0 (0%)
Irritability	56 (0.6%)	40 (0.4%)	33 (0.4%)	4 (0.5%)	16 (0.2%)	16 (0.7%)	10 (0.7%)	15 (0.3%)	16 (1%)	3 (1.8%)	10 (0.3%)	20 (0.6%)	5 (7%)	1 (0.2%)	-	3 (7.1%)
Convulsions	25 (0.3%)	18 (0.2%)	11 (0.1%)	1 (0.1%)	13 (0.2%)	6 (0.3%)	5 (0.3%)	12 (0.2%)	5 (0.3%)	0 (0%)	6 (0.2%)	3 (0.1%)	0 (0%)	0 (0%)	3 (3.4%)	-

Confidential: For Review Only

SUPPLEMENTARY TABLE 4. FREQUENCY OF REPORTED SYMPTOMS BY ALL PATIENTS AND PATIENTS WITH COMORBIDITIES

Clinical Features	Patients with comorbidities		All patients		Symptom ratio C/A
	n	%	n	%	
Fever	524	32.9	9812	10.7	3.1
Cough	668	41.9	10205	11.1	3.8
Rhinorrhea	444	27.9	7739	8.4	3.3
Dyspnea	167	10.5	805	0.9	12.0
Headache	423	26.6	7143	7.8	3.4
Diarrhea	111	7.0	2142	2.3	3.0
Abdominal pain	109	6.8	1487	1.6	4.2
Odynophagia	301	18.9	5001	5.4	3.55
Nausea and vomiting	121	7.6	1648	1.8	4.2
Arthralgia	20	1.3	166	0.2	7.0
Myalgia	216	13.6	3595	3.9	3.5
Tiredness and weakness	144	9.0	1163	1.8	5.0
Tachycardia	14	0.9	71	0.1	11.4
Chest pain	53	3.3	439	0.5	7.0
Irritability	13	0.8	88	0.1	8.5
Convulsions	7	0.4	42	0.0	9.6

Footnotes: C, patients with comorbidities; A, All patients.

Confidential: For Review Only

SUPPLEMETARY TABLE 5. NUMBER OF SYMPTOMS REPORTED BY PEDIATRIC COVID-19 CASES WITH AND WITHOUT COMORBIDITIES

		Studied population n (%)	Subpopulation of patients with comorbidities n (%)
Number of reported symptoms	0	13466 (14.6%)	424 (26.6%)
	1	7089 (7.7%)	224 (14.1%)
	2	6944 (7.5%)	321 (20.2%)
	3	4565 (5.0%)	304 (19.1%)
	4	2179 (2.4%)	165 (10.4%)
	5	937 (1.0%)	86 (5.4%)
	6	390 (0.4%)	41 (2.6%)
	7	126 (0.1%)	15 (0.9%)
	8 +	89 (0.1%)	12 (0.8%)

Confidential: For Review Only

Confidential: For Review Only

BMJ Paediatrics Open

COVID-19 in Portugal: a retrospective review of paediatric cases, hospital and PICU admissions in the first pandemic year

Journal:	*BMJ Paediatrics Open*
Manuscript ID	bmjpo-2022-001499.R1
Article Type:	Original research
Date Submitted by the Author:	27-May-2022

Complete List of Authors:	Elias, Cecilia; Universidade de Lisboa, EPI Task- Force FMUL; USP Francisco George<br>Feteira-Santos, Rodrigo; Universidade de Lisboa, EPI Task-Force FMUL ; Instituto de Medicina Preventiva e Saúde Pública, Faculdade de Medicina, Universidade de Lisboa. , Avenida Professor Egas Moniz, 1649-028, Lisboa, Portugal.<br>Camarinha, Catarina; Universidade de Lisboa, EPI Task-Force FMUL<br>de Araújo Nobre, Miguel; Universidade de Lisboa, EPI Task-Force FMUL; FMUL, Clinica Universitaria Estomatologia<br>Costa, Andreia Silva; Universidade de Lisboa, EPI Task-Force FMUL; Universidade de Lisboa Instituto de Saúde Ambiental<br>Bacelar-Nicolau, Leonor; Universidade de Lisboa, EPI Task-Force FMUL; Universidade de Lisboa, FMUL - Institute of Preventive Medicine & ISAMB<br>Furtado, Cristina; Universidade de Lisboa, EPI Task-Force FMUL; Instituto Nacional de Saúde Doutor Ricardo Jorge<br>Nogueira, Paulo Jorge; Universidade de Lisboa, EPI Task- Force FMUL; Universidade de Lisboa Faculdade de Medicina, Instituto de Medicina Preventiva e Saúde Pública

Keywords:	COVID-19, Epidemiology, Mortality

SCHOLARONE™
Manuscripts

I, the Submitting Author has the right to grant and does grant on behalf of all authors of the Work (as defined in the below author licence), an exclusive licence and/or a non-exclusive licence for contributions from authors who are: i) UK Crown employees; ii) where BMJ has agreed a CC-BY licence shall apply, and/or iii) in accordance with the terms applicable for US Federal Government officers or employees acting as part of their official duties; on a worldwide, perpetual, irrevocable, royalty-free basis to BMJ Publishing Group Ltd ("BMJ") its licensees and where the relevant Journal is co-owned by BMJ to the co-owners of the Journal, to publish the Work in this journal and any other BMJ products and to exploit all rights, as set out in our licence.

The Submitting Author accepts and understands that any supply made under these terms is made by BMJ to the Submitting Author unless you are acting as an employee on behalf of your employer or a postgraduate student of an affiliated institution which is paying any applicable article publishing charge ("APC") for Open Access articles. Where the Submitting Author wishes to make the Work available on an Open Access basis (and intends to pay the relevant APC), the terms of reuse of such Open Access shall be governed by a Creative Commons licence – details of these licences and which Creative Commons licence will apply to this Work are set out in our licence referred to above.

Other than as permitted in any relevant BMJ Author's Self Archiving Policies, I confirm this Work has not been accepted for publication elsewhere, is not being considered for publication elsewhere and does not duplicate material already published. I confirm all authors consent to publication of this Work and authorise the granting of this licence.

Confidential: For Review Only

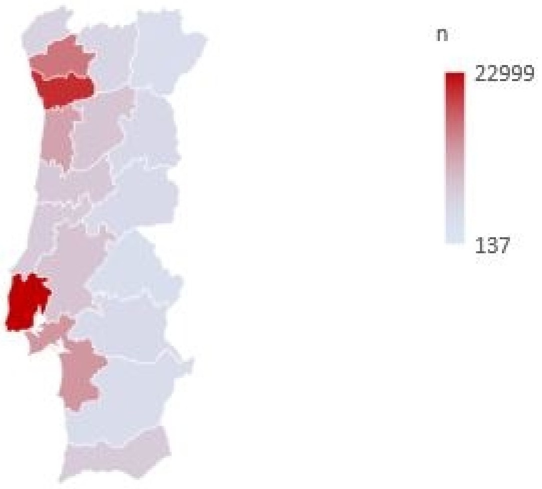

60x56mm (120 x 120 DPI)

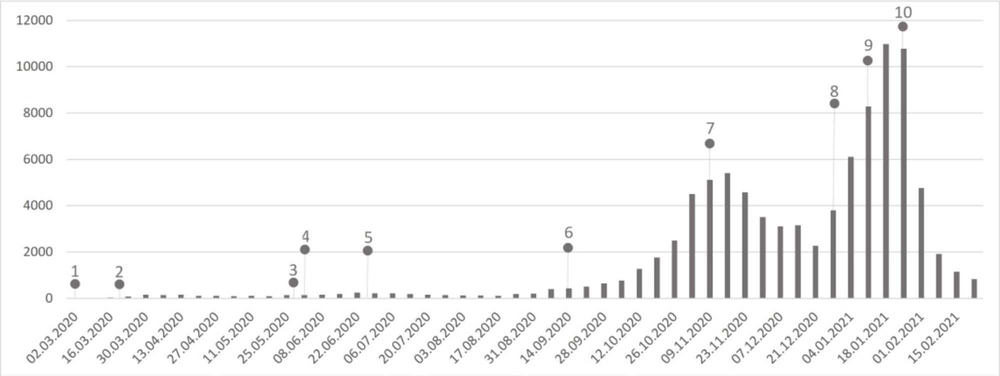

216x82mm (330 x 330 DPI)

Article

COVID-19 in Portugal: a retrospective review of paediatric cases, hospital and PICU admissions in the first pandemic year

Cecília Elias [1,2*], Rodrigo Feteira-Santos [1,3,4,5], Catarina Camarinha [1], Miguel de Araújo Nobre [1,3,6], Andreia Silva Costa [1,5,7,8], Leonor Bacelar-Nicolau [1,3,4,5], Cristina Furtado [1,3,5,9], Paulo Jorge Nogueira [1,3,4,5]

1. EPI Task-Force FMUL, Faculdade de Medicina, Universidade de Lisboa. Avenida Professor Egas Moniz, 1649-028, Lisboa, Portugal.

2. Unidade de Saúde Publica Francisco George, ACES Lisboa Norte, ARSLVT

3. Instituto de Medicina Preventiva e Saúde Pública, Faculdade de Medicina, Universidade de Lisboa. Avenida Professor Egas Moniz, 1649-028, Lisboa, Portugal.

4. Área Disciplinar Autónoma de Bioestatística (Laboratório de Biomatemática), Faculdade de Medicina, Universidade de Lisboa. Avenida Professor Egas Moniz, 1649-028, Lisboa, Portugal.

5. Instituto de Saúde Ambiental, Faculdade de Medicina, Universidade de Lisboa. Avenida Professor Egas Moniz, 1649-028, Lisboa, Portugal.

6. Clínica Universitária de Estomatologia, Faculdade de Medicina, Universidade de Lisboa. Avenida Professor Egas Moniz, 1649-028 Lisboa, Portugal.

7. CIDNUR - Centro de Investigação, Inovação e Desenvolvimento em Enfermagem de Lisboa Escola Superior de Enfermagem de Lisboa. Avenida Professor Egas Moniz, 1600-190 Lisboa.

8. CRC-W—Católica Research Centre for Psychological, Family and Social Wellbeing, Universidade Católica Portuguesa, Palma de Cima, 1649-023 Lisboa, Portugal.

9. National Institute of Health Dr. Ricardo Jorge, Av. Padre Cruz, 1600-560 Lisboa, Portugal.

* **Correspondence**: cecilia.elias@gmail.com

Confidential: For Review Only

ABSTRACT

Background: COVID-19 is considered by WHO a pandemic with public health emergency repercussions. Children often develop a mild disease with good prognosis and the recognition of children at risk is essential to successfully manage paediatric COVID-19. Quality epidemiological surveillance data is required to characterize and assess the pandemic.

Methods: Data on all reported pediatric COVID-19 cases, in Portugal, was retrospectively assessed from a fully anonymized dataset provided by the Directorate-General for Health (DGS). Pediatric hospital admission results were obtained from the DGS vaccine recommendations and paediatric intensive care unit (PICU) admission results from the Epicentre.pt group. Reported cases and PICU admissions from March 2020 to February 2021 and hospital admissions between March and December 2020 were analyzed.

Results: 92,051 COVID-19 cases were studied, 50.5% males, average age of 10.1 years, corresponding to 5.4% of children in Portugal. The most common symptoms were cough and fever, whereas gastrointestinal symptoms were infrequent. The most common comorbidity was asthma. A high rate of missing surveillance data was noticed, on presentation of disease and comorbidity variables, which warrants a cautious interpretation of results. Hospital admission was required in 0.93% of cases and PICU on 3.48 per 10,000 cases. PICU admission for Multisystem Inflammatory Syndrome in Children was more frequent in children with no comorbidities and males, severe COVID-19 was rarer and occurred mainly in females and infants. Case fatality rate and mortality rates were low, 1.8 per 100,000 cases and 1.2 per 1,000,000 respectively.

Conclusions: The clinical presentations were predominantly respiratory and neurologic, gastrointestinal symptoms were infrequent. Less than 1% of cases required hospital admission. MIS-C was more frequent in patients with no comorbidities and males. Mortality and case-fatality rates were low. Geographic adapted strategies, and information systems to facilitate surveillance, policy decision making, audit and research are required to improve surveillance data.

Confidential: For Review Only

Keywords: COVID-19, Paediatric, Epidemiology, Hospital Admission, MIS-C, PICU, Health surveillance data

What we know about this subject:

- Quality health surveillance data is essential assess the COVID-19 pandemic.
- Children present a milder course of disease and a lower susceptibility to being severely affected by COVID-19.
- Globally, pediatric COVID-19 case-fatality and mortality rates are low.

What our study adds:

- Portuguese paediatric surveillance data and systems need improvement.
- Paediatric COVID-19 incidence was 5.4% during the first pandemic year.
- Portuguese children and adolescents presented an low mortality and case-fatality rate, 1.8 per 100,000 cases and 1.2 per 1,000,000 respectively.

Confidential: For Review Only

BACKGROUND

Coronavirus disease 2019 (COVID-19), caused by severe acute respiratory syndrome coronavirus 2 (SARS-CoV-2), is considered by the World Health Organization (WHO) a pandemic with public health emergency repercussions.[1],[2] Worldwide, millions of people have been infected with COVID-19 since the end of 2019 among all age groups.[3] Children are also at risk for COVID-19 but have experienced a lower incidence than other age groups.[4],[5] Laboratory-confirmed COVID-19 cases in the United States reveal that only 1.7% of cases were among children and adolescents under 18 years old.[6] Furthermore, several studies showed these age groups have lower susceptibility to being severely affected, often developing a mild form of disease and with a likely good prognosis.[7–9] Studies have shown that children present different symptoms [10], distinct comorbidities, decreased disease severity and fewer disease complications.[10] The characteristics and clinical manifestations of infected children[11],[12] have been considered of major interest.[13] The most frequent symptoms described in children have been fever, cough[14] and ear, nose, and throat complaints. However, other manifestations such as gastrointestinal symptoms have also been identified.[12],[15],[16] A small number of children needed hospital admission, and of these, a minority required ICU admission either due to severe COVID-19 or multisystem inflammatory disease in children (MIS-C). Several risk factors have been associated with these conditions such as extreme age, male sex, comorbidities and non-white background.[17]

As COVID-19 characteristics and clinical course among children can differ from adult patients in numerous ways, specific recommendations for the diagnosis, treatment and vaccination plans in children are being implement worldwide, despite differences across countries.[18],[19],[20],[21] Increasing knowledge of the clinical characteristics in this demographic group may also contribute towards the development and continuous adaptation of preventive strategies that can benefit all communities. Epidemiological surveillance data is essential to attain these objectives. Epidemiological surveillance is a critical part of public health practice [7],[22] where continuous collection, analysis, and interpretation of health data helps identify disease clusters, assess health trends, monitor interventions, identify high risk groups and guide decision making and action.[23] Furthermore, epidemiological data quality is essential and should be monitored to guarantee data results are meaningful.[24]

Confidential: For Review Only

This study aimed to investigate the epidemiological and clinical characteristics of pediatric SARS-CoV-2 infection cases, hospital and ICU admissions, compare these to other countries and to assess the quality of epidemiological surveillance data in Portugal.

MATERIALS AND METHODS

STUDY DESIGN AND DATA

Data on all cases of COVID-19 in children and adolescents under the age of 18, in Portugal, diagnosed between March 2nd, 2020, and February 28th, 2021, were retrospectively assessed. The dataset was fully anonymized and was provided by the Directorate-General for Health (DGS) on 11th of March 2021. The data was collected by the SINAVE (National Epidemiological Surveillance System). The SINAVE is an online system aimed at the electronic notification of mandatory reporting diseases in Portugal. The SINAVE database includes demographic and clinical information collected from all reported patients. Epidemiological Surveillance data in SINAVE is meant to be filled by public health doctors and nurses. However, due to the COVID-19 pandemic increased workload, other professionals from a diverse background have also been recruited to support in surveillance. According to the WHO definition, a confirmed SARS-CoV-2 case was based on a positive polymerase chain reaction test.[25] Hospital admissions with a SARS-CoV-2 primary or secondary diagnosis, from March 2nd to December 31st 2020, were obtained as results from the Pediatric COVID-19 vaccination technical recommendation by DGS.[26] These results include admissions to all hospitals in Portugal with a SARS-CoV-2 diagnosis. The National Health Service Hospitals (SNS), freely cover the paediatric population in the country. No information on whether the SARS-CoV-2 diagnosis was the primary or secondary cause of admission was available. Data on pediatric SARS-CoV-2 PICU admissions, from March 2020 to February 2021, were obtained as anonymized data results on severe COVID-19 [27] and Multisystem inflammatory syndrome in children (MIS-C)[28] from the Epicentre.pt work group. DGS information was analyzed to obtain the number of deaths caused by COVID-19 and these overlapped with the recorded SINAVE case deaths. Data from the National Statistics Institute (INE) was used for mortality and population comparisons. The pediatric mid 2020 population was used by proxy as an estimate population for the period analyzed. Patients and public were not involved in the design or analysis developed in this study.

Confidential: For Review Only

VARIABLES

The SINAVE system includes two variables which indicate the patient's clinical presentation (as symptomatic or asymptomatic) and comorbidities (present, absent). Information on symptoms and comorbidities associated with each case notification should also be filled in through individual variables. For a given symptom or comorbidity, health professionals selected the respective field as yes or no, according to the patient's information. However, any information not filled, for a given symptom or comorbidity, created a missing value in the database. Considering this, a new variable was calculated to describe if a patient was symptomatic or not, using the patient's clinical presentation variable and associated symptoms variables and similarly for the presence of comorbidities. A patient was considered symptomatic either if health professionals classified it as symptomatic through the SINAVE original variable or at least one symptom was reported. In addition, those with any symptom variable filled in but not previously recorded as symptomatic were considered symptomatic patients. Patients' symptomatology status was missing when no symptom variables were filled, and no information regarding the presentation of symptoms in the original variable. A patient was considered having comorbidities either if the presence of comorbidities was selected or at least one comorbidity was reported.

Statistical analysis

Descriptive statistics were used to summarize univariate characteristics. Results were presented as absolute and relative frequencies for qualitative variables, and as means, medians and standard deviations (SD) for quantitative variables. Calculated percentages excluded missing cases unless otherwise stated. Differences between groups were evaluated by Chi-square test with significance level set at 5%. All analysis were conducted using R software version 3.6.3 (R Foundation for Statistical Computing, Vienna, Austria) and IBM SPSS Statistics 28.0.1.1 (14).

RESULTS

GENERAL CHARACTERISTICS

The present study included 92,051 COVID-19 cases in children and adolescents reported from March 2nd, 2020, to February 28th, 2021, in Portugal; 46,453 (50.5%) were males, and 45583 (49.5%) females.

Confidential: For Review Only

An overall reported case incidence of 5.4 per 100 children and adolescents, 5.3% males and 5.5% females. The age profile of patients was distributed between 0 and 17 years old, with the number of cases increasing with age (Table 1). An average age of 10.1 (standard deviation of 5.0) and a median of 11.0 years were observed.

The geographical distribution highlights most cases occurred in 3 urban areas, the Lisbon district (n=22,999, 25%), followed by Porto (n=18,350, 19.9%) and Braga (n=11,091, 12%). The areas with fewer cases were predominantly rural districts (Portalegre and Beja) and autonomous regions of the Azores and Madeira (Figure 1). The children and adolescents with COVID-19 had mostly Portuguese nationality (n = 88,387, 95.3%). Only 4% were from other backgrounds, mainly from Brazil, Angola, Cape Verde, São Tome and Principe and scattered from a wide range of countries.

COVID-19 CASES TIMELINE

During this study, several COVID-19 epidemic waves were observed (Figure 2): 1) between March and April 2020, when 738 cases were reported (0.8% of all cases); 2) between May and July 2020, when 1756 cases (1.9% of all cases) were reported; 3) from September to December 2020, when 39897 cases (43.3% of all cases) were reported; and 4) from January to March 2021, when 46,634 cases (50.7% of all cases) were reported.

CLINICAL PRESENTATION

Information on disease presentation, as symptomatic or asymptomatic, was reported in 47,470 (52%) of cases while there was no information regarding the presence or absence of symptoms in 44,192 cases (48%). From cases with information on clinical presentation, 13,077 (27.5%) were asymptomatic and 34,393 (72.5%) symptomatic. Specific symptoms were reported in 21,077 (44.4%) of cases and a further 13,316 (14.5%) where recorded as symptomatic without reporting symptoms. The asymptomatic presentation of the disease increased with age, peaking between 4 and 10 years of age and slightly decreasing subsequently.

https://mc.manuscriptcentral.com/bmjpo

Confidential: For Review Only

To describe the symptomatic presentations an analysis which exclusively included cases where symptoms were recorded was performed (n = 21,077). The most common symptoms reported were cough (n = 10,205, 48.4%), fever (n = 9,812, 46.6%), rhinorrhea (n = 7,739, 36.7%), and headache (n = 7,143, 33.9%). Gastrointestinal symptoms, such as abdominal pain, nausea and vomiting and diarrhea were reported in 7.1%, 7.8% and 10.2% respectively. Analyzing the distribution of the co-occurrence of clinical COVID-19 symptoms, in each pediatric patient, fever was commonly combined with cough (38.2%), rhinorrhea (29.8%), headache (29%), odynophagia (21%) or myalgia (16.6%). Furthermore, combinations of these symptoms were the most common presentations reported. Fever, cough, and rhinorrhea were the most common presentations for children from 0 to 9 (Table 2). Children aged 0 presented with the highest rates of fever (48%), cough (37%), rhinorrhea (35%) and diarrhea (24%) when compared to the other age groups. From age 10-17 years, headache was the most common reported symptom, followed by fever, cough and rhinorrhea.

COMORBIDITIES

Information on the presence or absence of comorbidities was reported in 19,680 (21.4%) of cases and there was no information regarding the presence or absence of symptoms in 72,371 (78.6%). A total of 1,593 patients presented with comorbidities, of which 1,000 were specified. The three most frequent comorbidities associated with COVID-19 in children and adolescents were: asthma (n = 753, 0.8%), neuromuscular diseases (n = 49) and diabetes (n = 55). Most patients with asthma or neurologic disease were males, whereas most patients with diabetes were females. (Table 3)

MISSING DATA ANALYSIS

Our analysis revealed a high rate of missing values in non-mandatory filling variables (Table 4): disease presentation and comorbidity results were missing in 44,177 (48%) and 72,371 (78.6%) of cases, respectively. Disease presentation presented statistical significance between districts and months; whereas comorbidities exhibited significant differences between sexes, district and months. The two

Confidential: For Review Only

districts with the highest recorded data were Lisbon and Faro. As the pandemic progressed, both these variables presented higher levels of missingness.

HOSPITAL AND PICU ADMISSIONS AND DEATHS

A total of 476 children were admitted to hospital with a COVID-19 ICD-10 code, either as primary or secondary diagnosis. (Table 5) Children aged from 0 – 4 represented 47.9% (n = 228) of hospital admissions, corresponding to 2.24% of all Covid-19 cases in this age group during this time. Overall, 2.8 per 10,000 children in Portugal required hospital admission. No other sociodemographic or clinical variables were available to further characterize this population.

A total of 32 children required PICU admission, corresponding to 0.19 PICU admissions per 10,000 children and adolescents in Portugal. Of these, 28 (87.5%) were admitted due to MIS-C and 4 (12.5%) due to severe COVID-19. MIS-C patients were predominantly male (21, 75%) with ages from 15 months to 17 years age, average of 10.75 and median of 10.5 years. Most cases occurred between January and February 2021. Six patients presented comorbidities. No deaths or major disability were observed. Severe COVID-19 patients were mainly females, under the age of one and all presenting with comorbidities. Two deaths caused by COVID-19 were reported by the DGS (the National Health Authority) during this study and coincided with two deaths recorded in the SINAVE database.

.

Confidential: For Review Only

Discussion

In this study, epidemiological and clinical characteristics of pediatric COVID-19 infections, hospital and PICU admissions in Portugal were described. Health surveillance information analysis revealed an essential and urgent need for improvement.

General Characteristics - Although there is accessible and exhaustive knowledge regarding adult COVID-19 disease[29],[30] pediatric COVID-19 epidemiology and clinical characteristics are significantly less studied and documented in the literature. SARS-CoV-2 infection studies have shown that children are more likely to be asymptomatic than adults [31] and present a less severe disease course.[32] [33] Young children and infants are reported as more vulnerable to infection [34] than older pediatric patients. COVID-19 related fatalities have been reported as scarce in the pediatric population.[6] In this context, we assessed the characteristics and clinical features of COVID-19 in children in Portugal.

We observed an increase of COVID-19 cases with age in both sexes with slightly higher number of cases in males compared to females (50.5% vs. 49.5%). Overall, in the scientific literature, males are most affected, although individual smaller studies have shown either sex as being the most frequent.[9],[33]

Most of these cases were geographically clustered in the high-density urban areas of Lisbon, Porto, and Braga. The association between population density and cumulative infection cases spread was previously observed at a county level in the United States of America and considered an effective predictor of cumulative infection cases.[35] The regions with lower number of cases were mainly rural and low-density populated districts, such as Portalegre, Beja and the autonomous regions of the Azores and Madeira. The nationality of COVID-19 pediatric patients reflects the population's background. The majority were Portuguese and 4% come from diverse backgrounds such as Brazil, Angola, Cape Verde, and São Tome and Principe, countries with historical ties and emigration patterns to Portugal.

Several COVID-19 waves were observed in our study. Following the first wave, in March and April 2020, the first State of Emergency [36], and lockdown were implemented, including school closures, mandatory remote work, limiting mobility, business hours, and social gatherings. Between March 17th

Confidential: For Review Only

and April 2nd, all children who attended the Emergency Department (ED) of a Portuguese hospital with suspected COVID-19 symptoms as cough, fever or rhinorrhoea were tested. Of all 94 cases, one was positive.[37] This can indicate a low incidence of disease at this stage which is in accordance with our results. Studies on antibody seroprevalence of SARS-CoV-2 have shown that only half the children and adolescents had developed disease symptoms.[38]

Clinical Presentation and Comorbidities - The most common symptoms identified (cough, fever, rhinorhoea and headache) have been dominant in meta-analysis describing clinical characteristics in children: Wang et al. reported the presence of fever in 48% and cough 39% of the cases.[14] Li et al. in 7004 pediatric cases reported fever 47% and cough 42% as the most prevalent clinical symptoms [39]. The presence of gastrointestinal symptoms has been identified as major symptoms occurring in the pediatric population.[40],[41],[42] In our analysis, diarrhea, abdominal pain, nausea and vomiting were less frequent than other symptoms like cough, fever and rhinorrhea as has been reported in other studies.[4],[33] Interestingly, the most common symptoms per age groups can be organized in 3 sets: 1) from ages zero to four years: fever, cough and rhinorrhea; 2) from ages five to nine years: fever, cough, rhinorrhea and headache; and 3) from 10 to 17 years: the previous 4 symptoms, odynophagia and myalgia. This result can reflect simultaneously the inability of young children to express their complaints and an increase in the variety of symptoms and presentations as age increases.

Asthma was the most common underlying condition and has been the most reported comorbidity among several pediatric cases as well.[6],[5],[33]. Verma et al. analysed SARS-CoV-2 infections in children in four hospitals in the New York City area and found those with asthma were more likely to need respiratory support during hospitalization.[43] In a systematic review, including 5686 children aged under 18 years with SARS-CoV-2 infection, the most frequent comorbidity was cardiac disease; and most children who required ventilation had underlying comorbidities.[4] A study of ED and admitted pediatric COVID-19 patients at a Level 3 Lisbon Hospital, one of two reference hospitals for COVID-19 pediatric patients at this point in the pandemic, showed 10% had risk factors for disease, 20% were asymptomatic, 43% presented with fever and 42% with respiratory symptoms.[44] Though the referred study is likely representing the most symptomatic COVID-19 cases seeking medical help, whereas our study focuses on all pediatric COVID-19 cases, chronic respiratory disease was also the most common

Confidential: For Review Only

comorbidity. Only a small proportion of children with COVID-19 had registered comorbidities (n = 1593, 2%) which is lower than reported in other studies[33] and of what the authors expected.

Missing Surveillance Data Analysis - The quality of surveillance data was assessed trough a missingness analysis, given the abnormally high number of missing clinical presentations (48%) and comorbidities (78.6%). Data results progressively worsened with the pandemic and particularly when case numbers sharply increased. These results can be consequential of massive work overload observed in the public health units combined with short staff. Furthermore, with increasing case counts, non-public health professionals were recruited to support the units. However, significant asymmetries were also evident according to district, with substantial fluctuations in recording rates across the country. Notwithstanding, good practice and the SINAVE guidelines warrant all fields to be answered and to achieve this goal, measures and geographically adapted strategies, such as increased training, resources, and continued auditing, need to be implemented to improve surveillance data. The SINAVE system collects symptoms and comorbidities in a non-mandatory and non-intelligent way, not tailored to previous answers. This system also needs to be improved and updated from a user perspective, so to fast-track user experience and enhance clinical record keeping.

Hospital and PICU admissions - In 2020, a total of 476 children were admitted to the hospital from 51,153 cases reported, corresponding to 1 in 108 cases requiring hospitalization, a rate of 0.93% of cases nationwide and a paediatric hospitalization rate of 2.8 per 10,000 children and adolescents. A lower case hospitalization result than reported in neighbouring Navarra, Spain (3.6%) during the first wave[45], in the United States (5.3%)[46] and in Scotland, where 1.03% of cases in children aged 5- to 17-year-olds required hospitalization[47]. These results probably reflect a higher testing rate of asymptomatic or mildly ill children in Portugal.

Thirty-two cases required PICU admission, corresponding to a rate of admission of 3.48 per 10,000 cases. Four cases (females under the age of one year and presenting comorbidities; 12.5%) were admitted with severe COVID-19 (non-MIS-C) and twenty-eight (87.5%) with MIS-C, implying that in our population, severe COVID-19 in children is less frequent than MIS-C. Patients with MIS-C were predominantly male and the majority (75%) had no comorbidities, which is similar to previously

Confidential: For Review Only

published results, where most MIS-C patients were healthy, and no correlation was observed between comorbidity score and MIS-C occurrence.[27]

Deaths - During this period, the Directorate-General of Health confirmed two paediatric deaths caused by COVID-19, corresponding to a case-fatality rate of approximately 1.8 per 100,000 cases. Furthermore, analysing specifically total paediatric deaths in Portugal in 2020 (n = 387) and comparing to the paediatric COVID-19 deaths, COVID-19 was the main cause of death in 0.52%. The mortality rate due to COVID-19 was 1.2 per million children and adolescents. These results find parallel to similar studies, highlighting the low mortality and case-fatality rates. Despite the extremely small number of deaths, it is reasonable to compare these results with other countries. Bhopal et al reported an overall mortality rate due to COVID-19 in children of 1.7 per 10.000, until February 2021 when comparing seven countries. Smith et al.[48] reported a mortality rate due to COVID-19 of 2 per million and fatality rate of 5 per 100,000 in England, between March 2020 and February 2021, a result 1.7 and 2.5 times higher than the one we report, respectively.[43] Spanish mortality results were published by Tagarro et al.[49] and reported 0.21 per 100 000 in children aged 0 to 9 years and 0.34 per 100 000 in children aged 10 to 19 years. These results may reflect differences in shielding of vulnerable population or lockdown policies implemented in these countries, but further studies are required to analyse these effects.

The major strengths of our study are the combined analysis of a large database of COVID-19 cases, hospital and PICU admissions and deaths. These results present a description of paediatric COVID-19 in Portugal and allow comparison with other countries. A missingness analysis was performed and highlights strategic improvements required to epidemiological surveillance data.

The authors found the elevated level of missingness in surveillance data unusual. The analysis of real-world data has important challenges as previously acknowledged[50], however other studies using national data have reported similar difficulties[51],[52]. Despite the authors best effort in this analysis, less than optimal real-world data has imposed important limitations to the study and hindered some of its quality, particularly relating to missing values in some of the analyzed variables. The authors are developing further studies and proposing solutions to help prevent these issues.[51],[52] As stated, the major limitations of this analysis are data-related, besides the level of missingness observed, the

https://mc.manuscriptcentral.com/bmjpo

Confidential: For Review Only

possible underreporting of cases and its characteristics, such as symptoms and comorbidities, the limited access to hospital admission data and deaths and the impracticality of establishing a chronological order of events. Furthermore, the estimated population used for comparison was obtain by-proxy with the reported paediatric for 2020.

Conclusions

The clinical manifestations of disease reported in our analysis were predominantly respiratory and neurologic, while gastrointestinal symptoms were infrequent. Less than 1% of patients required hospital admission. MIS-C was more frequent in patients with no comorbidities and males, severe COVID-19 was rarer and occurred mainly in children under 1 year of age and females, all with comorbidities. Case fatality rates and mortality rates were low when comparing with other countries. This study highlights the need to improve epidemiological surveillance data in Portugal, with geographic adapted strategies, and information systems to facilitate adequate epidemiological surveillance, policy decision making, audit and research.

Confidential: For Review Only

ACKNOWLEDGEMENTS

The authors would like to thank Dr Cristina Camilo and the EPICENTRE.PT research group.

FUNDING

Funds from Fundação para a Ciência e a Tecnologia to the Instituto de Saúde Ambiental, Faculdade de Medicina, Universidade de Lisboa (UIDB/04295/2020).

Confidential: For Review Only

CONFLICT OF INTERESTS

The authors declare no conflict of interest.

CONTRIBUTORSHIP STATEMENT

Cecilia Elias planned the study and was the guarantor. She developed the methodology and was involved in obtaining the data, data analysis, writing the manuscript, creating tables/figures and reviewing the text. Rodrigo Feteira-Santos was involved in obtaining data, writing the manuscript, creating tables and figures and reviewing the text. Catarina Camarinho was involved in planning the study, creating tables and figures, writing and reviewing the manuscript and maintaining references. Miguel de Araújo Nobre was involved in writing and reviewing the manuscript. Andreia Costa was involved in writing and reviewing the manuscript. Leonor Bacelar was involved in writing and reviewing the manuscript. Cristina Furtado was involved in writing and reviewing the manuscript. Paulo Nogueira was involved in planning the study, data analysis and writing and reviewing the manuscript.

References

[1] Cucinotta D, Vanelli M. WHO Declares COVID-19 a Pandemic. Acta Biomed 2020;91:157–60. https://doi.org/10.23750/abm.v91i1.9397.

[2] WHO/Europe | Coronavirus disease (COVID-19) outbreak - WHO announces COVID-19 outbreak a pandemic.

[3] Wu Z, McGoogan JM. Characteristics of and Important Lessons From the Coronavirus Disease 2019 (COVID-19) Outbreak in China: Summary of a Report of 72 314 Cases From the Chinese Center for Disease Control and Prevention. JAMA 2020;323:1239–42. https://doi.org/10.1001/jama.2020.2648.

[4] Williams N, Radia T, Harman K, Agrawal P, Cook J, Gupta A. COVID-19 Severe acute respiratory syndrome coronavirus 2 (SARS-CoV-2) infection in children and adolescents: a systematic review of critically unwell children and the association with underlying comorbidities. Eur J Pediatr 2021;180:689–97. https://doi.org/10.1007/s00431-020-03801-6.

[5] Stokes EK, Zambrano LD, Anderson KN, Marder EP, Raz KM. Coronavirus Disease 2019 Case Surveillance — United States, January 22–May 30, 2020. MMWR Morb Mortal Wkly Rep 2020 2020;69:759–65.

[6] Bialek S, Gierke R, Hughes M, McNamara LA, Pilishvili T, Skoff T. Coronavirus disease 2019 in children: Current status - Morbidity and Mortality Weekly Report 2020;69:422–6.

[7] Dong Y, Mo X, Hu Y, Qi X, Jiang F, Jiang Z, et al. Epidemiology of COVID-19 Among Children in China. Pediatrics 2020;145:20200702. https://doi.org/10.1542/PEDS.2020-0702.

[8] Mantovani A, Rinaldi E, Zusi C, Beatrice G, Saccomani MD, Dalbeni A. Coronavirus disease 2019 (COVID-19) in children and/or adolescents: a meta-analysis. Pediatr Res 2021;89:733–7. https://doi.org/10.1038/s41390-020-1015-2.

[9] Götzinger F, Santiago-García B, Noguera-Julián A, Lanaspa M, Lancella L, Calò Carducci FI, et al. COVID-19 in children and adolescents in Europe: a multinational, multicentre cohort study. Lancet Child Adolesc Heal 2020;4:653–61. https://doi.org/10.1016/S2352-4642(20)30177-2.

[10] Zare-Zardini H, Soltaninejad H, Ferdosian F, Hamidieh AA, Memarpoor-Yazdi M. Coronavirus Disease 2019 (COVID-19) in Children: Prevalence, Diagnosis, Clinical Symptoms, and Treatment. Int J Gen Med 2020;13:477–82. https://doi.org/10.2147/IJGM.S262098.

[11] Guo CX, He L, Yin JY, Meng XG, Tan W, Yang GP, et al. Epidemiological and clinical features of pediatric COVID-19. BMC Med 2020;18:1–7. https://doi.org/10.1186/s12916-020-01719-2.

[12] Zimmermann P, Curtis N. Coronavirus infections in children including COVID-19: An overview of the epidemiology, clinical features, diagnosis, treatment and prevention options in children. Pediatr Infect Dis J 2020;39:355–68. https://doi.org/10.1097/INF.0000000000002660.

[13] Davies NG, Klepac P, Liu Y, Prem K, Jit M, Pearson CAB, et al. Age-dependent effects in the transmission and control of COVID-19 epidemics. Nat Med 2020;26:1205–11. https://doi.org/10.1038/s41591-020-0962-9.

Confidential: For Review Only

[14] Wang Z, Zhou Q, Wang C, Shi Q, Lu S, Ma Y, et al. Clinical characteristics of children with COVID-19: a rapid review and meta-analysis. Ann Transl Med 2020;8:620. https://doi.org/10.21037/atm-20-3302.

[15] Mayor S. Covid-19: UK studies find gastrointestinal symptoms are common in children. BMJ 2020;370. https://doi.org/10.1136/bmj.m3484.

[16] Giacomet V, Barcellini L, Stracuzzi M, Longoni E, Folgori L, Leone A, et al. Gastrointestinal symptoms in severe covid-19 children. Pediatr Infect Dis J 2020;39:E317–20. https://doi.org/10.1097/INF.0000000000002843.

[17] Ward JL, Harwood R, Smith C, Kenny S, Clark M, Davis PJ, et al. Risk factors for PICU admission and death among children and young people hospitalized with COVID-19 and PIMS-TS in England during the first pandemic year. Nat Med 2022;28:193–200. https://doi.org/10.1038/s41591-021-01627-9.

[18] Maltezou HC, Vorou R, Papadima K, Kossyvakis A, Spanakis N, Gioula G, et al. Transmission dynamics of SARS-CoV-2 within families with children in Greece: A study of 23 clusters. J Med Virol 2021;93:1414–20. https://doi.org/10.1002/jmv.26394.

[19] Han Y-N, Feng Z-W, Sun L-N, Ren X-X, Wang H, Xue Y-M, et al. A comparative-descriptive analysis of clinical characteristics in 2019-coronavirus-infected children and adults. J Med Virol 2020;92:1596–602. https://doi.org/10.1002/jmv.25835.

[20] Bi Q, Wu Y, Mei S, Ye C, Zou X, Zhang Z, et al. Epidemiology and transmission of COVID-19 in 391 cases and 1286 of their close contacts in Shenzhen, China: a retrospective cohort study. Lancet Infect Dis 2020;20:911–9. https://doi.org/10.1016/S1473-3099(20)30287-5.

[21] Kawasuji H, Takegoshi Y, Kaneda M, Ueno A, Miyajima Y, Kawago K, et al. Transmissibility of COVID-19 depends on the viral load around onset in adult and symptomatic patients. PLoS One 2020;15:e0243597. https://doi.org/10.1371/journal.pone.0243597.

[22] Ibrahim NKR, Al Bar HM. Surveillance of childhood vaccine-preventable diseases at health facilities in Jeddah, Saudi Arabia. East Mediterr Health J 2009;15:532–43.

[23] Choi BCK. The Past, Present, and Future of Public Health Surveillance. Scientifica (Cairo) 2012;2012:875253. https://doi.org/10.6064/2012/875253.

[24] European Centre for Disease Prevention and Control. Data quality monitoring and surveillance system evaluation – A handbook of methods and applications. Stockholm: ECDC; 2014.

[25] World Health Organization. Laboratory testing for 2019 novel coronavirus (2019-nCoV) in suspected human cases. WHO - Interim Guid 2020;2019:1–7.

[26] Direção Geral da Saúde (DGS). Comissão Técnica de Vacinação contra a COVID-191 Vacinação contra a COVID-19 em crianças com 5 a 11 anos

[27] Preston LE, Chevinsky JR, Kompaniyets L, Lavery AM, Kimball A, Boehmer TK, et al. Characteristics and Disease Severity of US Children and Adolescents Diagnosed With COVID-19. JAMA Netw Open 2021;4:e215298–e215298. https://doi.org/10.1001/jamanetworkopen.2021.5298.

https://mc.manuscriptcentral.com/bmjpo

Confidential: For Review Only

[28] World Health Organization (WHO). Living guidance for clinical management of COVID-19: Living guidance, 23 November 2021.

[29] Lechien JR, Chiesa-Estomba CM, Place S, Van Laethem Y, Cabaraux P, Mat Q, et al. Clinical and epidemiological characteristics of 1420 European patients with mild-to-moderate coronavirus disease 2019. J Intern Med 2020;288:335–44. https://doi.org/https://doi.org/10.1111/joim.13089.

[30] Zhou F, Yu T, Du R, Fan G, Liu Y, Liu Z, et al. Clinical course and risk factors for mortality of adult inpatients with COVID-19 in Wuhan, China: a retrospective cohort study. Lancet 2020. https://doi.org/10.1016/S0140-6736(20)30566-3.

[31] Adeyinka A, Bailey K, Pierre L, Kondamudi N. COVID 19 infection: Pediatric perspectives. J Am Coll Emerg Physicians Open 2021;2:1–11. https://doi.org/10.1002/emp2.12375.

[32] Dong Y, Dong Y, Mo X, Hu Y, Qi X, Jiang F, et al. Epidemiology of COVID-19 among children in China. Pediatrics 2020;145. https://doi.org/10.1542/peds.2020-0702.

[33] Patel NA. Pediatric COVID-19: Systematic review of the literature. Am J Otolaryngol - Head Neck Med Surg 2020;41:102573. https://doi.org/10.1016/j.amjoto.2020.102573.

[34] Dong Y, Mo X, Hu Y, Qi X, Jiang F, Jiang Z, et al. Epidemiology of COVID-19 Among Children in China. Pediatrics 2020;145. https://doi.org/10.1542/peds.2020-0702.

[35] Wong DWS, Li Y. Spreading of COVID-19: Density matters. PLoS One 2020;15:e0242398.

[36] Diário da República 1.a série. Decreto do Presidente da República n.o 14-A/2020 de 18 de março. 2020.

[37] Costa A, Almeida H, Moniz M, Alves C. COVID-19 screening in a Portuguese pediatric population. Enferm Infecc Microbiol Clin 2020:9–12. https://doi.org/10.1016/j.eimc.2020.09.004.

[38] Felsenstein S, Hedrich CM. SARS-CoV-2 infections in children and young people. Clin Immunol 2020;220:108588. https://doi.org/https://doi.org/10.1016/j.clim.2020.108588.

[39] Li B, Zhang S, Zhang R, Chen X, Wang Y, Zhu C. Epidemiological and Clinical Characteristics of COVID-19 in Children: A Systematic Review and Meta-Analysis. Front Pediatr 2020;8:591132. https://doi.org/10.3389/fped.2020.591132.

[40] Waterfield T, Watson C, Moore R, Ferris K, Tonry C, Watt A, et al. Seroprevalence of SARS-CoV-2 antibodies in children: a prospective multicentre cohort study. Arch Dis Child 2021;106:680–6. https://doi.org/10.1136/archdischild-2020-320558.

[41] Akobeng AK, Grafton-Clarke C, Abdelgadir I, Twum-Barimah E, Gordon M. Gastrointestinal manifestations of COVID-19 in children: a systematic review and meta-analysis. Frontline Gastroenterol 2021;12:332–7. https://doi.org/10.1136/flgastro-2020-101529.

[42] Al-Beltagi M, Saeed NK, Bediwy AS, El-Sawaf Y. Paediatric gastrointestinal disorders in SARS-CoV-2 infection: Epidemiological and clinical implications. World J Gastroenterol 2021;27:1716–27. https://doi.org/10.3748/wjg.v27.i16.1716.

[43] Verma S, Lumba R, Dapul HM, Gold-von Simson G, Phoon CK, Lighter JL, et al. Characteristics of Hospitalized Children With SARS-CoV-2 in the New York City Metropolitan Area. Hosp Pediatr 2021;11:71–8. https://doi.org/10.1542/hpeds.2020-001917.

Confidential: For Review Only

[44] Picão de Carvalho C, Castro C, Sampaio Graça I, Lorenzo C, Barbosa Rodrigues A, Inácio R, et al. Série de Casos de 103 Crianças com Infeção por SARS-CoV-2 em Portugal. Acta Med Port 2020;33:795. https://doi.org/10.20344/amp.14537.

[45] Moreno-Galarraga L, Urretavizcaya-Martínez M, Alegría Echauri J, García Howard M, Ruperez García E, Aguilera-Albesa S, et al. SARS-CoV-2 infection in children requiring hospitalization: the experience of Navarra, Spain. World J Pediatr 2020;16:614–22. https://doi.org/10.1007/s12519-020-00393-x.

[46] Kim TY, Kim EC, Agudelo AZ, Friedman L. COVID-19 hospitalization rate in children across a private hospital network in the United States: COVID-19 hospitalization rate in children. Arch Pediatr 2021;28:530–2. https://doi.org/10.1016/j.arcped.2021.07.004.

[47] Shi T, Pan J, Katikireddi SV, McCowan C, Kerr S, Agrawal U, et al. Risk of COVID-19 hospital admission among children aged 5–17 years with asthma in Scotland: a national incident cohort study. Lancet Respir Med 2022. https://doi.org/10.1016/S2213-2600(21)00491-4.

[48] Smith C, Odd D, Harwood R, Ward J, Linney M, Clark M, et al. Deaths in children and young people in England after SARS-CoV-2 infection during the first pandemic year. Nat Med 2022;28:185–92. https://doi.org/10.1038/s41591-021-01578-1.

[49] Tagarro A, García-Salido A, Martínez E-V, Vega-Piris L, Mellado MJ. Low COVID-19 mortality in Spanish children. Lancet Child Adolesc Heal 2021;5:e24–5. https://doi.org/10.1016/S2352-4642(21)00125-5.

[50] Liu M, Qi Y, Wang W, Sun X. Toward a better understanding about real-world evidence. Eur J Hosp Pharm Sci Pract 2022;29:8–11. https://doi.org/10.1136/ejhpharm-2021-003081.

[51] Nogueira PJ, de Araújo Nobre M, Costa A, Ribeiro RM, Furtado C, Bacelar Nicolau L, et al. The Role of Health Preconditions on COVID-19 Deaths in Portugal: Evidence from Surveillance Data of the First 20293 Infection Cases. J Clin Med 2020;9. https://doi.org/10.3390/jcm9082368.

[52] Nogueira PJ, de Araújo Nobre M, Costa A, Ribeiro RM, Furtado C, Bacelar Nicolau L, et al. Reply to Comment on Nogueira, P.J., et al. "The Role of Health Preconditions on COVID-19 Deaths in Portugal: Evidence from Surveillance Data of the First 20293 Infection Cases". J. Clin. Med. 2020, 9, 2368. J Clin Med 2020;9. https://doi.org/10.3390/jcm9113449.

Confidential: For Review Only

TABLE 1. AGE AND SEX OF PEDIATRIC COVID-19 INFECTED CASES, FROM MARCH 2020 TO FEBRUARY 2021 IN PORTUGAL.

		Total n (%)***	Female n (%)**	Male n (%)**	Reported cases per 100 children (%)
N (%)		92051	45583 (49.5%)	46453 (50.5%)	5.4%
Age	0	1719 (1.9%)	826 (1.8%)	884 (1.9%)	2.0%
	1	3725 (4.0%)	1784 (3.9%)	1940 (4.2%)	4.3%
	2	3692 (4.0%)	1860 (4.1%)	1832 (3.9%)	4.2%
	3	3332 (3.6%)	1575 (3.5%)	1757 (3.8%)	3.8%
	4	3709 (4.0%)	1840 (4.0%)	1869 (4.0%)	4.2%
	Subtotal 1-4 y	14458 (15.7%)	7059 (15.5%)	7398 (15.9%)	4.1%
	5	4023 (4.4%)	1988 (4.4%)	2035 (4.4%)	4.6%
	6	4299 (4.7%)	2128 (4.7%)	2171 (4.7%)	5.1%
	7	4592 (5.0%)	2232 (4.9%)	2360 (5.1%)	5.4%
	8	5058 (5.5%)	2490 (5.5%)	2568 (5.5%)	5.5%
	9	5548 (6.0%)	2709 (5.9%)	2839 (6.1%)	5.7%
	Subtotal 5-9 y	23520 (25.6%)	11547 (25.3%)	11973 (25.8%)	5.3%
	10	5705 (6.2%)	2872 (6.3%)	2833 (6.1%)	5.6%
	11	5726 (6.2%)	2838 (6.2%)	2888 (6.2%)	5.9%
	12	5865 (6.4%)	2928 (6.4%)	2937 (6.3%)	5.8%
	13	6080 (6.6%)	2942 (6.5%)	3137 (6.8%)	6.1%
	14	6449 (7.0%)	3235 (7.1%)	3213 (6.9%)	6.3%
	Subtotal 10-14 y	29825 (32.4%)	14815 (32.5%)	15008 (32.3%)	5,9%
	15	6919 (7.5%)	3402 (7.5%)	3514 (7.6%)	6.5%
	16	7635 (8.3%)	3890 (8.5%)	3744 (8.1%)	7.3%
	17	7975 (8.7%)	4041 (8.9%)	3932 (8.5%)	7.4%
	Subtotal 15-17 y	22529 (24.5%)	11333 (24.9%)	11190 (24.1%)	7.1%

Footnotes: 15 (0.02%) missing values were observed for sex, *** global percentage; **percentage within sex level.

Confidential: For Review Only

TABLE 2. SYMPTOMS ASSOCIATED WITH COVID-19 PER AGE GROUP FROM MARCH 2020 TO FEBRUARY 2021 IN PORTUGAL.

	Age Groups (years)					
	0<br>n (%)	1 - 4<br>n (%)	5 - 9<br>n (%)	10 - 14<br>n (%)	15 - 17<br>n (%)	Total<br>n (%)
Total	1075	8316	11733	14759	11587	47470
Symptoms						
Fever	513 (47.7%)	2846 (34.2%)	2096 (17.9%)	2317 (15.7%)	1965 (17.0%)	9737 (20.5%)
Cough	396 (36.8%)	2397 (28.8%)	1920 (16.4%)	2632 (17.8%)	2771 (23.9%)	10116 (21.3%)
Dyspnea	34 (3.2%)	192 (2.3%)	136 (1.2%)	180 (1.2%)	258 (2.2%)	800 (1.7%)
Rhinorrhoea	376 (35.0%)	2047 (24.6%)	1465 (12.5%)	1927 (13.1%)	1818 (15.7%)	7633 (16.1%)
Odynophagia	13 (1.2%)	364 (4.4%)	1003 (8.5%)	1747 (11.8%)	1831 (15.8%)	4958 (10.4%)
Headache	1 (0.1%)	149 (1.8%)	1351 (11.5%)	2723 (18.4%)	2841 (24.5%)	7065 (14.9%)
Abdominal pain	8 (0.7%)	199 (2.4%)	553 (4.7%)	449 (3.0%)	252 (2.2%)	1461 (3.1%)
Chest pain	0 (0.0%)	11 (0.1%)	59 (0.5%)	148 (1.0%)	214 (1.8%)	432 (0.9%)
Arthralgia	0 (0.0%)	9 (0.1%)	26 (0.2%)	64 (0.4%)	61 (0.5%)	160 (0.3%)
Myalgia	1 (0.1%)	84 (1.0%)	436 (3.7%)	1300 (8.8%)	1731 (14.9%)	3552 (7.5%)
Nausea and vomiting	38 (3.5%)	362 (4.4%)	403 (3.4%)	475 (3.2%)	344 (3.0%)	1622 (3.4%)
Diarrhea	107 (10.0%)	616 (7.4%)	443 (3.8%)	523 (3.5%)	464 (4.0%)	2153 (4.5%)
Convulsions	1 (0.1%)	17 (0.2%)	5 (0.0%)	10 (0.1%)	5(0.0%)	38 (0.1%)
Irritability	19 (1.8%)	43 (0.5%)	9 (0.1%)	9 (0.1%)	5 (0.0%)	85 (0.2%)
General weakness	16 (1.5%)	148 (1.8%)	221 (1.9%)	533 (3.6%)	724 (6.2%)	1642 (3.5%)
Tachycardia	2 (0.2%)	20 (0.2%)	15 (0.1%)	16 (0.1%)	12 (0.1%)	65 (0.1%)

Footnotes: Percentages of patients presenting a specific symptom from patients presenting with the column symptom.

view Only

TABLE 3. COMORBIDITIES OF THE PEDIATRIC COVID-19 CASES, FROM MARCH 2020 TO FEBURARY 2021, IN PORTUGAL.

		Total<br>n (%) ***	Female<br>n (%) **	Male<br>n (%) **
Total cases with comorbidities*		1593	691	902
Total cases with known comorbidities		1000	427	573
Number of comorbidities	0	18087 (94.9%)	9013 (95.5%)	9074 (94.1%)
	1	943 (4.9%)	401 (4.2%)	542 (6.0%)
	2	47 (0.2%)	20 (0.2%)	27 (0.3%)
	3+	10 (0.1%)	6 (0.1%)	4 (0.0%)
Total		19087	9440	9647
Comorbidities				
	Asthma	753 (47.3%)	316 (45.7%)	437 (48.4%)
	Chronic neurologic / neuromuscular disease	49 (3.1%)	24 (3.5%)	25 (2.8%)
	Diabetes	55 (3.5%)	29 (4.2%)	26 (2.9%)
	Chronic haematological disease	40 (2.1%)	17 (2.5%)	23 (2.5%)
	Chronic neurologic deficit	34 (2.1%)	15 (2.2%)	19 (2.0%)
	Cancer	36 (2.3%)	13 (1.9%)	23 (2.5%)
	Chronic pulmonary disease	42 (2.6%)	17 (2.5%)	25 (2.8%)
	Chronic renal disease	28 (1.8%)	15 (2.2%)	13 (1.4%)
	HIV and other immunodeficiencies	25 (1.6%)	12 (1.7%)	13 (1.4%)
	Liver disease	8 (0.5%)	2 (0.3%)	6 (0.6%)

Footnotes: Results of each morbidity were presented considering the sample size of cases with any reported morbidity (n=1,593). *Total cases with comorbidities, of which 1000 are specified. ***Global percentage; **Percentage within sex level.

Only

TABLE 4. PAEDIATRIC COVID-19 MISSING DATA ANALYSIS FOR CASES REPORTED BETWEEN MARCH 2020 AND FEBRUARY 2021, IN PORTUGAL.

	Recorded	%	Missing	%	p	Recorded	%	Missing	%	p
Sex	92036	100%	15	0.0						
District	89650	97.4%	2399	2.6%						
Disease Presentat	47859	52.0%	44177	48.0%						
Comorbidities	19680	21.4%	72371	78.6%						
	**Disease Presentation**					**Comorbidities**				
**Sex**										
Female	23709	52%	21874	48%	0.940	9704	21.3%	35879	78.7%	<0.001
Male	24150	52%	22303	48%		9976	21.4%	36477	78.6%	
**District**										
Aveiro	2451	37.3%	4120	62.7%	<0.001	1084	16.5%	5488	83.5%	<0.001
Beja	295	36.3%	517	63.7%		152	18.7%	660	81.3%	
Braga	5784	52.2%	5307	47.8%		2287	20.6%	8804	79.4%	
Braganca	204	29.0%	499	71.0%		77	11.0%	626	89.0%	
Castelo Branco	556	52.9%	496	47.1%		265	25.1%	787	74.9%	
Coimbra	1088	38.8%	1714	61.2%		573	20.4%	2229	79.6%	
Evora	433	42.6%	584	42.6%		85	8.4%	932	91.6%	
Faro	1826	71.3%	734	28.7%		802	31.3%	1758	68.7%	
Guarda	224	19.5%	926	80.5%		121	10.5%	1029	89.5%	
Leiria	1057	42.1%	1455	57.9%		487	19.4%	2025	80.6%	
Lisboa	16161	70.3%	6838	29.7%		6955	30.2%	16044	69.8%	
Portalegre	268	41.9%	371	58.1%		67	10.5%	572	89.5%	
Porto	7801	42.5%	10549	57.5%		2887	15.7%	15463	84.3%	
Santarem	1196	37.2%	2023	62.8%		469	14.6%	2750	85.4%	
Setubal	4937	62.2%	2950	37.4%		1880	23.8%	6007	76.2%	
Viana do Castelo	777	43.3%	1019	56.7%		309	17.2%	1487	82.8%	
Vila Real	734	50.3%	724	49.7%		311	21.3%	1147	78.7%	
Viseu	1245	41.0%	1788	59%		472	15.6%	2561	84.4%	
**Month**										
March	150	88.2%	20	11.8%	<0.001	117	68.8%	53	31.2%	<0.001
April	470	82.9%	97	17.1%		332	58.6%	235	41.4%	
May	427	91.8%	38	8.2%		297	63.9%	168	36.1%	
June	690	91.6%	63	8.4%		438	58.2%	315	41.8%	
July	774	93.4%	55	6.6%		437	52.7%	392	47.3%	
August	536	92.9%	41	7.1%		298	51.6%	279	48.4%	
September	1440	81.5%	327	18.5%		728	41.2%	1039	58.8%	
October	4205	65.0%	2266	35.0%		1920	29.7%	4551	70.3%	
November	10611	62.8%	9503	47.2%		4672	23.2%	15442	76.8%	
December	7691	53.2%	6766	46.8%		3143	21.7%	11314	78.3%	
January	16893	45.4%	20343	54.6%		6008	16.1%	31228	83.9%	
February	3972	0.46	4673	54.1%		1290	14.9%	7355	85.1%	

Confidential: For Review Only

TABLE 5. PEDIATRIC HOSPITAL FROM MARCH 2020 TO DECEMBER 2020 AND PICU ADMISISONS FROM MARCH 2020 TO FEBRUARY 2021 IN PORTUGAL

Hospital admissions from March to December 2020

Age Group	Hospital admissions n (%)	Cases per age group	Hospital admissions per 100 reported cases	Hospital admissions per 10.000 children
0 - 4	228 (47.9%)	10161	2.24	5.2
5-11	112 (23.5%)	18358	0.61	1.7
12-17	136 (28.6%)	22674	0.60	2.2
Total	476 (100%)	51193	0.93	2.8

PICU admissions from March 2020 to February 2021

	Severe COVID-19	MIS-C	Total ICU admissions
PICU admissions (%)	4 (12.5%)	28 (87.5%)	32 (100%)
PICU admissions per 10.000 reported cases	0.43	3.04	3.48
PICU admissions per 10.000 children	0.02	0.16	0.19
Female (%)	3 (75%)	7 (25%)	10 (31.2%)
Male (%)	1 (25%)	21 (75%)	22 (68.8%)
Min age (months)	3	15	
Max age (years)	17	17	
Median (years)	0	10.5	
Comorbidities present (%)	4 (100%)	6 (21.4%)	10 (31.2%)

ew Only

Confidential: For Review Only

Confidential: For Review Only

FIGURE 1. MAP OF COVID-19 PEDIATRIC CASES IN PORTUGAL, BY DISTRICT IN ABSOLUTE VALUE.

Confidential: For Review Only

FIGURE 2. PEDIATRIC COVID-19 CASES PER WEEK

Footnote: 1. First COVID-19 patient diagnosed in Portugal; 2. First national state of emergency; 3. Lockdown easing; 4. Preschool and primary school reopening; 5. End of the school year; 6. Start of the school year; 7. State of emergency with restrictions; 8. Christmas with minimal gathering restrictions; 9. Reintroduction of mandatory remote working, travel restrictions and school closures; 10. Highest daily death toll due to COVID-19 during the pandemic.

Confidential: For Review Only

Confidential: For Review Only

SUPPLEMENTARY TABLE 1. PEDIATRIC COVID-19 CASES COUNTRY OF ORIGIN, EXCLUDING PORTUGAL

Country	n(%)
Brasil	2170 (59.2%)
Angola	331 (9.0%)
Cape Verde	205 (5.6%)
Saint Tome and Principe	121 (3.3%)
Guine Bissau	86 (2.3%)
Nepal	75 (2.0%)
Ukraine	72 (2.0%)
Italy	66 (1.8%)
France	62 (1.7%)
Venezuela	49 (1.3%)
Spain	47 (1.3%)
United Kingdom	45 (1.2%)
Romenia	45 (1.2%)
India	33 (0.9%)
Bangladesh	27 (0.7%)
Mozambique	23 (0.6%)
Netherlands	19 (0.5%)
Republic of Moldava	17 (0.5%)
Germany	16 (0.4%)
Paquistan	16 (0.4%)
Belgium	14 (0.4%)
Bulgaria	14 (0.4%)
Colombia	12 (0.3%)
Cuba	9 (0.2%)
Ireland	9 (0.2%)
China	7 (0.2%)
United States of America	7 (0.2%)
Other	67 (1.8%)

Review Only

SUPPLEMENTARY TABLE 2. SYMPTOMATIC AND ASYMPTOMATIC COVID-19 CASES PER AGE

Years	Asymptomatic n (%)	Symptomatic n (%)	Total	Total reported cases	Missing information n (%)
0	162 (15.1%)	913 (84.9%)	1075	1719	644 (37.5%)
1	387 (15.9%)	2040 (84.1%)	2427	3725	1298 (34.8%)
2	485 (22.6%)	1658 (77.4%)	2143	3692	1549 (42%)
3	527 (28.2%)	1342 (71.8%)	1869	3332	1463 (43.9%)
4	640 (34.1%)	1237 (65.9%)	1877	3709	1832 (49.4%)
5	704 (34.7%)	1325 (65.3%)	2029	4023	1994 (49.6%)
6	769 (36%)	1367 (64%)	2136	4299	2163 (50.3%)
7	825 (36.4%)	1442 (63.6%)	2267	4592	2325 (50.6%)
8	871 (34.7%)	1640 (65.3%)	2511	5058	2547 (50.4%)
9	966 (34.6%)	1824 (65.4%)	2790	5548	2758 (49.7%)
10	918 (33%)	1868 (67%)	2786	5705	2919 (51.2%)
11	892 (31.6%)	1930 (68.4%)	2822	5726	2904 (50.7%)
12	879 (30.2%)	2029 (69.8%)	2908	5865	2957 (50.4%)
13	816 (26.8%)	2229 (73.2%)	3045	6080	3035 (49.9%)
14	869 (27.2%)	2329 (72.8%)	3198	6449	3251 (50.4%)
15	829 (23.5%)	2702 (76.5%)	3531	6919	3388 (49%)
16	778 (19.9%)	3139 (80.1%)	3917	7635	3718 (48.7%)
17	760 (18.4%)	3379 (81.6%)	4139	7975	3836 (48.1%)
Total	13077 (27.5%)	34393 (72.4)	47470	92051	44581 (48.4%)

Footnote: Asymptomatic and symptomatic percentages calculated from all patients with known disease presentation; Missing information percentages calculated from all patients within an age bracket

For Review Only

SUPPLEMENTARY TABLE 3. DISTRIBUITION OF SYMPTOMS OF PAEDIATIC COVID-19 CASES

	Fever	Cough	Rhinorrhea	Dyspneia	Headache	Diarrhoea	Abdominal pain	Odynophagia	Nausea and Vomiting	Arthalgia	Myalgia	Tiredness and weakness	Tachycardia	Chest pain	Irritability	Convulsions
Total*	9737 (46.2%)	10016 (47.5%)	7633 (36.2%)	800 (3.8%)	7065 (33.5%)	2153 (10.2%)	1461 (6.9%)	4958 (23.5%)	1622 (7.7%)	160 (0.8%)	3552 (16.9%)	1642 (7.8%)	65 (0.3%)	432 (2%)	85 (0.4%)	38 (0.2%)
Symptoms**																
Fever		3721 (37,2%)	2902 (38%)	314 (39,3%)	2824 (40%)	1060 (49,2%)	845 (57,8%)	2046 (41,3%)	992 (61,2%)	81 (50,6%)	1615 (45,5%)	752 (45,8%)	52 (80%)	156 (36,1%)	56 (65,9%)	25 (65,8%)
Cough	3721 (38,2%)		4203 (55,1%)	584 (73%)	2724 (38,6%)	790 (36,7%)	430 (29,4%)	2310 (46,6%)	562 (34,6%)	74 (46,3%)	1600 (45%)	752 (45,8%)	30 (46,2%)	261 (60,4%)	40 (47,1%)	18 (42,9%)
Rhinorrhea	2902 (29,8%)	4203 (42%)		366 (45,8%)	1976 (28%)	568 (26,4%)	342 (23,4%)	1746 (35,2%)	415 (25,6%)	68 (42,5%)	1019 (28,7%)	565 (34,4%)	22 (33,8%)	144 (33,3%)	33 (38,8%)	11 (26,2%)
Dyspneia	314 (3,2%)	584 (5,8%)	366 (4,8%)		208 (2,9%)	77 (3,6%)	48 (3,3%)	183 (3,7%)	64 (3,9%)	9 (5,6%)	132 (3,7%)	118 (7,2%)	16 (24,6%)	102 (23,6%)	4 (4,7%)	1 (2,4%)
Headache	2824 (29%)	2724 (27,2%)	1976 (25,9%)	208 (26%)		616 (28,6%)	596 (40,8%)	2106 (42,5%)	578 (35,6%)	99 (61,9%)	2055 (57,9%)	857 (52,2%)	14 (21,5%)	205 (47,5%)	16 (18,8%)	13 (31%)
Diarrhoea	1060 (10,9%)	790 (7,9%)	568 (7,4%)	77 (9,6%)	616 (8,7%)		513 (35,1%)	326 (6,6%)	430 (26,5%)	23 (14,4%)	355 (10%)	220 (13,4%)	4 (6,2%)	45 (10,4%)	16 (18,8%)	6 (14,3%)
Abdominal pain	845 (8,7%)	430 (4,3%)	342 (4,5%)	48 (6%)	596 (8,4%)	513 (23,8%)		351 (7,1%)	436 (26,9%)	24 (15%)	252 (7,1%)	159 (9,7%)	11 (16,9%)	49 (11,3%)	10 (11,8%)	5 (11,9%)
Odynophagia	2046 (21%)	2310 (23,1%)	1746 (22,9%)	183 (22,9%)	2106 (29,8%)	326 (15,1%)	351 (24%)		319 (19,7%)	68 (42,5%)	1080 (30,4%)	460 (28%)	16 (24,6%)	135 (31,3%)	15 (17,6%)	12 (28,6%)
Nausea and Vomiting	992 (10,2%)	562 (5,6%)	415 (5,4%)	64 (8%)	578 (8,2%)	430 (20%)	436 (29,8%)	319 (6,4%)		24 (15%)	296 (8,3%)	228 (13,9%)	13 (20%)	55 (12,7%)	16 (18,8%)	5 (11,9%)
Arthalgia	81 (0,8%)	74 (0,7%)	68 (0,9%)	9 (1,1%)	98 (1,4%)	23 (1,1%)	24 (1,6%)	68 (1,4%)	24 (1,5%)		115 (3,2%)	56 (3,4%)	1 (1,5%)	13 (3%)	3 (3,5%)	0 (0%)
Myalgia	1615 (16,6%)	1600 (16%)	1019 (13,3%)	132 (16,5%)	2055 (29,1%)	355 (16,5%)	252 (17,2%)	1080 (21,8%)	296 (18,2%)	115 (71,9%)		648 (39,5%)	13 (20%)	124 (28,7%)	10 (11,8%)	6 (14,3%)
Tiredness and weakness	752 (7,7%)	752 (7,5%)	565 (7,4%)	118 (14,8%)	857 (12,1%)	220 (10,2%)	159 (10,9%)	460 (9,3%)	228 (14,1%)	56 (35%)	648 (18,2%)		10 (15,4%)	85 (19,7%)	20 (23,5%)	3 (7,1%)
Tachycardia	52 (0,5%)	30 (0,3%)	22 (0,3%)	16 (2%)	14 (0,2%)	4 (0,2%)	10 (0,7%)	16 (0,3%)	13 (0,8%)	1 (0,6%)	13 (0,4%)	10 (0,6%)		4 (0,9%)	5 (5,9%)	0 (0%)
Chest pain	156 (1,6%)	261 (2,6%)	144 (1,9%)	102 (12,8%)	202 (2,9%)	45 (2,1%)	49 (3,4%)	135 (2,7%)	55 (3,4%)	13 (8,1%)	124 (3,5%)	85 (5,2%)	4 (6,2%)		1 (1,2%)	0 (0%)
Irritability	56 (0,6%)	40 (0,4%)	33 (0,4%)	4 (0,5%)	16 (0,2%)	16 (0,7%)	10 (0,7%)	15 (0,3%)	15 (0,9%)	3 (1,9%)	10 (0,3%)	20 (1,2%)	5 (7,7%)	1 (0,2%)		3 (7,1%)
Convulsions	25 (0,3%)	18 (0,2%)	11 (0,1%)	1 (0,1%)	13 (0,2%)	6 (0,3%)	5 (0,3%)	12 (0,2%)	5 (0,3%)	0 (0%)	6 (0,2%)	3 (0,2%)	0 (0%)	0 (0%)	3 (3,5%)	

Footnote: *Percentages calculated from symptomatic patients with specified symptoms, **Percentages calculated from patients with column symptom

For peer review only

Confidential: For Review Only

BMJ Paediatrics Open

COVID-19 in Portugal: a retrospective review of paediatric cases, hospital and PICU admissions in the first pandemic year

Journal:	*BMJ Paediatrics Open*
Manuscript ID	bmjpo-2022-001499.R2
Article Type:	Original research
Date Submitted by the Author:	28-Jun-2022
Complete List of Authors:	Elias, Cecilia; Universidade de Lisboa, EPI Task- Force FMUL; USP Francisco George<br>Feteira-Santos, Rodrigo; Universidade de Lisboa, EPI Task-Force FMUL ; Instituto de Medicina Preventiva e Saúde Pública, Faculdade de Medicina, Universidade de Lisboa. , Avenida Professor Egas Moniz, 1649-028, Lisboa, Portugal.<br>Camarinha, Catarina; Universidade de Lisboa, EPI Task-Force FMUL<br>de Araújo Nobre, Miguel; Universidade de Lisboa, EPI Task-Force FMUL; FMUL, Clinica Universitaria Estomatologia<br>Costa, Andreia Silva; Universidade de Lisboa, EPI Task-Force FMUL; Universidade de Lisboa Instituto de Saúde Ambiental<br>Bacelar-Nicolau, Leonor; Universidade de Lisboa, EPI Task-Force FMUL; Universidade de Lisboa, FMUL - Institute of Preventive Medicine & ISAMB<br>Furtado, Cristina; Universidade de Lisboa, EPI Task-Force FMUL; Instituto Nacional de Saúde Doutor Ricardo Jorge<br>Nogueira, Paulo Jorge; Universidade de Lisboa, EPI Task- Force FMUL; Universidade de Lisboa Faculdade de Medicina, Instituto de Medicina Preventiva e Saúde Pública
Keywords:	COVID-19, Epidemiology, Mortality

SCHOLARONE™
Manuscripts

I, the Submitting Author has the right to grant and does grant on behalf of all authors of the Work (as defined in the below author licence), an exclusive licence and/or a non-exclusive licence for contributions from authors who are: i) UK Crown employees; ii) where BMJ has agreed a CC-BY licence shall apply, and/or iii) in accordance with the terms applicable for US Federal Government officers or employees acting as part of their official duties; on a worldwide, perpetual, irrevocable, royalty-free basis to BMJ Publishing Group Ltd ("BMJ") its licensees and where the relevant Journal is co-owned by BMJ to the co-owners of the Journal, to publish the Work in this journal and any other BMJ products and to exploit all rights, as set out in our licence.

The Submitting Author accepts and understands that any supply made under these terms is made by BMJ to the Submitting Author unless you are acting as an employee on behalf of your employer or a postgraduate student of an affiliated institution which is paying any applicable article publishing charge ("APC") for Open Access articles. Where the Submitting Author wishes to make the Work available on an Open Access basis (and intends to pay the relevant APC), the terms of reuse of such Open Access shall be governed by a Creative Commons licence – details of these licences and which Creative Commons licence will apply to this Work are set out in our licence referred to above.

Other than as permitted in any relevant BMJ Author's Self Archiving Policies, I confirm this Work has not been accepted for publication elsewhere, is not being considered for publication elsewhere and does not duplicate material already published. I confirm all authors consent to publication of this Work and authorise the granting of this licence.

Confidential: For Review Only

Article

COVID-19 in Portugal: a retrospective review of paediatric cases, hospital and PICU admissions in the first pandemic year

Cecília Elias [1,2*], Rodrigo Feteira-Santos [1,3,4,5], Catarina Camarinha [1], Miguel de Araújo Nobre [1,3,6], Andreia Silva Costa [1,5,7,8], Leonor Bacelar-Nicolau [1,3,4,5], Cristina Furtado [1,3,5,9], Paulo Jorge Nogueira [1,3,4,5]

1. EPI Task-Force FMUL, Faculdade de Medicina, Universidade de Lisboa. Avenida Professor Egas Moniz, 1649-028, Lisboa, Portugal.

2. Unidade de Saúde Publica Francisco George, ACES Lisboa Norte, ARSLVT

3. Instituto de Medicina Preventiva e Saúde Pública, Faculdade de Medicina, Universidade de Lisboa. Avenida Professor Egas Moniz, 1649-028, Lisboa, Portugal.

4. Área Disciplinar Autónoma de Bioestatística (Laboratório de Biomatemática), Faculdade de Medicina, Universidade de Lisboa. Avenida Professor Egas Moniz, 1649-028, Lisboa, Portugal.

5. Instituto de Saúde Ambiental, Faculdade de Medicina, Universidade de Lisboa. Avenida Professor Egas Moniz, 1649-028, Lisboa, Portugal.

6. Clínica Universitária de Estomatologia, Faculdade de Medicina, Universidade de Lisboa. Avenida Professor Egas Moniz, 1649-028 Lisboa, Portugal.

7. CIDNUR - Centro de Investigação, Inovação e Desenvolvimento em Enfermagem de Lisboa Escola Superior de Enfermagem de Lisboa. Avenida Professor Egas Moniz, 1600-190 Lisboa.

8. CRC-W—Católica Research Centre for Psychological, Family and Social Wellbeing, Universidade Católica Portuguesa, Palma de Cima, 1649-023 Lisboa, Portugal.

9. National Institute of Health Dr. Ricardo Jorge, Av. Padre Cruz, 1600-560 Lisboa, Portugal.

* **Correspondence**: cecilia.elias@gmail.com

Confidential: For Review Only

ABSTRACT

Background: COVID-19 is considered by WHO a pandemic with public health emergency repercussions. Children often develop a mild disease with good prognosis and the recognition of children at risk is essential to successfully manage paediatric COVID-19. Quality epidemiological surveillance data is required to characterize and assess the pandemic.

Methods: Data on all reported pediatric COVID-19 cases, in Portugal, was retrospectively assessed from a fully anonymized dataset provided by the Directorate-General for Health (DGS). Pediatric hospital admission results were obtained from the DGS vaccine recommendations and paediatric intensive care unit (PICU) admission results from the Epicentre.pt group. Reported cases and PICU admissions from March 2020 to February 2021 and hospital admissions between March and December 2020 were analyzed.

Results: 92,051 COVID-19 cases were studied, 50.5% males, average age of 10.1 years, corresponding to 5.4% of children in Portugal. The most common symptoms were cough and fever, whereas gastrointestinal symptoms were infrequent. The most common comorbidity was asthma. A high rate of missing surveillance data was noticed, on presentation of disease and comorbidity variables, which warrants a cautious interpretation of results. Hospital admission was required in 0.93% of cases and PICU on 3.48 per 10,000 cases. PICU admission for Multisystem Inflammatory Syndrome in Children was more frequent in children with no comorbidities and males, severe COVID-19 was rarer and occurred mainly in females and infants. Case fatality rate and mortality rates were low, 1.8 per 100,000 cases and 1.2 per 1,000,000 respectively.

Conclusions: The overall reported case incidence was 5.4 per 100 children and adolescents and less than 1% of cases required hospital admission. MIS-C was more frequent in patients with no comorbidities and males. Mortality and case-fatality rates were low. Geographic adapted strategies, and information systems to facilitate surveillance, policy decision making, audit and research are required to improve surveillance data.

Confidential: For Review Only

Conclusions: The clinical presentations predominantly respiratory and neurologic conversely gastrointestinal symptoms were infrequent. Less than 1% of cases required hospital admission. MIS-C was more frequent in patients with no comorbidities and males. Mortality and case-fatality rates were low. Geographic adapted strategies, and information systems to facilitate surveillance, policy decision making, audit and research are required to improve surveillance data.

Confidential: For Review Only

Keywords: COVID-19, Pediatric, Epidemiology, Hospital Admission, MIS-C, PICU, Health surveillance data

Confidential: For Review Only

What we know about this subject:

- Quality health surveillance data is essential assess the COVID-19 pandemic.
- Children present a milder course of disease and a lower susceptibility to being severely affected by COVID-19.
- Globally pediatric COVID-19 case-fatality and mortality rates are low.

What our study adds:

- Portuguese paediatric surveillance data and systems need improvement.
- Paediatric COVID-19 incidence was 5.4% during the first pandemic year.
- Portuguese children and adolescents presented an extremely low mortality and case-fatality rate, 1.8 per 100,000 cases and 1.2 per 1,000,000 respectively.

BACKGROUND

Coronavirus disease 2019 (COVID-19), caused by severe acute respiratory syndrome coronavirus 2 (SARS-CoV-2), is considered by the World Health Organization (WHO) a pandemic with public health emergency repercussions.[1],[2] Worldwide, millions of people have been infected with COVID-19 since the end of 2019 among all age groups.[3] Children are also at risk for COVID-19 but have experienced a lower incidence than other age groups.[4,5] Laboratory-confirmed COVID-19 cases in the United States reveal that only 1.7% of cases were among children and adolescents under 18 years old.[6] Furthermore, several studies showed these age groups have lower susceptibility to being severely affected, often developing a mild form of disease and with a likely good prognosis.[7–9] Studies have shown that children present different symptoms, distinct comorbidities, decreased disease severity and fewer disease complications.[10] The characteristics and clinical manifestations of infected children[11],[12] have been considered of major interest.[13] The most frequent symptoms described in children have been fever, cough[14] and ear, nose, and throat complaints. However, other manifestations such as gastrointestinal symptoms have also been identified.[12,15,16] A small number of children needed hospital admission, and of these, a minority required ICU admission either due to severe COVID-19 or multisystem inflammatory disease in children (MIS-C).[17,18] Several risk factors have been associated with these conditions such as extreme age, male sex, comorbidities and non-white background.[19]

As COVID-19 characteristics and clinical course among children can differ from adult patients in numerous ways, specific recommendations for the diagnosis, treatment and vaccination plans in children are being implement worldwide, despite differences across countries.[20,21,22,23]Increasing knowledge of the clinical characteristics in this demographic group may also contribute towards the development and continuous adaptation of preventive strategies that can benefit all communities. Epidemiological surveillance data is essential to attain these objectives. Epidemiological surveillance is a critical part of public health practice[24] where continuous collection, analysis, and interpretation of health data helps identify disease clusters, assess health trends, monitor interventions, identify high risk groups and guide decision making and action.[25] Furthermore, epidemiological data quality is essential and should be monitored to guarantee data results are meaningful.[26]

Confidential: For Review Only

This study aimed to investigate the epidemiological and clinical characteristics of pediatric SARS-CoV-2 infection cases, hospital and ICU admissions, compare these to other countries and to assess the quality of epidemiological surveillance data in Portugal.

MATERIALS AND METHODS

STUDY DESIGN AND DATA

Data on all cases of COVID-19 in children and adolescents under the age of 18, in Portugal, diagnosed between March 2nd, 2020, and February 28th, 2021 were retrospectively assessed. The dataset was fully anonymized and was provided by the Directorate-General for Health (DGS) on 11th of March 2021. The data was collected by the SINAVE (National Epidemiological Surveillance System). The SINAVE is an online system aimed at the electronic notification of mandatory reporting diseases in Portugal. The SINAVE database includes demographic and clinical information collected from all reported patients. Epidemiological Surveillance data in SINAVE is meant to be filled by public health doctors and nurses. However, due to the COVID-19 pandemic increase workload, other professionals from a diverse background have also been recruited to support in surveillance. According to the WHO definition, a confirmed SARS-CoV-2 case was based on a positive polymerase chain reaction test.[27] Hospital admissions with a SARS-CoV-2 primary or secondary diagnosis, from March 2nd to December 31st 2020, were obtained as results from the Pediatric COVID-19 vaccination technical recommendation by DGS.[28] These results include admissions to all hospitals in Portugal with a SARS-CoV-2 diagnosis. ~~The National Health Service Hospitals (SNS), freely cover the paediatric population in the country.~~ No information on whether the SARS-CoV-2 diagnosis was the primary or secondary cause of admission was available. Data on pediatric SARS-CoV-2 PICU admissions, from March 2020 to February 2021, were obtained as anonymized data results on severe COVID-19 [29] and Multisystem inflammatory syndrome in children (MIS-C)[30] from the Epicentre.pt work group. Daily death reports published by DGS were analyzed to calculate the number of deaths caused by COVID-19 and these overlapped with the recorded SINAVE case deaths. Data from the National Statistics Institute (INE) was used for mortality and population comparisons. The pediatric mid 2020 population was used by proxy as an estimate population for the period analyzed. Patients and public were not involved in the design or analysis developed in this study.

Confidential: For Review Only

VARIABLES

The SINAVE system includes two variables which indicate the patient's clinical presentation (as symptomatic or asymptomatic) and comorbidities (present, absent). Information on symptoms and comorbidities associated with each case notification should also be filled in through individual variables. For a given symptom or comorbidity, health professionals selected the respective field as yes or no, according to the patient's information. However, any information not filled, for a given symptom or comorbidity, created a missing value in the database. Considering this, a new variable was calculated to describe if a patient was symptomatic or not, using the patient's clinical presentation variable and associated symptoms variables and similarly for the presence of comorbidities. A patient was considered symptomatic either if health professionals classified it as symptomatic through the SINAVE original variable or at least one symptom was reported. In addition, those with any symptom variable filled in but not previously recorded as symptomatic were considered symptomatic patients. Patients' symptomatology status was missing when no symptom variables were filled, and no information regarding the presentation of symptoms in the original variable. A patient was considered having comorbidities either if the presence of comorbidities was selected through the SINAVE original variable or at least one comorbidity was reported.

Statistical analysis

Descriptive statistics were used to summarize univariate characteristics. Results were presented as absolute and relative frequencies for qualitative variables, and as means, medians and standard deviations (SD) for quantitative variables. Calculated percentages in have excluded missing cases unless otherwise stated. ~~Differences between groups were evaluated by Chi-square test with significance level set at 5%.~~ All analysis were conducted using R software version 3.6.3 (R Foundation for Statistical Computing, Vienna, Austria) and IBM SPSS Statistics 28.0.1.1 (14).

RESULTS

GENERAL CHARACTERISTICS

The present study included 92,051 COVID-19 cases in children and adolescents reported from March 2nd, 2020, to February 28th, 2021, in Portugal; 46,453 (50.5%) were males, and 45583 (49.5%) females,

Confidential: For Review Only

or the overall reported case incidence was 5.4 overall reported case rate of 5.4 per 100 children and adolescents and corresponding to 5.3% of males and 5.5% of females. The age profile of patients was distributed between 0 and 17 years old, with the number of cases increasing with age (Table 1). An average age of 10.1 (standard deviation of 5.0) and a median of 11.0 years were observed.

The geographical distribution highlights most cases occurred in 3 urban areas, the Lisbon district (n=22,999, 25%), followed by Porto (n=18,350, 19.9%) and Braga (n=11,091, 12%). The areas with fewer cases were predominantly rural districts (Portalegre and Beja) and autonomous regions of the Azores and Madeira (Figure 1). The children and adolescents with COVID-19 had mostly Portuguese nationality (n = 88,387, 95.3%). Only 4% were from other backgrounds, mainly from Brazil, Angola, Cape Verde, São Tome and Principe and scattered from a wide range of countries.

COVID-19 CASES TIMELINE

During this study, several COVID-19 epidemic waves were observed (Figure 2): 1) between March and April 2020, when 738 cases were reported (0.8% of all cases); 2) between May and July 2020, when 1756 cases (1.9% of all cases) were reported; 3) from September to December 2020, when 39897 cases (43.3% of all cases) were reported; and 4) from January to March 2021, when 46,634 cases (50.7% of all cases) were reported.

CLINICAL PRESENTATION

Information on disease presentation, as symptomatic or asymptomatic, was reported in 47,470 (52%) of cases while there was no information regarding the presence or absence of symptoms in 44,192 cases (48%). From cases with information on clinical presentation, 13,077 (27.5%) were asymptomatic and 34,393 (72.5%) symptomatic. Specific symptoms were reported in 21,077 (61.3%44.4%) of cases and a further 13,316 (38.7%14.5%) where recorded as symptomatic without reporting symptoms. The asymptomatic presentation of the disease increased with age, peaking between 4 and 10 years of age and slightly decreasing subsequently.

Confidential: For Review Only

To describe the symptomatic presentations an analysis which ~~exclusively~~ included only cases where symptoms were recorded was performed (n = 21,077). The most common symptoms reported were cough (n = 10,205, 48.4%), fever (n = 9,812, 46.6%), rhinorrhea (n = 7,739, 36.7%), and headache (n = 7,143, 33.9%). Gastrointestinal symptoms, such as abdominal pain, nausea and vomiting and diarrhea were reported in 7.1%, 7.8% and 10.2% respectively. Analyzing the distribution of the co-occurrence of clinical COVID-19 symptoms, in each pediatric patient, fever was commonly combined with cough (38.2%), rhinorrhea (29.8%), headache (29%), odynophagia (21%) or myalgia (16.6%). Furthermore, combinations of these symptoms were the most common presentations reported. Fever, cough, and rhinorrhea were the most common presentations for children from 0 to 9 (Table 2). Children aged 0 presented with the highest rates of fever (48%), cough (37%), rhinorrhea (35%) and diarrhea (24%) when compared to the other age groups. From age 10-17 years, headache was the most common reported symptom, followed by fever, cough and rhinorrhea.

COMORBIDITIES

Information on the presence or absence of comorbidities was reported in 19,680 (21.4%) of cases and there was no information regarding the presence or absence of comorbidities~~symptoms~~ in 72,371 (78.6%). A total of 1,593 patients presented with comorbidities, of which 1,000 were specified. The three most frequently reported comorbidities ~~associated with COVID-19~~ in children and adolescents with COVID-19 were: asthma (n = 753, 0.8%), neuromuscular diseases (n = 49) and diabetes (n = 55). Most patients with asthma or neurologic disease were males, whereas most patients with diabetes were females. (Table 3)

HOSPITAL AND PICU ADMISSIONS AND DEATHS

A total of 476 children were admitted to hospital with a COVID-19 ICD-10 code, either as primary or secondary diagnosis. Children aged from 0 – 4 represented 47.9% (228) of hospital admissions, corresponding to 2.24% of all Covid-19 cases in this age group during this time. Overall, 2.8 per 10,000

Confidential: For Review Only

children in Portugal required hospital admission (Table 4). No other sociodemographic or clinical variables were available to further characterize this population.

A total of 32 children required PICU admission, corresponding to 0.19 PICU admissions per 10,000 children and adolescents in Portugal. Of these, 28 (87.5%) were admitted due to MIS-C and 4 (12.5%) due to severe COVID-19. MIS-C patients were predominantly male (21, 75%) with ages from 15 months to 17 years age, average of 10.75 and median of 10.5 years. Most cases occurred between January and February 2021. Six patients presented comorbidities. Severe COVID-19 patients were mainly females, under the age of one and all presenting with comorbidities. Two deaths caused by COVID-19 were reported by the DGS (the National Health Authority) during this study and coincided with two deaths recorded in the SINAVE database.

.

Confidential: For Review Only

Discussion

In this study, epidemiological and clinical characteristics of pediatric COVID-19 infections, hospital and PICU admissions in Portugal were described. Health surveillance information analysis revealed an essential and urgent need for improvement.

General Characteristics - Although there is accessible and exhaustive knowledge regarding adult COVID-19 disease[31],[32], pediatric COVID-19 epidemiology and clinical characteristics are significantly less studied and documented in the literature. SARS-CoV-2 infection studies have shown that children are more likely to be asymptomatic than adults [33] and present a less severe disease course.[34 35] Young children and infants are reported as more vulnerable to infection [36] than older pediatric patients. COVID-19 related fatalities have been reported as scarce in the pediatric population.[37] In this context, we assessed the characteristics and clinical features of COVID-19 in children in Portugal.

We observed an increase of COVID-19 cases with age in both sexes with slightly higher number of cases in males compared to females (50.5% vs. 49.5%). Overall, in the scientific literature, males are most affected, although individual smaller studies have shown either sex as being the most frequent.[38],[35]

Most of these cases were geographically clustered in the high-density urban areas of Lisbon, Porto, and Braga. The association between population density and cumulative infection cases spread was previously observed at a county level in the United States of America and considered an effective predictor of cumulative infection cases.[39] The regions with lower number of cases were mainly rural and low-density populated districts, such as Portalegre, Beja and the autonomous regions of the Azores and Madeira. The nationality of COVID-19 pediatric patients reflects the population's background. The majority were Portuguese and 4% come from diverse backgrounds such as Brazil, Angola, Cape Verde, and São Tome and Principe, countries with historical ties and emigration patterns to Portugal.

Several COVID-19 waves were observed in our study. Following the first wave, in March and April 2020, the first State of Emergency [40], and lockdown were implemented, including school closures, mandatory remote work, limiting mobility, business hours, and social gatherings. Between March 17th and April 2nd, all children who attended the Emergency Department (ED) of a Portuguese hospital with suspected

Confidential: For Review Only

COVID-19 symptoms as cough, fever or rhinorrhoea were tested. Of all 94 cases, one was positive.[41] This can indicate a low incidence of disease at this stage which is in accordance with our results. Studies on antibody seroprevalence of SARS-CoV-2 have shown that only half the children and adolescents had developed disease symptoms.[42]

Clinical Presentation and Comorbidities - The most common symptoms identified (cough, fever, rhinorhoea and headache) have been dominant in meta-analysis describing clinical characteristics of children: Wang et al. reported the presence of fever in 48% and cough 39% of the cases.[43] Li et al. in 7004 paediatric cases reported fever 47% and cough 42% as the most prevalent clinical symptoms [44]. The presence of gastrointestinal symptoms has been identified as major symptoms occurring in the pediatric population.[45,46,47] In our analysis, diarrhea, abdominal pain, nausea and vomiting were less frequent than other symptoms like cough, fever and rhinorrhea as has been reported in other studies.[4,35] Interestingly, the most common symptoms per age groups can be organized in 3 sets: 1) from ages zero to four years: fever, cough and rhinorrhea; 2) from ages five to nine years: fever, cough, rhinorrhea and headache; and 3) from 10 to 17 years: the previous 4 symptoms, odynophagia and myalgia. This result can reflect simultaneously the inability of young children to express their complaints and an increase in the variety of symptoms and presentations as age increases.

Asthma was the most common underlying condition and has been the most reported comorbidity among several pediatric cases as well.[37,48,49]. Verma et al. analysed SARS-CoV-2 infections in children in four hospitals in the New York City area and found those with asthma were more likely to need respiratory support during hospitalization.[50] In a systematic review, including 5686 children aged under 18 years with SARS-CoV-2 infection, the most frequent comorbidity was cardiac disease; and most children who required ventilation had underlying comorbidities.[4] A study of ED and admitted pediatric COVID-19 patients at a Level 3 Lisbon Hospital, one of two reference hospitals for COVID-19 pediatric patients at this point in the pandemic, showed 10% had risk factors for disease, 20% were asymptomatic, 43% presented with fever and 42% with respiratory symptoms.[51] Though the referred study is likely representing the most symptomatic COVID-19 cases seeking medical help, whereas our study focuses on all pediatric COVID-19 cases, chronic respiratory disease was also the most common comorbidity. Only a small proportion of children with COVID-19 had registered comorbidities (n = 1593, 2%) which is lower than reported in other studies[49] and of what the authors expected.

Confidential: For Review Only

Missing Surveillance Data Analysis - The quality of surveillance data was assessed trough a missingness analysis, given the abnormally high number of missing clinical presentations (48%) and comorbidities (98%). Given the abnormally high number of missing clinical presentations (48%, n = 44,177) and comorbidities (98%, n = 72,371) the quality of surveillance data was assessed. Statistical significant dData results progressively worsened with the pandemic and particularly when case numbers sharply increased. These results can be consequential of massive work overload observed in the public health units combined with short staff. Furthermore, with increasing case counts, non-public health professionals were recruited to support the units. However, significant asymmetries were also evident according to district, with substantial fluctuations in recording rates across the country. The two districts with the highest recorded data were Lisbon and Faro whereas the lowest recorded on disease presentation where Guarda and Bragança and on the presence of comorbidities where Évora, Bragança, Guarda and Portalegre. Notwithstanding, good practice and the SINAVE guidelines warrant all fields to be answered and to achieve this goal measures and geographically adapted strategies, such as increased training, resources, and continued auditing, need to be implemented to improve data surveillance. The SINAVE system collects symptoms and comorbidities in a non-mandatory and non-intelligent way, not tailored to previous answers. This system also needs to be improved from a user perspective so to fast-track user experience and enhance clinical record keeping. A problem with Portuguese surveillance data collection system needs to be recognized so it can be improved with increased training, geographic adapted strategies, and information systems to facilitate adequate epidemiological surveillance collection. This data is essential to guide public health strategies, policy decision making, audit and research.

Hospital and PICU admissions - In 2020, a total of 476 children were admitted to the hospital from 51,153 cases reported, corresponding to 1 in 108 cases requiring hospitalization, a rate of 0.93% of cases nationwide and a paediatric hospitalization rate of 2.8 per 10,000 children and adolescents. A lower case hospitalization result than reported in neighbouring Navarra, Spain (3.6%) during the first wave[52], in the United States (5.3%)[53] and in Scotland, where 1.03% of cases in children aged 5- to 17-year-olds required hospitalization[54]. These results probably might reflect a higher testing rate of asymptomatic or mildly ill children in Portugal.

Confidential: For Review Only

Thirty-two cases required PICU admission, corresponding to a rate of admission of 3.48 per 10,000 cases. Four cases (12.5%) Four cases (females under the age of one year and presenting comorbidities; 12.5%) were admitted with severe COVID-19 (non-MIS-C) and twenty-eight (87.5%) with MIS-C, implying that in our population, severe COVID-19 in children is less frequent than MIS-C. Patients with severe COVID-19 were predominantly female under one with comorbidities, whereas MIS-C patients were predominantly male and the majority (75%) had no comorbidities, which is similar to previously published results, where most MIS-C patients were healthy, and no correlation was observed between comorbidity score and MIS-C occurrence.[29]

Deaths - During this period, the Directorate-General of Health confirmed two paediatric deaths caused by COVID-19, corresponding to a case-fatality rate of approximately 1.8 per 100,000 cases. Furthermore, analysing specifically total paediatric deaths in Portugal in 2020 (n = 387) and comparing to the paediatric COVID-19 deaths, COVID-19 was the main cause of death in 0.52%. The mortality rate due to COVID-19 was 1.2 per million children and adolescents. These results find parallel to similar studies, highlighting the low mortality and case-fatality rates. Despite the extremely small number of deaths, it is reasonable to compare these results with other countries. Bhopal et al reported an overall mortality rate due to COVID-19 in children of 1.7 per 10.000, until February 2021 when comparing seven countries. Smith et al.[55] reported a mortality rate due to COVID-19 of 2 per million and fatality rate of 5 per 100,000 in England, between March 2020 and February 2021, a result 1.7 and 2.5 times higher than the one we report, respectively. Spanish mortality results were published by Tagarro et al.[56] and reported 0.21 per 100 000 in children aged 0 to 9 years and 0.34 per 100 000 in children aged 10 to 19 years. These results may reflect differences in shielding of vulnerable population or lockdown policies implemented in these countries, but further studies are required to analyse these effects.

The major strengths of our study are the combined analysis of a large database of COVID-19 cases, hospital and PICU admissions and deaths. These results present a description of paediatric COVID-19 in Portugal and allow comparison with other countries.

The authors found the elevatedhigh level of missingness in surveillance data unusual. The analysis of real-world data has important challenges as previously acknowledged[57], however other studies using

Confidential: For Review Only

national data have reported similar difficulties[58,59]. Despite the authors best effort in this analysis, less than optimal real-world data has imposed important limitations to the study and hindered some of its quality, particularly relating to missing values in some of the analyzed variables. The authors are developing further studies and proposing solutions to help prevent these issues.[58,59] As stated, the major limitations of this analysis are data-related, ~~besides the level of missingness observed,~~ the possible underreporting of cases and its characteristics, such as symptoms and comorbidities, the limited access to hospital admission data and deaths and the impracticality of establishing a chronological order of events. Furthermore, the estimated population used for comparison was obtain by-proxy with the reported paediatric for 2020.

CONCLUSIONS

The overall reported case incidence was 5.4 per 100 children and adolescents and less than 1% of cases required hospital admission. ~~The clinical manifestations of disease reported in our analysis were predominantly respiratory and neurologic, while gastrointestinal symptoms were infrequent. Less than 1% of patients required hospital admission.~~ MIS-C was more frequent in patients with no comorbidities and males, severe COVID-19 was rarer and occurred mainly in children under 1 year of age and females, all with comorbidities. Case fatality rates and mortality rates were low when comparing with other countries. This study highlights the need to improve epidemiological surveillance data in Portugal, with geographic adapted strategies, and information systems to facilitate adequate epidemiological surveillance, policy decision making, audit and research.

Confidential: For Review Only

Acknowledgements

The authors would like to thank Dr. Cristina Camilo and the EPICENTRE.PT research group.

Funding

Funds from Fundação para a Ciência e a Tecnologia to the Instituto de Saúde Ambiental, Faculdade de Medicina, Universidade de Lisboa (UIDB/04295/2020).

Conflict of Interests

The authors declare no conflict of interest.

Contributorship Statement

Cecilia Elias planned the study and was the guarantor. She developed the methodology and was involved in obtaining the data, data analysis, writing the manuscript, creating tables/figures and reviewing the text. Rodrigo Feteira-Santos was involved in obtaining data, writing the manuscript, creating tables and figures and reviewing the text. Catarina Camarinho was involved in planning the study, creating tables and figures, writing and reviewing the manuscript and maintaining references. Miguel de Araújo Nobre was involved in writing and reviewing the manuscript. Andreia Costa was involved in writing and reviewing the manuscript. Leonor Bacelar was involved in writing and reviewing the manuscript. Cristina Furtado was involved in writing and reviewing the manuscript. Paulo Nogueira was involved in planning the study, data analysis and writing and reviewing the manuscript.

Confidential: For Review Only

Confidential: For Review Only

References

1. Cucinotta D, Vanelli M. WHO Declares COVID-19 a Pandemic. *Acta bio-medica : Atenei Parmensis*. 2020;91(1):157-160. doi:10.23750/abm.v91i1.9397

2. WHO/Europe | Coronavirus disease (COVID-19) outbreak - WHO announces COVID-19 outbreak a pandemic.

3. Wu Z, McGoogan JM. Characteristics of and Important Lessons From the Coronavirus Disease 2019 (COVID-19) Outbreak in China: Summary of a Report of 72 314 Cases From the Chinese Center for Disease Control and Prevention. *JAMA*. 2020;323(13):1239-1242. doi:10.1001/jama.2020.2648

4. Williams N, Radia T, Harman K, Agrawal P, Cook J, Gupta A. COVID-19 Severe acute respiratory syndrome coronavirus 2 (SARS-CoV-2) infection in children and adolescents: a systematic review of critically unwell children and the association with underlying comorbidities. *European Journal of Pediatrics*. 2021;180(3):689-697. doi:10.1007/s00431-020-03801-6

5. Stokes EK, Zambrano LD, Anderson KN, Marder EP, Raz KM. Coronavirus Disease 2019 Case Surveillance — United States, January 22–May 30, 2020. *MMWR Morb Mortal Wkly Rep 2020*. 2020;69(24):759-765.

6. Bialek S, Gierke R, Hughes M, McNamara LA, Pilishvili T, Skoff T. Coronavirus disease 2019 in children: Current status - Morbidity and Mortality Weekly Report. 2020;69(14):422-426.

7. Dong Y, Mo X, Hu Y, et al. Epidemiology of COVID-19 Among Children in China. *Pediatrics*. 2020;145(6):20200702. doi:10.1542/PEDS.2020-0702

8. Mantovani A, Rinaldi E, Zusi C, Beatrice G, Saccomani MD, Dalbeni A. Coronavirus disease 2019 (COVID-19) in children and/or adolescents: a meta-analysis. *Pediatric research*. 2021;89(4):733-737. doi:10.1038/s41390-020-1015-2

9. Götzinger F, Santiago-García B, Noguera-Julián A, et al. COVID-19 in children and adolescents in Europe: a multinational, multicentre cohort study. *The Lancet Child & adolescent health*. 2020;4(9):653-661. doi:10.1016/S2352-4642(20)30177-2

10. Zare-Zardini H, Soltaninejad H, Ferdosian F, Hamidieh AA, Memarpoor-Yazdi M. Coronavirus Disease 2019 (COVID-19) in Children: Prevalence, Diagnosis, Clinical Symptoms, and Treatment. *International journal of general medicine*. 2020;13:477-482. doi:10.2147/IJGM.S262098

11. Guo CX, He L, Yin JY, et al. Epidemiological and clinical features of pediatric COVID-19. *BMC Medicine*. 2020;18(1):1-7. doi:10.1186/s12916-020-01719-2

Confidential: For Review Only

12. Zimmermann P, Curtis N. Coronavirus infections in children including COVID-19: An overview of the epidemiology, clinical features, diagnosis, treatment and prevention options in children. *Pediatric Infectious Disease Journal*. 2020;39(5):355-368. doi:10.1097/INF.0000000000002660

13. Davies NG, Klepac P, Liu Y, et al. Age-dependent effects in the transmission and control of COVID-19 epidemics. *Nature Medicine*. 2020;26(8):1205-1211. doi:10.1038/s41591-020-0962-9

14. Wang Z, Zhou Q, Wang C, et al. Clinical characteristics of children with COVID-19: a rapid review and meta-analysis. *Annals of translational medicine*. 2020;8(10):620. doi:10.21037/atm-20-3302

15. Mayor S. Covid-19: UK studies find gastrointestinal symptoms are common in children. *BMJ*. 2020;370. doi:10.1136/bmj.m3484

16. Giacomet V, Barcellini L, Stracuzzi M, et al. Gastrointestinal symptoms in severe covid-19 children. *Pediatric Infectious Disease Journal*. 2020;39(10):E317-E320. doi:10.1097/INF.0000000000002843

17. Borrelli M, Corcione A, Castellano F, Fiori Nastro F, Santamaria F. Coronavirus Disease 2019 in Children. *Frontiers in Pediatrics*. 2021;9. doi:10.3389/fped.2021.668484

18. Patel NA. Pediatric COVID-19: Systematic review of the literature. *American Journal of Otolaryngology - Head and Neck Medicine and Surgery*. 2020;41(5). doi:10.1016/j.amjoto.2020.102573

19. Ward JL, Harwood R, Smith C, et al. Risk factors for PICU admission and death among children and young people hospitalized with COVID-19 and PIMS-TS in England during the first pandemic year. *Nature medicine*. 2022;28(1):193-200. doi:10.1038/s41591-021-01627-9

20. Maltezou HC, Vorou R, Papadima K, et al. Transmission dynamics of SARS-CoV-2 within families with children in Greece: A study of 23 clusters. *Journal of medical virology*. 2021;93(3):1414-1420. doi:10.1002/jmv.26394

21. Han YN, Feng ZW, Sun LN, et al. A comparative-descriptive analysis of clinical characteristics in 2019-coronavirus-infected children and adults. *Journal of medical virology*. 2020;92(9):1596-1602. doi:10.1002/jmv.25835

22. Bi Q, Wu Y, Mei S, et al. Epidemiology and transmission of COVID-19 in 391 cases and 1286 of their close contacts in Shenzhen, China: a retrospective cohort study. *The Lancet Infectious diseases*. 2020;20(8):911-919. doi:10.1016/S1473-3099(20)30287-5

Confidential: For Review Only

23. Kawasuji H, Takegoshi Y, Kaneda M, et al. Transmissibility of COVID-19 depends on the viral load around onset in adult and symptomatic patients. *PloS one*. 2020;15(12):e0243597. doi:10.1371/journal.pone.0243597

24. Ibrahim NKR, Al Bar HM. Surveillance of childhood vaccine-preventable diseases at health facilities in Jeddah, Saudi Arabia. *Eastern Mediterranean health journal = La revue de sante de la Mediterranee orientale = al-Majallah al-sihhiyah li-sharq al-mutawassit*. 2009;15(3):532-543.

25. Choi BCK. The Past, Present, and Future of Public Health Surveillance. Eriksson K, Mastrangelo G, eds. *Scientifica*. 2012;2012:875253. doi:10.6064/2012/875253

26. European Centre for Disease Prevention and Control. *Data Quality Monitoring and Surveillance System Evaluation – A Handbook of Methods and Applications*. Stockholm: ECDC; 2014.

27. World Health Organization. Laboratory testing for 2019 novel coronavirus (2019-nCoV) in suspected human cases. *WHO - Interim guidance*. 2020;2019(January):1-7.

28. Direção Geral da Saúde (DGS). Comissão Técnica de Vacinação contra a COVID-191 Vacinação contra a COVID-19 em crianças com 5 a 11 anos.

29. Preston LE, Chevinsky JR, Kompaniyets L, et al. Characteristics and Disease Severity of US Children and Adolescents Diagnosed With COVID-19. *JAMA Network Open*. 2021;4(4):e215298-e215298. doi:10.1001/jamanetworkopen.2021.5298

30. World Health Organization (WHO). Living guidance for clinical management of COVID-19: Living guidance, 23 November 2021.

31. Lechien JR, Chiesa-Estomba CM, Place S, et al. Clinical and epidemiological characteristics of 1420 European patients with mild-to-moderate coronavirus disease 2019. *Journal of Internal Medicine*. 2020;288(3):335-344. doi:https://doi.org/10.1111/joim.13089

32. Zhou F, Yu T, Du R, et al. Clinical course and risk factors for mortality of adult inpatients with COVID-19 in Wuhan, China: a retrospective cohort study. *The Lancet*. Published online 2020. doi:10.1016/S0140-6736(20)30566-3

33. Adeyinka A, Bailey K, Pierre L, Kondamudi N. COVID 19 infection: Pediatric perspectives. *Journal of the American College of Emergency Physicians Open*. 2021;2(1):1-11. doi:10.1002/emp2.12375

34. Dong Y, Dong Y, Mo X, et al. Epidemiology of COVID-19 among children in China. *Pediatrics*. 2020;145(6). doi:10.1542/peds.2020-0702

Confidential: For Review Only

35. Patel NA. Pediatric COVID-19: Systematic review of the literature. *American Journal of Otolaryngology - Head and Neck Medicine and Surgery*. 2020;41(5):102573. doi:10.1016/j.amjoto.2020.102573

36. Dong Y, Mo X, Hu Y, et al. Epidemiology of COVID-19 Among Children in China. *Pediatrics*. 2020;145(6). doi:10.1542/peds.2020-0702

37. Bialek S, Gierke R, Hughes M, McNamara LA, Pilishvili T, Skoff T. Coronavirus disease 2019 in children: Current status - Morbidity and Mortality Weekly Report. 2020;69(14):422-426.

38. Götzinger F, Santiago-García B, Noguera-Julián A, et al. COVID-19 in children and adolescents in Europe: a multinational, multicentre cohort study. *The Lancet Child & adolescent health*. 2020;4(9):653-661. doi:10.1016/S2352-4642(20)30177-2

39. Wong DWS, Li Y. Spreading of COVID-19: Density matters. *PLOS ONE*. 2020;15(12):e0242398.

40. Diário da República 1.ª série. *Decreto Do Presidente Da República n.º 14-A/2020 de 18 de Março.*; 2020.

41. Costa A, Almeida H, Moniz M, Alves C. COVID-19 screening in a Portuguese pediatric population. *Enfermedades Infecciosas y Microbiologia Clinica*. 2020;(xx):9-12. doi:10.1016/j.eimc.2020.09.004

42. Felsenstein S, Hedrich CM. SARS-CoV-2 infections in children and young people. *Clinical Immunology*. 2020;220:108588. doi:https://doi.org/10.1016/j.clim.2020.108588

43. Wang Z, Zhou Q, Wang C, et al. Clinical characteristics of children with COVID-19: a rapid review and meta-analysis. *Annals of translational medicine*. 2020;8(10):620. doi:10.21037/atm-20-3302

44. Li B, Zhang S, Zhang R, Chen X, Wang Y, Zhu C. Epidemiological and Clinical Characteristics of COVID-19 in Children: A Systematic Review and Meta-Analysis. *Frontiers in pediatrics*. 2020;8:591132. doi:10.3389/fped.2020.591132

45. Waterfield T, Watson C, Moore R, et al. Seroprevalence of SARS-CoV-2 antibodies in children: a prospective multicentre cohort study. *Archives of Disease in Childhood*. 2021;106(7):680-686. doi:10.1136/archdischild-2020-320558

46. Akobeng AK, Grafton-Clarke C, Abdelgadir I, Twum-Barimah E, Gordon M. Gastrointestinal manifestations of COVID-19 in children: a systematic review and meta-analysis. *Frontline Gastroenterology*. 2021;12(4):332-337. doi:10.1136/flgastro-2020-101529

Confidential: For Review Only

47. Al-Beltagi M, Saeed NK, Bediwy AS, El-Sawaf Y. Paediatric gastrointestinal disorders in SARS-CoV-2 infection: Epidemiological and clinical implications. *World journal of gastroenterology*. 2021;27(16):1716-1727. doi:10.3748/wjg.v27.i16.1716

48. Stokes EK, Zambrano LD, Anderson KN, Marder EP, Raz KM. Coronavirus Disease 2019 Case Surveillance — United States, January 22–May 30, 2020. *MMWR Morb Mortal Wkly Rep 2020*. 2020;69(24):759-765.

49. Patel NA. Pediatric COVID-19: Systematic review of the literature. *American Journal of Otolaryngology - Head and Neck Medicine and Surgery*. 2020;41(5):102573. doi:10.1016/j.amjoto.2020.102573

50. Verma S, Lumba R, Dapul HM, et al. Characteristics of Hospitalized Children With SARS-CoV-2 in the New York City Metropolitan Area. *Hospital Pediatrics*. 2021;11(1):71-78. doi:10.1542/hpeds.2020-001917

51. Picão de Carvalho C, Castro C, Sampaio Graça I, et al. Série de Casos de 103 Crianças com Infeção por SARS-CoV-2 em Portugal. *Acta Médica Portuguesa*. 2020;33(12):795. doi:10.20344/amp.14537

52. Moreno-Galarraga L, Urretavizcaya-Martínez M, Alegría Echauri J, et al. SARS-CoV-2 infection in children requiring hospitalization: the experience of Navarra, Spain. *World journal of pediatrics : WJP*. 2020;16(6):614-622. doi:10.1007/s12519-020-00393-x

53. Kim TY, Kim EC, Agudelo AZ, Friedman L. COVID-19 hospitalization rate in children across a private hospital network in the United States: COVID-19 hospitalization rate in children. *Archives de pediatrie : organe officiel de la Societe francaise de pediatrie*. 2021;28(7):530-532. doi:10.1016/j.arcped.2021.07.004

54. Shi T, Pan J, Katikireddi SV, et al. Risk of COVID-19 hospital admission among children aged 5–17 years with asthma in Scotland: a national incident cohort study. *The Lancet Respiratory Medicine*. Published online January 30, 2022. doi:10.1016/S2213-2600(21)00491-4

55. Smith C, Odd D, Harwood R, et al. Deaths in children and young people in England after SARS-CoV-2 infection during the first pandemic year. *Nature Medicine*. 2022;28(1):185-192. doi:10.1038/s41591-021-01578-1

56. Tagarro A, García-Salido A, Martínez EV, Vega-Piris L, Mellado MJ. Low COVID-19 mortality in Spanish children. *The Lancet Child & adolescent health*. 2021;5(6):e24-e25. doi:10.1016/S2352-4642(21)00125-5

Confidential: For Review Only

57. Liu M, Qi Y, Wang W, Sun X. Toward a better understanding about real-world evidence. *European Journal of Hospital Pharmacy: Science and Practice*. 2022;29(1):8-11. doi:10.1136/ejhpharm-2021-003081

58. Nogueira PJ, de Araújo Nobre M, Costa A, et al. The Role of Health Preconditions on COVID-19 Deaths in Portugal: Evidence from Surveillance Data of the First 20293 Infection Cases. *Journal of Clinical Medicine*. 2020;9(8). doi:10.3390/jcm9082368

59. Nogueira PJ, de Araújo Nobre M, Costa A, et al. Reply to Comment on Nogueira, P.J., et al. "The Role of Health Preconditions on COVID-19 Deaths in Portugal: Evidence from Surveillance Data of the First 20293 Infection Cases". J. Clin. Med. 2020, 9, 2368. *Journal of Clinical Medicine*. 2020;9(11). doi:10.3390/jcm9113449

Confidential: For Review Only

TABLE 1. AGE AND SEX OF PEDIATRIC COVID-19 INFECTED CASES, FROM MARCH 2020 TO FEBRUARY 2021 IN PORTUGAL.

		Total n (%)***	Female n (%)**	Male n (%)**	Reported cases per 100 children (%)
N (%)		92051	45583	46453	5.4%
Age	0	1719 (1.9%)	826 (1.8%)	884 (1.9%)	2.0%
	1	3725 (4.0%)	1784 (3.9%)	1940 (4.2%)	4.3%
	2	3692 (4.0%)	1860 (4.1%)	1832 (3.9%)	4.2%
	3	3332 (3.6%)	1575 (3.5%)	1757 (3.8%)	3.8%
	4	3709 (4.0%)	1840 (4.0%)	1869 (4.0%)	4.2%
	Subtotal 1-4 y	14458 (15.7%)	7059 (15.5%)	7398 (15.9%)	4.1%
	5	4023 (4.4%)	1988 (4.4%)	2035 (4.4%)	4.6%
	6	4299 (4.7%)	2128 (4.7%)	2171 (4.7%)	5.1%
	7	4592 (5.0%)	2232 (4.9%)	2360 (5.1%)	5.4%
	8	5058 (5.5%)	2490 (5.5%)	2568 (5.5%)	5.5%
	9	5548 (6.0%)	2709 (5.9%)	2839 (6.1%)	5.7%
	Subtotal 5-9 y	23520 (25.6%)	11547 (25.3%)	11973 (25.8%)	5.3%
	10	5705 (6.2%)	2872 (6.3%)	2833 (6.1%)	5.6%
	11	5726 (6.2%)	2838 (6.2%)	2888 (6.2%)	5.9%
	12	5865 (6.4%)	2928 (6.4%)	2937 (6.3%)	5.8%
	13	6080 (6.6%)	2942 (6.5%)	3137 (6.8%)	6.1%
	14	6449 (7.0%)	3235 (7.1%)	3213 (6.9%)	6.3%
	Subtotal 10-14 y	29825 (32.4%)	14815 (32.5%)	15008 (32.3%)	5,9%
	15	6919 (7.5%)	3402 (7.5%)	3514 (7.6%)	6.5%
	16	7635 (8.3%)	3890 (8.5%)	3744 (8.1%)	7.3%
	17	7975 (8.7%)	4041 (8.9%)	3932 (8.5%)	7.4%
	Subtotal 15-17 y	22529 (24.5%)	11333 (24.9%)	11190 (24.1%)	7.1%

Footnotes: 15 (0.02%) missing values were observed for sex, ***global percentage; **percentage within sex level.

Confidential: For Review Only

TABLE 2. SYMPTOMS ASSOCIATED WITH COVID-19 PER AGE GROUP FROM MARCH 2020 TO FEBRUARY 2021 IN PORTUGAL.

	Age Groups (years)					
	0<br>n (%)	1 - 4<br>n (%)	5 - 9<br>n (%)	10 - 14<br>n (%)	15 - 17<br>n (%)	Total<br>n (%)
Total	1075	8316	11733	14759	11587	47470
Symptoms						
Fever	513 (47.7%)	2846 (34.2%)	2096 (17.9%)	2317 (15.7%)	1965 (17.0%)	9737 (20.5%)
Cough	396 (36.8%)	2397 (28.8%)	1920 (16.4%)	2632 (17.8%)	2771 (23.9%)	10116 (21.3%)
Dyspnea	34 (3.2%)	192 (2.3%)	136 (1.2%)	180 (1.2%)	258 (2.2%)	800 (1.7%)
Rhinorrhoea	376 (35.0%)	2047 (24.6%)	1465 (12.5%)	1927 (13.1%)	1818 (15.7%)	7633 (16.1%)
Odynophagia	13 (1.2%)	364 (4.4%)	1003 (8.5%)	1747 (11.8%)	1831 (15.8%)	4958 (10.4%)
Headache	1 (0.1%)	149 (1.8%)	1351 (11.5%)	2723 (18.4%)	2841 (24.5%)	7065 (14.9%)
Abdominal pain	8 (0.7%)	199 (2.4%)	553 (4.7%)	449 (3.0%)	252 (2.2%)	1461 (3.1%)
Chest pain	0 (0.0%)	11 (0.1%)	59 (0.5%)	148 (1.0%)	214 (1.8%)	432 (0.9%)
Arthralgia	0 (0.0%)	9 (0.1%)	26 (0.2%)	64 (0.4%)	61 (0.5%)	160 (0.3%)
Myalgia	1 (0.1%)	84 (1.0%)	436 (3.7%)	1300 (8.8%)	1731 (14.9%)	3552 (7.5%)
Nausea and vomiting	38 (3.5%)	362 (4.4%)	403 (3.4%)	475 (3.2%)	344 (3.0%)	1622 (3.4%)
Diarrhea	107 (10.0%)	616 (7.4%)	443 (3.8%)	523 (3.5%)	464 (4.0%)	2153 (4.5%)
Convulsions	1 (0.1%)	17 (0.2%)	5 (0.0%)	10 (0.1%)	5(0.0%)	38 (0.1%)
Irritability	19 (1.8%)	43 (0.5%)	9 (0.1%)	9 (0.1%)	5 (0.0%)	85 (0.2%)
General weakness	16 (1.5%)	148 (1.8%)	221 (1.9%)	533 (3.6%)	724 (6.2%)	1642 (3.5%)
Tachycardia	2 (0.2%)	20 (0.2%)	15 (0.1%)	16 (0.1%)	12 (0.1%)	65 (0.1%)

Footnotes: Percentages of patients presenting a specific symptom from patients presenting with the column symptom.

Review Only

TABLE 3. COMORBIDITIES OF THE PEDIATRIC COVID-19 CASES, FROM MARCH 2020 TO FEBURARY 2021, IN PORTUGAL.

		Total<br>n (%) ***	Female<br>n (%) **	Male<br>n (%) **
Total cases with comorbidities*		1593	691	902
Total cases with known comorbidities		1000	427	573
Number of comorbidities	0	18087 (94.9%)	9013 (95.5%)	9074 (94.1%)
	1	943 (4.9%)	401 (4.2%)	542 (6.0%)
	2	47 (0.2%)	20 (0.2%)	27 (0.3%)
	3+	10 (0.1%)	6 (0.1%)	4 (0.0%)
Total		19087	9440	9647
Comorbidities				
	Asthma	753 (47.3%)	316 (45.7%)	437 (48.4%)
	Chronic neurologic / neuromuscular disease	49 (3.1%)	24 (3.5%)	25 (2.8%)
	Diabetes	55 (3.5%)	29 (4.2%)	26 (2.9%)
	Chronic haematological disease	40 (2.1%)	17 (2.5%)	23 (2.5%)
	Chronic neurologic deficit	34 (2.1%)	15 (2.2%)	19 (2.0%)
	Cancer	36 (2.3%)	13 (1.9%)	23 (2.5%)
	Chronic pulmonary disease	42 (2.6%)	17 (2.5%)	25 (2.8%)
	Chronic renal disease	28 (1.8%)	15 (2.2%)	13 (1.4%)
	HIV and other immunodeficiencies	25 (1.6%)	12 (1.7%)	13 (1.4%)
	Liver disease	8 (0.5%)	2 (0.3%)	6 (0.6%)

Footnotes: Results of each morbidity were presented considering the sample size of cases with any reported morbidity (n=1,593). *Total cases with comorbidities, of which 1000 are specified. ***Global percentage; **Percentage within sex level.

nly

TABLE 4. COMORBIDITIES OF THE PEDIATRIC COVID-19 CASES, FROM MARCH 2020 TO FEBURARY 2021, IN PORTUGAL.

		Total n (%)	Female n (%)	Male n (%)
Total cases with comorbidities		1593	691	902
Total cases with known comorbidities		1000	427	573
Number of comorbidities	0	18087 (94.9%)	9013 (95.5%)	9074 (94.1%)
	1	943 (4.9%)	401 (4.2%)	542 (6.0%)
	2	47 (0.2%)	20 (0.2%)	27 (0.3%)
	3+	10 (0.1%)	6 (0.1%)	4 (0.0%)
Total		19087	9440	9647
Comorbiditie*s				
	Asthma	753 (47.3%)	316 (45.7%)	437 (48.4%)
	Chronic neurologic / neuromuscular disease	49 (3.1%)	24 (3.5%)	25 (2.8%)
	Diabetes	55 (3.5%)	29 (4.2%)	26 (2.9%)
	Chronic haematological disease	40 (2.1%)	17 (2.5%)	23 (2.5%)
	Chronic neurologic deficit	34 (2.1%)	15 (2.2%)	19 (2.0%)
	Cancer	36 (2.3%)	13 (1.9%)	23 (2.5%)
	Chronic pulmonary disease	42 (2.6%)	17 (2.5%)	25 (2.8%)
	Chronic renal disease	28 (1.8%)	15 (2.2%)	13 (1.4%)
	HIV and other immunodeficiencies	25 (1.6%)	12 (1.7%)	13 (1.4%)
	Liver disease	8 (0.5%)	2 (0.3%)	6 (0.6%)

Footnotes: *Results of each comorbidity were presented considering the sample size of cases with any reported morbidity (n=1,593).

only

TABLE 45. PEDIATRIC HOSPITAL FROM MARCH 2020 TO DECEMBER 2020 AND PICU ADMISISONS FROM MARCH 2020 TO FEBRUARY 2021 IN PORTUGAL

Hospital admissions from March to December 2020

Age Group	Hospital admissions n (%)	Cases per age group	Hospital admissions per 100 reported cases	Hospital admissions per 10.000 children
0 - 4	228 (47.9%)	10161	2.24	5.2
5-11	112 (23.5%)	18358	0.61	1.7
12-17	136 (28.6%)	22674	0.60	2.2
Total	476 (100%)	51193	0.93	2.8

PICU admissions from March 2020 to February 2021

	Severe COVID-19	MIS-C	Total ICU admissions
PICU admissions (%)	4 (12.5%)	28 (87.5%)	32 (100%)
PICU admissions per 10.000 reported cases	0.43	3.04	3.48
PICU admissions per 10.000 children	0.02	0.16	0.19
Female (%)	3 (75%)	7 (25%)	10 (31.2%)
Male (%)	1 (25%)	21 (75%)	22 (68.8%)
Min age (months)	3	15	
Max age (years)	17	17	
Median (years)	0	10.5	
Comorbidities present (%)	4 (100%)	6 (21.4%)	10 (31.2%)

ew Only

Confidential: For Review Only

Confidential: For Review Only

FIGURE 1. MAP OF COVID-19 PEDIATRIC CASES IN PORTUGAL, BY DISTRICT IN ABSOLUTE VALUE.

Confidential: For Review Only

FIGURE 2. PEDIATRIC COVID-19 CASES PER WEEK

Footnote: 1. First COVID-19 patient diagnosed in Portugal; 2. First national state of emergency; 3. Lockdown easing; 4. Preschool and primary school reopening; 5. End of the school year; 6. Start of the school year; 7. State of emergency with restrictions; 8. Christmas with minimal gathering restrictions; 9. Reintroduction of mandatory remote working, travel restrictions and school closures; 10. Highest daily death toll due to COVID-19 during the pandemic.

Confidential: For Review Only

Confidential: For Review Only

3

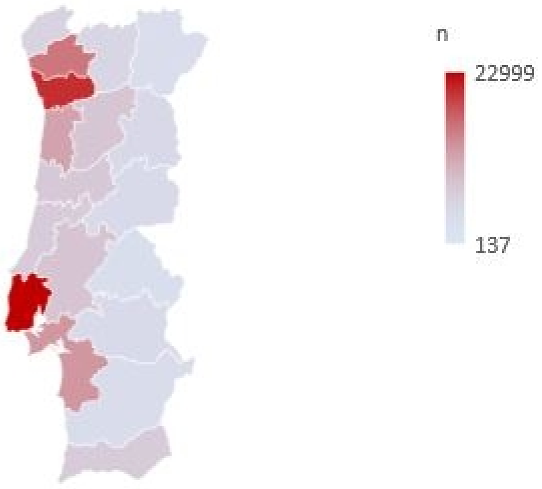

60x56mm (120 x 120 DPI)

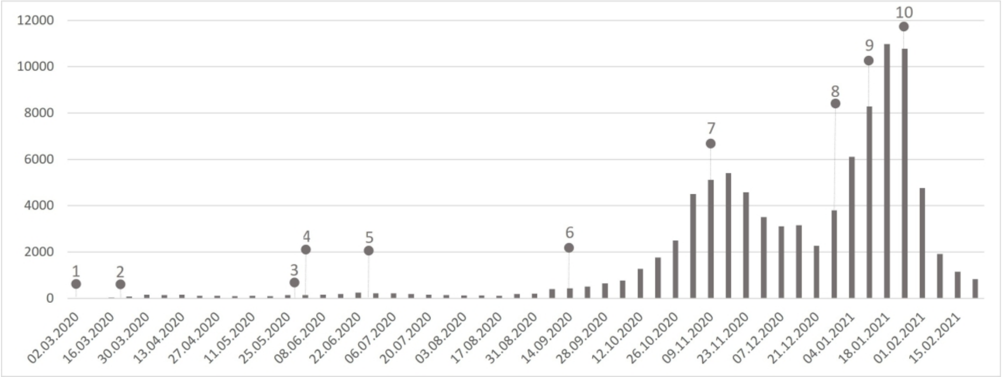

216x82mm (330 x 330 DPI)

SUPPLEMENTARY TABLE 1. PEDIATRIC COVID-19 CASES COUNTRY OF ORIGIN, EXCLUDING PORTUGAL

Country	n(%)
Brasil	2170 (59.2%)
Angola	331 (9.0%)
Cape Verde	205 (5.6%)
Saint Tome and Principe	121 (3.3%)
Guine Bissau	86 (2.3%)
Nepal	75 (2.0%)
Ukraine	72 (2.0%)
Italy	66 (1.8%)
France	62 (1.7%)
Venezuela	49 (1.3%)
Spain	47 (1.3%)
United Kingdom	45 (1.2%)
Romenia	45 (1.2%)
India	33 (0.9%)
Bangladesh	27 (0.7%)
Mozambique	23 (0.6%)
Netherlands	19 (0.5%)
Republic of Moldava	17 (0.5%)
Germany	16 (0.4%)
Paquistan	16 (0.4%)
Belgium	14 (0.4%)
Bulgaria	14 (0.4%)
Colombia	12 (0.3%)
Cuba	9 (0.2%)
Ireland	9 (0.2%)
China	7 (0.2%)
United States of America	7 (0.2%)
Other	67 (1.8%)

r Review Only

SUPPLEMENTARY TABLE 2. SYMPTOMATIC AND ASYMPTOMATIC COVID-19 CASES PER AGE

Years	Asymptomatic n (%)	Symptomatic n (%)	Total	Total reported cases	Missing information n (%)
0	162 (15.1%)	913 (84.9%)	1075	1719	644 (37.5%)
1	387 (15.9%)	2040 (84.1%)	2427	3725	1298 (34.8%)
2	485 (22.6%)	1658 (77.4%)	2143	3692	1549 (42%)
3	527 (28.2%)	1342 (71.8%)	1869	3332	1463 (43.9%)
4	640 (34.1%)	1237 (65.9%)	1877	3709	1832 (49.4%)
5	704 (34.7%)	1325 (65.3%)	2029	4023	1994 (49.6%)
6	769 (36%)	1367 (64%)	2136	4299	2163 (50.3%)
7	825 (36.4%)	1442 (63.6%)	2267	4592	2325 (50.6%)
8	871 (34.7%)	1640 (65.3%)	2511	5058	2547 (50.4%)
9	966 (34.6%)	1824 (65.4%)	2790	5548	2758 (49.7%)
10	918 (33%)	1868 (67%)	2786	5705	2919 (51.2%)
11	892 (31.6%)	1930 (68.4%)	2822	5726	2904 (50.7%)
12	879 (30.2%)	2029 (69.8%)	2908	5865	2957 (50.4%)
13	816 (26.8%)	2229 (73.2%)	3045	6080	3035 (49.9%)
14	869 (27.2%)	2329 (72.8%)	3198	6449	3251 (50.4%)
15	829 (23.5%)	2702 (76.5%)	3531	6919	3388 (49%)
16	778 (19.9%)	3139 (80.1%)	3917	7635	3718 (48.7%)
17	760 (18.4%)	3379 (81.6%)	4139	7975	3836 (48.1%)
Total	13077 (27.5%)	34393 (72.4)	47470	92051	44581 (48.4%)

Footnote: Asymptomatic and symptomatic percentages calculated from all patients with known disease presentation; Missing information percentages calculated from all patients within an age bracket

For Review Only

SUPPLEMENTARY TABLE 3. DISTRIBUITION OF SYMPTOMS OF PAEDIATIC COVID-19 CASES

	Fever	Cough	Rhinorrhea	Dyspneia	Headache	Diarrhoea	Abdominal pain	Odynophagia	Nausea and Vomiting	Arthalgia	Myalgia	Tiredness and weakness	Tachycardia	Chest pain	Irritability	Convulsions
Total*	9737 (46.2%)	10016 (47.5%)	7633 (36.2%)	800 (3.8%)	7065 (33.5%)	2153 (10.2%)	1461 (6.9%)	4958 (23.5%)	1622 (7.7%)	160 (0.8%)	3552 (16.9%)	1642 (7.8%)	65 (0.3%)	432 (2%)	85 (0.4%)	38 (0.2%)
Symptoms**																
Fever		3721 (37,2%)	2902 (38%)	314 (39,3%)	2824 (40%)	1060 (49,2%)	845 (57,8%)	2046 (41,3%)	992 (61,2%)	81 (50,6%)	1615 (45,5%)	752 (45,8%)	52 (80%)	156 (36,1%)	56 (65,9%)	25 (65,8%)
Cough	3721 (38,2%)		4203 (55,1%)	584 (73%)	2724 (38,6%)	790 (36,7%)	430 (29,4%)	2310 (46,6%)	562 (34,6%)	74 (46,3%)	1600 (45%)	752 (45,8%)	30 (46,2%)	261 (60,4%)	40 (47,1%)	18 (42,9%)
Rhinorrhea	2902 (29,8%)	4203 (42%)		366 (45,8%)	1976 (28%)	568 (26,4%)	342 (23,4%)	1746 (35,2%)	415 (25,6%)	68 (42,5%)	1019 (28,7%)	565 (34,4%)	22 (33,8%)	144 (33,3%)	33 (38,8%)	11 (26,2%)
Dyspneia	314 (3,2%)	584 (5,8%)	366 (4,8%)		208 (2,9%)	77 (3,6%)	48 (3,3%)	183 (3,7%)	64 (3,9%)	9 (5,6%)	132 (3,7%)	118 (7,2%)	16 (24,6%)	102 (23,6%)	4 (4,7%)	1 (2,4%)
Headache	2824 (29%)	2724 (27,2%)	1976 (25,9%)	208 (26%)		616 (28,6%)	596 (40,8%)	2106 (42,5%)	578 (35,6%)	99 (61,9%)	2055 (57,9%)	857 (52,2%)	14 (21,5%)	205 (47,5%)	16 (18,8%)	13 (31%)
Diarrhoea	1060 (10,9%)	790 (7,9%)	568 (7,4%)	77 (9,6%)	616 (8,7%)		513 (35,1%)	326 (6,6%)	430 (26,5%)	23 (14,4%)	355 (10%)	220 (13,4%)	4 (6,2%)	45 (10,4%)	16 (18,8%)	6 (14,3%)
Abdominal pain	845 (8,7%)	430 (4,3%)	342 (4,5%)	48 (6%)	596 (8,4%)	513 (23,8%)		351 (7,1%)	436 (26,9%)	24 (15%)	252 (7,1%)	159 (9,7%)	11 (16,9%)	49 (11,3%)	10 (11,8%)	5 (11,9%)
Odynophagia	2046 (21%)	2310 (23,1%)	1746 (22,9%)	183 (22,9%)	2106 (29,8%)	326 (15,1%)	351 (24%)		319 (19,7%)	68 (42,5%)	1080 (30,4%)	460 (28%)	16 (24,6%)	135 (31,3%)	15 (17,6%)	12 (28,6%)
Nausea and Vomiting	992 (10,2%)	562 (5,6%)	415 (5,4%)	64 (8%)	578 (8,2%)	430 (20%)	436 (29,8%)	319 (6,4%)		24 (15%)	296 (8,3%)	228 (13,9%)	13 (20%)	55 (12,7%)	16 (18,8%)	5 (11,9%)
Arthalgia	81 (0,8%)	74 (0,7%)	68 (0,9%)	9 (1,1%)	98 (1,4%)	23 (1,1%)	24 (1,6%)	68 (1,4%)	24 (1,5%)		115 (3,2%)	56 (3,4%)	1 (1,5%)	13 (3%)	3 (3,5%)	0 (0%)
Myalgia	1615 (16,6%)	1600 (16%)	1019 (13,3%)	132 (16,5%)	2055 (29,1%)	355 (16,5%)	252 (17,2%)	1080 (21,8%)	296 (18,2%)	115 (71,9%)		648 (39,5%)	13 (20%)	124 (28,7%)	10 (11,8%)	6 (14,3%)
Tiredness and weakness	752 (7,7%)	752 (7,5%)	565 (7,4%)	118 (14,8%)	857 (12,1%)	220 (10,2%)	159 (10,9%)	460 (9,3%)	228 (14,1%)	56 (35%)	648 (18,2%)		10 (15,4%)	85 (19,7%)	20 (23,5%)	3 (7,1%)
Tachycardia	52 (0,5%)	30 (0,3%)	22 (0,3%)	16 (2%)	14 (0,2%)	4 (0,2%)	10 (0,7%)	16 (0,3%)	13 (0,8%)	1 (0,6%)	13 (0,4%)	10 (0,6%)		4 (0,9%)	5 (5,9%)	0 (0%)
Chest pain	156 (1,6%)	261 (2,6%)	144 (1,9%)	102 (12,8%)	202 (2,9%)	45 (2,1%)	49 (3,4%)	135 (2,7%)	55 (3,4%)	13 (8,1%)	124 (3,5%)	85 (5,2%)	4 (6,2%)		1 (1,2%)	0 (0%)
Irritability	56 (0,6%)	40 (0,4%)	33 (0,4%)	4 (0,5%)	16 (0,2%)	16 (0,7%)	10 (0,7%)	15 (0,3%)	15 (0,9%)	3 (1,9%)	10 (0,3%)	20 (1,2%)	5 (7,7%)	1 (0,2%)		3 (7,1%)
Convulsions	25 (0,3%)	18 (0,2%)	11 (0,1%)	1 (0,1%)	13 (0,2%)	6 (0,3%)	5 (0,3%)	12 (0,2%)	5 (0,3%)	0 (0%)	6 (0,2%)	3 (0,2%)	0 (0%)	0 (0%)	3 (3,5%)	

Footnote: *Percentages calculated from symptomatic patients with specified symptoms, **Percentages calculated from patients with column symptom

view Only

Confidential: For Review Only
